# Evolution and Diversification of *FRUITFULL* Genes in Solanaceae

**DOI:** 10.3389/fpls.2019.00043

**Published:** 2019-02-21

**Authors:** Dinusha C. Maheepala, Christopher A. Emerling, Alex Rajewski, Jenna Macon, Maya Strahl, Natalia Pabón-Mora, Amy Litt

**Affiliations:** ^1^Department of Botany and Plant Sciences, University of California, Riverside, Riverside, CA, United States; ^2^Institut des Sciences de l’Évolution de Montpellier, Université de Montpellier, Centre National de la Recherche Scientifique, Institut de Recherche pour le Développement, École Pratique des Hautes Études, Montpellier, France; ^3^The New York Botanical Garden, Bronx, NY, United States; ^4^Instituto de Biología, Universidad de Antioquia, Medellín, Colombia

**Keywords:** dry fruit, fleshy fruit, fruit development, fruit evolution, *FRUITFULL*, gene duplication, MADS-box transcription factors, Solanaceae

## Abstract

Ecologically and economically important fleshy edible fruits have evolved from dry fruit numerous times during angiosperm diversification. However, the molecular mechanisms that underlie these shifts are unknown. In the Solanaceae there has been a major shift to fleshy fruits in the subfamily Solanoideae. Evidence suggests that an ortholog of *FRUITFULL* (*FUL*), a transcription factor that regulates cell proliferation and limits the dehiscence zone in the silique of *Arabidopsis*, plays a similar role in dry-fruited Solanaceae. However, studies have shown that *FUL* orthologs have taken on new functions in fleshy fruit development, including regulating elements of tomato ripening such as pigment accumulation. *FUL* belongs to the core eudicot *euFUL* clade of the angiosperm *AP1*/*FUL* gene lineage. The *euFUL* genes fall into two paralogous clades, *euFULI* and *euFULII*. While most core eudicots have one gene in each clade, Solanaceae have two: *FUL1* and *FUL2* in the former, and *MBP10* and *MBP20* in the latter. We characterized the evolution of the *euFUL* genes to identify changes that might be correlated with the origin of fleshy fruit in Solanaceae. Our analyses revealed that the Solanaceae *FUL1* and *FUL2* clades probably originated through an early whole genome multiplication event. By contrast, the data suggest that the *MBP10* and *MBP20* clades are the result of a later tandem duplication event. *MBP10* is expressed at weak to moderate levels, and its atypical short first intron lacks putative transcription factor binding sites, indicating possible pseudogenization. Consistent with this, our analyses show that *MBP10* is evolving at a faster rate compared to *MBP20.* Our analyses found that Solanaceae *euFUL* gene duplications, evolutionary rates, and changes in protein residues and expression patterns are not correlated with the shift in fruit type. This suggests deeper analyses are needed to identify the mechanism underlying the change in *FUL* ortholog function.

## Introduction

**FIGURE 1:**
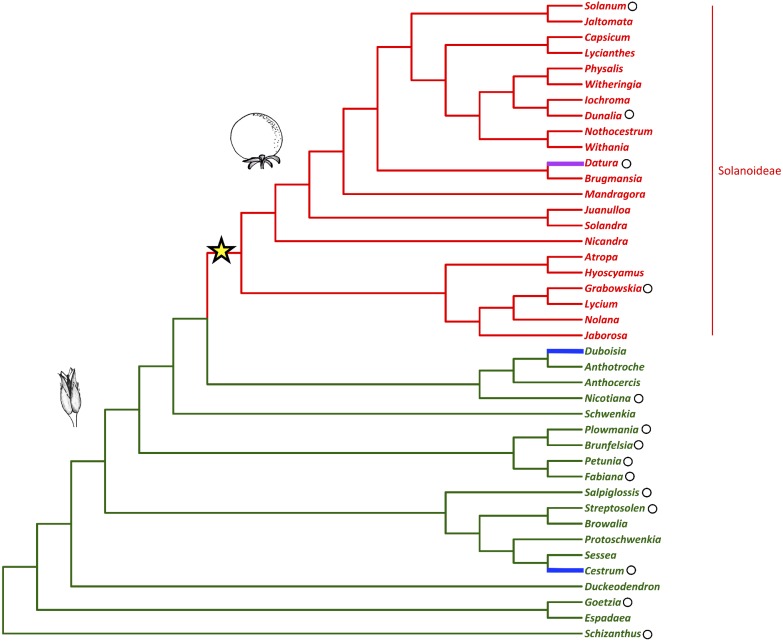
Solanaceae phylogeny with fruit type (dry vs. fleshy) mapped (adapted from Knapp, 2002; Olmstead et al., 2008). The shift to fleshy fruit in the sub-family Solanoideae is indicated with the star. The capsule represents the ancestral fruit-type while the berry represents the generic fruit-type following this shift. The reversal to dry fruit and the independent evolutionary origins of fleshy fruit are highlighted in magenta and blue, respectively. Black circles mark the genera referred to in the text.

Fleshy fruits are agriculturally and economically important plant organs that have evolved from dry fruits many times during angiosperm evolution. However, the genetic changes that are required for this shift to occur are as yet unknown (Bolmgren and Eriksson, 2010). In the agriculturally, pharmacologically, and horticulturally important plant family Solanaceae (nightshades), there was a shift to fleshy fruit in the subfamily Solanoideae from plesiomorphic dry fruit (Figure 1) (Knapp, 2002). In the family two independent transitions to fleshy fruits have also occurred in the genera *Duboisia* (subfamily Anthocercideae) and *Cestrum* (subfamily Cestroideae), as well as a reversal to dry fruit in the genus *Datura* (subfamily Solanoideae) (Knapp, 2002).

Evidence from tomato (*Solanum lycopersicum*, subfamily Solanoideae) indicates that *FRUITFULL* (*FUL*) transcription factors (TFs) have novel functions in fleshy fruit development compared to *Arabidopsis* (Brassicaceae) and *Nicotiana* (Solanaceae, subfamily Nicotianoideae) (Gu et al., 1998; Smykal et al., 2007; Bemer et al., 2012; Shima et al., 2013, 2014; Wang et al., 2014). *FUL* is a MADS-box TF that plays pleiotropic roles in both reproductive and vegetative development in the model plant *Arabidopsis thaliana* (Spence et al., 1996; Gu et al., 1998; Liljegren et al., 2000; Rajani and Sundaresan, 2001; Melzer et al., 2008). *FUL* controls cell proliferation in the fruit valves and spatially limits the formation of the dehiscence zone in the dry silique of *A. thaliana*, enabling the mature fruits to dehisce (Spence et al., 1996; Gu et al., 1998; Liljegren et al., 2000, 2004; Rajani and Sundaresan, 2001). Overexpression of a *Nicotiana tabacum FUL* ortholog in woodland tobacco (*Nicotiana sylvestris*) resulted in indehiscent fruits with reduced lignification at the dehiscence zones, suggesting a role similar to that observed in silique development in *A. thaliana* (Smykal et al., 2007). Several groups have examined the function of *euFUL* genes, the core-eudicot clade to which *FUL* belongs, in tomato (Bemer et al., 2012; Shima et al., 2014; Wang et al., 2014). All studies showed defects in fruit pigmentation during ripening when *FUL* ortholog expression was downregulated, and some studies also suggested roles in ethylene production and pericarp and cuticle thickness (Bemer et al., 2012; Shima et al., 2014; Wang et al., 2014). These data indicate that *euFUL* genes are controlling different processes in dry and fleshy fruits in the Solanaceae.

Early in the diversification of core-eudicots, there was a duplication in the *euFUL* gene clade, which resulted in the *euFULI* and *euFULII* clades (Litt and Irish, 2003; Shan et al., 2007). The *A. thaliana FUL* gene belongs to the *euFULI* clade while its paralog, *AGL79* which plays a role in lateral root development, branching, leaf morphology, and transition to flowering, belongs to the *euFULII* clade (Gao et al., 2018). The *euFULI* clade has duplicated in Solanaceae resulting in two subclades, designated here as *FUL1* and *FUL2*; likewise the *euFULII* clade has two Solanaceae-specific subclades, here designated *MBP10* and *MBP20* (Hileman et al., 2006; Bemer et al., 2012; The Tomato Genome Consortium, 2012). We studied the evolution of *euFUL* genes in Solanaceae to characterize patterns of selection, duplication, and sequence evolution to identify changes that might be correlated with the shift to fleshy fruit. We tested the following hypotheses: (1) following the duplication of *euFUL* genes, there was a relaxation of selection in some or all of the resulting clades that resulted in sequence diversification; (2) changes in amino acid sequences are correlated with the origin of fleshy fruit. Although we found several sites showing changes in amino acid residues that might have resulted in changes in protein function, none of these were associated with the evolution of fleshy fruit. Consistent with our hypothesis, we found that the *FUL1* and *MBP10* genes are evolving at significantly faster rates in comparison to *FUL2* and *MBP20.* In combination with the relatively weak expression of *MBP10* and loss of potential regulatory elements, our data suggest that the *MBP10* lineage may be undergoing pseudogenization.

## Materials and Methods

### Plant Material for Sequencing

Sources of plant and tissue material for sequencing are listed in Table S1 in Supplementary Data Sheet S1. Plants were grown in temperature controlled glasshouses at University of California, Riverside (UCR), The New York Botanical Garden, NY (NYBG), and The University of Antioquia, Colombia (UdeA) or collected from the grounds at UCR and the Universidad de Antioquia or the field at Parque Arvi, Vereda Santa Elena, El Tambo, Colombia.

For ease of reference and to simplify language, throughout the paper, members of Solanoideae, including the dry-fruited *Datura*, will be referred to as “fleshy-fruited species” (rather than “fleshy-fruited species and *Datura*”). Likewise non-Solanoideae, including the fleshy-fruited *Cestrum* and *Duboisia*, will be referred to as “dry-fruited species” (rather than “dry-fruited species and *Cestrum* and *Duboisia*”).

### RNA Isolation, cDNA Synthesis/Library Preparation, and Sequencing

RNA was extracted from fruit, floral/inflorescence or leaf tissue using RNeasy Plant Mini Kits (QIAGEN, Hilden, Germany) according to the manufacturer’s protocol. For *Grabowskia glauca*, *Dunalia spinosa*, *Fabiana viscosa*, and *Salpiglossis sinuata* RNA extractions, lysis buffer RLC was used instead of RLT and 2.5% (w/v) polyvinylpyrrolidone (PVP) was added. The RLT buffer was used for extracting RNA from all other species. RNA quality was checked using a BioSpectrometer Basic (Eppendorf, Hamburg, Germany) and stored at −80°C. cDNA was synthesized using SuperScript III Reverse Transcriptase (Thermo Fisher, San Diego, CA, United States) according to the manufacturer’s protocol and the product was checked by amplifying *ACTIN*. Clade-specific degenerate primers were designed to target specific *euFUL* gene homologs based on conserved regions in Solanaceae *euFUL* gene alignments (Supplementary Table S5). PCR was run for two initial cycles with an annealing temperature between 40 and 45°C followed by 30 cycles at 55°C annealing temperature. The PCR products were visualized on a 1% agarose gel. If multiple amplicon band sizes were present, the annealing temperature of the first two cycles was increased until only one product size was achieved.

PCR products were purified using QIAquick PCR Purification Kit (QIAGEN) according to the manufacturer’s protocol. The purified product was then cloned using TOPO TA Cloning Kit (Life Technologies, Carlsbad, CA, United States) according to the manufacturer’s protocol, and the ligated plasmids were transformed into chemically competent TOP10 strain of *Escherichia coli*. Transformants were plated on LB plates with kanamycin selection (50 μg/mL) coated with 40 μL of 25 mg/mL X-Gal and IPTG, and incubated at 37°C overnight. Individual positive (white) colonies were used as templates in amplification with M13F and M13R primers (Life Technologies, Carlsbad, CA, United States) to identify those colonies with inserts of the expected size between 500 bp and 1 kb. These were grown overnight in 5 mL liquid LB medium supplemented with kanamycin (50 μg/mL) in an incubator-shaker at 250 RPM and 37°C. Plasmids were extracted from the liquid cultures using Plasmid Miniprep Kit (QIAGEN) according to the manufacturer’s protocol, and sequenced using M13 reverse primer at the Institute for Integrative Genome Biology (IIGB) at UCR or Eton Bioscience, Inc. (San Diego, CA, United States).

For library preparation, RNA quality was checked using a Bioanalyzer (Agilent, Santa Clara, CA, United States). RNAseq library preparation was done according to the manufacturer’s Poly(A) mRNA Magnetic Isolation Module protocol for NEBNext Ultra Directional RNA library Prep Kit for Illumina (New England BioLabs, Ipswich, MA, United States). *Cestrum diurnum, C. nocturnum*, and *Schizanthus grahamii* libraries were sequenced on an Illumina NextSeq v2 platform with high-output runs of 75 bp paired-end reads while *Dunalia spinosa, Fabiana viscosa, Grabowskia glauca*, and *Salpiglossis sinuata* libraries were sequenced on an Illumina NextSeq v2 platform with high-output runs of both 75 bp paired-end reads and 150 bp single-end reads at IIGB, UCR. *Nicotiana obtusifolia* libraries were generated at NYBG and sequenced at the Beijing Genomics Institute (Shenzhen, China), and *Brunfelsia australis* and *Streptosolen jamesonii* libraries (Ortiz-Ramírez et al., 2018) were generated at UdeA and sequenced at Macrogen (Korea). All resulting *euFUL* sequences from both degenerate primer PCRs and transcriptomes are listed in Table S2 in Supplementary Data Sheet S1. Individual sequences from PCR-based methods have been deposited in the GenBank (for accession numbers, see Table S1 in Supplementary Data Sheet S1) and transcriptome data for *N. obtusifolia*, *C. diurnum*, *C. nocturnum*, *D. spinosa, F. viscosa, G. glauca*, and *S. grahamii* have been deposited on the SolGenomics network^1^.

### Mining *euFUL* Sequences From *de novo* Transcriptome Assembly and Databases

For transcriptome assembly, raw paired-end reads and single-end reads from Illumina sequencing were first quality trimmed using Trimmomatic v0.36 (Bolger et al., 2014) or TrimGalore (Krueger, 2017) and *de novo* assembled on the UCR High Performance Computing Cluster (HPCC) using the default settings of Trinity v2.4.0 (Grabherr et al., 2011). *Dunalia spinosa, Fabiana viscosa, Grabowskia glauca*, and *Salpiglossis sinuata* libraries were assembled by combining both 75 bp paired-end and 150 bp single-end reads. Each assembled transcriptome was then used to create a custom Basic Local Alignment Tool (BLAST) (Altschul et al., 1990) database. The BLAST database for each species was queried on the HPCC with both blastn and tblastx using all available sequences in our *euFUL* sequence file using a UNIX command line that sequentially matched each sequence in our query file against the database (BLAST^®^ Command Line Applications User Manual, 2008). BLAST analyses were also conducted on the NCBI^2^ (NCBI Resource Coordinators, 2017) and oneKP^3^ (Matasci et al., 2014) databases using *A. thaliana FUL* and various Solanaceae *FUL* homologs as query. Matching output sequences (Table S1 in Supplementary Data Sheet S1) from both transcriptomes assemblies and database mining were further confirmed by compiling a gene tree as described below. We confirmed the accuracy of our sequences using gene specific primers and Sanger sequencing. Unless specified otherwise, all sequences referred to in this manuscript are the full or partial mRNA sequences.

### Gene-Tree Generation

The Multiple Sequence Comparison by Log-Expectation (MUSCLE) (Edgar, 2004) tool was used to align *euFUL* sequences (Supplementary Data Sheet S2). The appropriate model for tree building, GTR+G, was determined with jModelTest 2.0 (Darriba et al., 2012). Ten independent maximum likelihood (ML) analyses starting with random trees were performed using GARLI v2.1 (Genetic Algorithm for Rapid Likelihood Inference) (Bazinet et al., 2014). *euFUL* genes from Convolvulaceae (*Convolvulus*, *Cuscuta* and *Ipomoea* species), which were retrieved from the oneKP database^4^, were designated as the outgroup in each analysis, which meant these sequences were automatically excluded from the ingroup clades. Each ML run was set to terminate when there was no significantly better scoring topology for 20,000 consecutive generations. The ten resulting trees were checked for agreement by calculating the pairwise Robinson–Foulds distance using ‘ape’ and ‘phangorn’ packages on R (Robinson and Foulds, 1981; Paradis et al., 2004; Schliep, 2010; R Core Team, 2018). The tree with the largest ML value was chosen as the starting tree in a bootstrap analysis involving 1,000 replicates. The results of the replicates were summarized and bootstrap values were calculated using SumTrees tool of DendroPy package on Python ver. 2.7 (Python Language Reference, 2010; Sukumaran and Holder, 2010) or Geneious 10.2 (Darling et al., 2010; Kearse et al., 2012).

Any sequences that did not group with any of the subclades were aligned with the paralogs to investigate whether these may have been splice isoforms. Any such isoform was expected to have large insertions/deletions at splice junctions. None were noted.

### Selection Pressure Analysis

The CODEML program within the Phylogenetic Analysis by Maximum Likelihood (PAML) (Yang, 1997) v 1.3^5^ software package was run on the HPCC at UCR to analyze the selection pressure acting on *euFUL* genes. These analyses were performed to test if different gene lineages as well as sub-groups within those lineages were evolving at significantly different rates. Further scenarios were considered in which each gene, the transition branches from dry to fleshy fruit trait, or specific sites in the sequences were tested for significantly different rates of evolution. Model 0 (M0) was used to estimate a single evolutionary rate for all genes when the clades being analyzed encompassed the entire dataset. Model 2 (M2) was used when two groups encompassing the entire data set have different rates or when two groups that are being compared do not encompass the entire data set. In the latter case, the two clades being compared were grouped together to obtain a single evolutionary rate in comparison to the rate for the remaining data (background). This single rate for the two clades grouped together was then compared to the rates for each clade separately to determine if the separate rates were significantly different from the combined rate. The test statistic, 2ΔL (twice the difference of the resulting log-likelihood values), and the degrees of freedom (df), were then used in chi-squared tests to check for statistical significance. In any comparison where the *P*-value was less than 0.05, the second hypothesis was considered to have the better fit than the first, implying there is statistical power to support that the gene clades are evolving at different rates. Since Solanaceae has a well-supported phylogeny (Olmstead et al., 2008; Särkinen et al., 2013), for PAML analyses, the branches of the gene-tree described above were adjusted to match the phylogenetic relationships of the species included in the analysis. In the *euFUL* gene groups that are evolving faster, sites undergoing positive selection were analyzed using mixed effects model of evolution (MEME)^6^ (Murrell et al., 2012).

The gene alignments for the *euFUL* subclades that are evolving at statistically significantly faster than the other subclades were translated using AliView (Larsson, 2014). In these protein alignments, the sites that changed from hydrophilic to hydrophobic or vice versa were identified manually. Those changes that might have been functionally deleterious versus those that might have been neutral were identified using the PROVEAN Protein tool^7^ (Choi, 2012; Choi et al., 2012; Choi and Chan, 2015).

MADS (M), intervening/interacting (I) and keratin-like (K) domains of the proteins were identified using a published MADS-box protein model (Kaufmann et al., 2005).

The structure of M, I, and K domains of tomato FUL1 and MBP10 were predicted using PHYRE2 server^8^ (Kelley et al., 2015).

### *MBP10/MBP20* Synteny and Intron Analyses

One-million-base-pair regions surrounding tomato *MBP10* and *MBP20* were analyzed for synteny using the progressive Mauve alignment tool on Geneious 10.2^9^ (Darling et al., 2010; Kearse et al., 2012).

Putative TF binding site searches for *MBP10* and *MBP20* first introns were done using PROMO 3.0^10^ at a maximum matrix dissimilarity rate of zero (Messeguer et al., 2002; Farré et al., 2003).

### Solanaceae *euFUL* Expression Analysis

The expression patterns of *euFUL* genes were analyzed using RT-PCR data for *Solanum pimpinellifolium* organs, and transcriptome data from this study for five stages of fruit development in *S. pimpinellifolium* and tomato following stages identified by Gillaspy et al. (1993) and Tanksley (2004). Additional expression data were obtained from the eFP browser^11^ for tomato, *S. pimpinellifolium*, potato (*S. tuberosum*) (Massa et al., 2011; Potato Genome Sequencing Consortium et al., 2011; The Tomato Genome Consortium, 2012) and from the Gene Expression Atlas^12^ for *Nicotiana benthamiana* (Nakasugi et al., 2014), and other publications (Hileman et al., 2006; Burko et al., 2013).

The TF binding sites for the 2 kb and 5 kb regions upstream of the *euFUL* gene transcription start sites of tomato (GCF_000188115.4) (The Tomato Genome Consortium, 2012), potato (GCF_000226075.1) (Potato Genome Sequencing Consortium et al., 2011) and *N. sylvestris* (GCA_000393655.1) (Sierro et al., 2013) were predicted using PlantPAN 2.0^13^ (Chang et al., 2008). Due to the limitations of available contig length, the longest promoter region used for *N. sylvestris MBP10* was 3.3 kb.

## Results

### Solanaceae Have Four Clades of *euFUL* Genes

Our analysis consisted of 106 sequences from 45 species in 26 genera obtained from direct amplification, transcriptomes, and online genomic databases (Table S1 in Supplementary Data Sheet S1). Of these, 64 sequences belonged to species from the Solanoideae, characterized by the derived fleshy fruit, whereas the other 42 sequences were from species with the ancestral dry-fruit trait. We designated *euFUL* genes from Convolvulaceae, the sister-group of Solanaceae, as the outgroup (Stefanoviæ et al., 2003). For many species in the analysis, we have an incomplete set of paralogs; however, we had substantial and diverse representation from across the phylogeny, which allows us to test hypotheses regarding the evolution of this gene lineage in Solanaceae.

**FIGURE 2:**
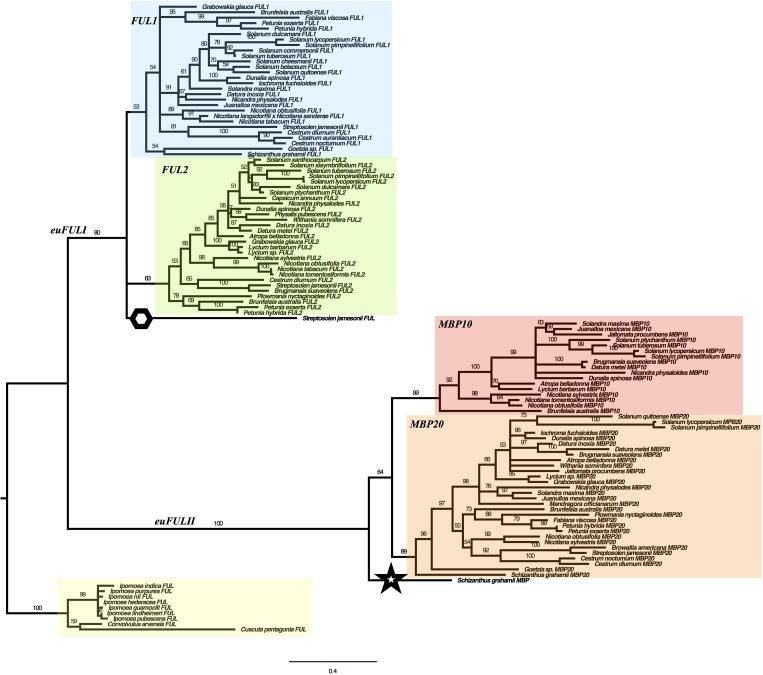
Solanaceae *euFUL* Maximum Likelihood gene tree. *FUL1*, *FUL2*, *MBP10*, and *MBP20* clades are colored in blue, green, red, and orange, respectively. A hexagon is placed next to the *Streptosolen* gene that is sister to *FUL1* and *FUL2* clades, and a star is placed next to the *Schizanthus* gene that is sister to the *euFULII* clade. The Convolvulaceae outgroup is highlighted in yellow. The numbers on the branches indicate the bootstrap support.

We used maximum likelihood methods (Garli v2.1) (Bazinet et al., 2014) to reconstruct the relationships of Solanaceae *euFUL* genes (Figure 2). The resulting tree shows two major lineages of *euFUL* genes, with 80% and 100% bootstrap support, respectively, that correspond to the previously identified core eudicot *euFULI* and *euFULII* lineages (Litt and Irish, 2003; Shan et al., 2007). A Solanaceae whole-genome triplication has been proposed (The Tomato Genome Consortium, 2012; Albert and Chang, 2014; Vanneste et al., 2014; Bombarely et al., 2016), which would suggest that all Solanaceae should have three *euFULI* and three *euFULII* genes. However, others have suggested a duplication (Blanc and Wolfe, 2004; Schlueter et al., 2004; Song et al., 2012). Our data and other studies, as well as searches of the tomato genome have shown that tomato has four *euFUL* genes: two *euFULI* and two *euFULII* (Hileman et al., 2006; Bemer et al., 2012; The Tomato Genome Consortium, 2012) instead of the six predicted by a triplication. Additional genome sequencing (e.g., potato, *Capsicum annuum*) (Potato Genome Sequencing Consortium et al., 2011; Hulse-Kemp et al., 2018), transcriptome sequencing, and PCR-based analyses (this study) have also found two *euFULI* and two *euFULII* genes. This suggests the loss of one paralog from each of the *euFULI* and *euFULII* clades following a whole-genome triplication (The Tomato Genome Consortium, 2012; Albert and Chang, 2014; Vanneste et al., 2014; Bombarely et al., 2016) or, alternatively one or more duplication events (Blanc and Wolfe, 2004; Schlueter et al., 2004; Song et al., 2012).

For the purposes of this paper, we will refer to the *euFULI* and *euFULII* subclades by the name currently used for the tomato gene in each subclade (Hileman et al., 2006; Bemer et al., 2012). Thus, the two *euFULI* subclades will be referred to as the *FUL1* and *FUL2* clades, and the *euFULII* subclades will be referred to as the *MPB10* and *MBP20* subclades (Figure 2). In our gene tree, while the *FUL2, MBP10*, and *MBP20* clades had high bootstrap support of 83, 99 and 89%, respectively, the *FUL1* clade had only 53% support (Figure 2). A single gene from *Streptosolen* grouped sister to the *FUL1* and *FUL2* clades, while a gene from *Schizanthus*, one of the earliest diverging genera (Olmstead et al., 2008; Särkinen et al., 2013), grouped as sister to the *euFULII* clade. To confirm the above were not artifacts, we re-assembled the *Streptosolen* transcriptome while searching for reads supporting the gene contig, and amplified the *Schizanthus* sequence using gene-specific primers.

The presence of both *FUL1* and *FUL2* genes in species from across the phylogeny is consistent with the event that produced these two clades being part of a family-wide, whole-genome duplication or triplication (Blanc and Wolfe, 2004; Schlueter et al., 2004; Song et al., 2012; The Tomato Genome Consortium, 2012; Albert and Chang, 2014; Vanneste et al., 2014; Bombarely et al., 2016). However, we did not find a *FUL2* ortholog in *Schizanthus*, using transcriptome data, or *Goetzia*, using PCR. These two genera are among the earliest diverging in the family (Olmstead et al., 2008; Särkinen et al., 2013), and are the earliest that we sampled. This raises the possibility that the *FUL1/FUL2* clades resulted from a duplication that occurred following the diversification of *Schizanthus* and *Goetzia.* In addition, although we obtained *MBP10* sequences from *Nicotiana* and most of the genera that diversified subsequently (Figure 1 and Figure S2 in Supplementary Data Sheet S1), we did not find members of the *MBP10* clade in genera that diverged prior to *Brunfelsia*. This suggests that the *MBP10* and *MBP20* subclades were produced by a duplication that occurred later in Solanaceae diversification, after the *euFULI* duplication and any proposed family-wide whole-genome events.

### The *euFULII* Clades Are the Result of a Tandem Gene Duplication

**FIGURE 3:**
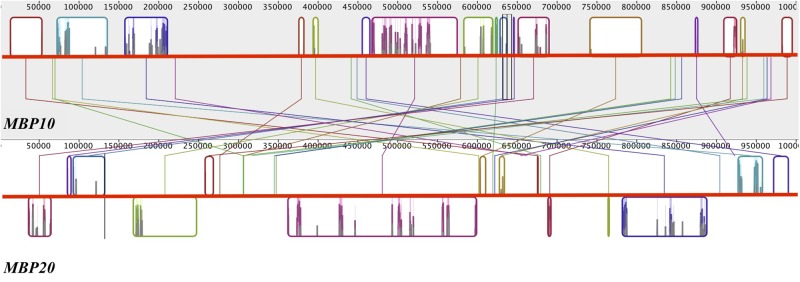
Reverse synteny of the regions surrounding *MBP10* and *MBP20* on tomato chromosome 2. The gray block at the top contains the 1 Mbp region surrounding *MBP10* and the white block at the bottom contains the 1 Mbp region surrounding *MBP20*. A colored box in one block is homologous to a box with the identical color in the other block. *MBP10* and *MBP20* genomic sequences are in the center homologous region of the respective block. In *MBP20*, the boxed regions below the red horizontal line are in reverse orientation to the corresponding homologous regions in *MBP10*.

To investigate the nature of the *MBP10/MBP20* duplication, we mapped the location of the four *euFUL* paralogs to the genome of cultivated tomato. *FUL1* and *FUL2* are located on chromosomes 6 and 3, respectively, consistent with their origin from a whole genome multiplication. By contrast, *MBP10* and *MBP20* are both located on chromosome 2, about 14.3 million base pairs apart (Figure 3). The location of both *euFULII* genes on the same chromosome, and the presence of only one ortholog in early diverging species, support the hypothesis that these paralogs may be the result of a tandem gene duplication. Moreover, comparing a 1-million-base-pair region surrounding both *MBP10* and *MBP20* shows synteny, further supporting a tandem duplication (Figure 3). Annotations indicate that these syntenic zones contain 17 homologous regions. The regions that show homology are located on the opposite sides of *MBP10* and *MBP20*, suggesting an inversion of the tandemly duplicated region.

Although we recovered an *MBP10*-clade member in *Brunfelsia australis* using transcriptome analysis, we were unable to amplify this gene from leaf or floral tissue of *Fabiana* or *Plowmania*, genera that are most closely related to *Brunfelsia* (Figure 1 and Figure S2 in Supplementary Data Sheet S1). In addition, *Petunia* is also a member of the clade that includes *Brunfelsia*, and searches of the published *Petunia* genomes (Bombarely et al., 2016) also failed to turn up an *MBP10*-clade member. However, the *Brunfelsia* sequence in our analysis, obtained from transcriptome data, falls in the expected place in the phylogeny, and we confirmed the presence of *MBP10* transcript in *Brunfelsia floribunda* floral RNA. This suggests that the *MBP10/MBP20* duplication occurred before the divergence of the *Brunfelsia*/*Fabiana*/*Petunia*/*Plowmania* clade but the *MBP10* paralog was lost in *Fabiana*, *Petunia* and *Plowmania*.

### *MBP10* Has a Short First Intron With No TF Binding Sites

A long first intron ranging from 1 to 10 kb, with multiple potential TF binding sites, is a general feature of *FUL* homologs (Table 2) (Takumi et al., 2011). By contrast, *MBP10* has a short first intron of about 80 bp in both cultivated tomato and its closest wild relative, *S. pimpinellifolium*, and about 110 bp in *Nicotiana obtusifolia* (Table 2). The expression of most *euFUL* genes is strong across nearly all vegetative and reproductive organs (Ferrándiz et al., 2000; Shchennikova et al., 2004; Kim et al., 2005; Hileman et al., 2006; Bemer et al., 2012; Pabón-Mora et al., 2012, 2013; Scorza et al., 2017); however, diverse analyses using both quantitative and non-quantitative methods indicate that *MBP10* expression is relatively weak in tomato, *S. pimpinellifolium*, and *N. obtusifolia* in most organs (Massa et al., 2011; Potato Genome Sequencing Consortium et al., 2011; The Tomato Genome Consortium, 2012; Nakasugi et al., 2014), however, some studies have suggested moderate expression in leaves (Figure S1 in Supplementary Data Sheet S1 and unpublished data). To determine if the short first intron lacks putative TF binding sites, we searched the first intron of *MBP10* and *MBP20* in tomato (Promo v3.0) (Messeguer et al., 2002; Farré et al., 2003). We found that the first intron of *MBP10* contains no putative TF binding sites, while that of *MBP20* contains 88 putative TF binding sites for eight different TFs. These TFs belong to five main families (Figure S3 in Supplementary Data Sheet S1): MYB (MYB2, C1), HSF (HSF1), Dof (Dof1, MNB1a, PBF), WRKY (SPF1) and MADS-box (SQUA). A similar situation was observed for *Nicotiana obtusifolia*, which had 133 putative binding sites in the first intron of *MBP20* for a similar array of TFs, while *MBP10* had only four such sites. In addition, we searched the first intron of *AGL79*, the *euFULII* paralog of *FUL* in *A. thaliana*, and found 49 putative binding sites, also for similar TFs and TF families. This suggests a loss of regulatory motifs in *MBP10*.

### *FUL1* and *MBP10* Genes Are Evolving at a Faster Rate Than *FUL2* and *MBP20*

Using the Solanaceae *euFUL* sequence data (Table S1 in Supplementary Data Sheet S1), we conducted selection pressure analyses (PAML v1.3) (Yang, 1997) to investigate if there was a shift in evolutionary rate following the *FUL1/FUL2* or *MBP10/MBP20* duplication. Selection pressure (ω) acting upon different *euFUL* gene subclades was calculated as the ratio of the rate of non-synonymous substitutions to the rate of synonymous substitutions (dN/dS) (Yang, 1997; Yang and Nielsen, 2000). An ω value of less than 1 means the coding regions are under purifying selection and that protein function is conserved. By contrast, an ω of more than 1 means that the coding regions are under diversifying selection (Yang and Nielsen, 2000). This is interpreted as allowing potential divergence in protein function (Torgerson et al., 2002; Almeida and Desalle, 2009). The nucleotide alignments we used in these analyses excluded the C-termini for all sequences except for those in the *FUL2* clade, due to the high variability of this region, which prevents reliable alignment.

Our results indicate that all Solanaceae *euFUL* gene clades are undergoing purifying selection (ω ≤ 0.20; Table 1 and Table S3 in Supplementary Data Sheet S1), suggesting conservation of function. The two main lineages, *euFULI* (ω = 0.13) and *euFULII* (ω = 0.16) are evolving at statistically indistinguishable rates. However, within the *euFULI* clade, genes of the *FUL1* clade are evolving at a significantly higher rate (ω = 0.17) compared to those of the *FUL2* clade (ω = 0.11). Within the *euFULII* clade, *MBP10* genes are also evolving at a significantly higher rate (ω = 0.19) compared to *MBP20* (ω = 0.15). Comparing each clade against all other clades showed that *FUL2* ortholog sequences are the most conserved while *MBP10* ortholog sequences have the weakest purifying selection rates, followed by *FUL1*, implying the possibility of diversifying functions in the latter two subclades (Table 1 and Table S3 in Supplementary Data Sheet S1). None of the gene groups showed a change in evolutionary rates in comparisons between dry- and fleshy-fruited species (Table S3 in Supplementary Data Sheet S1).

**Table 1:** Evolutionary rates of *euFUL* gene clades that are evolving at statistically different rates.

Comparison	Model	ω_0_	ω_1_	ω_2_	2ΔL	*df*	*P*-value
*FUL1* vs. *FUL2*	M2_A_ (ω_0_: background; ω_1_: *FUL1* and *FUL2*)	0.1577	0.1311	_	15.5040	1	0.0001
	M2_B_ (ω_0_: background; ω_1_: *FUL1*; ω_2_: *FUL2*)	0.1577	0.1710	0.1064			
*MBP10* vs. *MBP20*	M2_A_ (ω_0_: background; ω_1_: *MBP10* and *MBP20*)	0.1311	0.1577	_	7.0291	1	0.0080
	M2_B_ (ω_0_: background; ω_1_: *MBP10*; ω_2_: *MBP20*)	0.1279	0.1939	0.1514			
*FUL1* vs. other *euFUL*	M0 (ω_0_: all branches)	0.1423	_	_	5.3906	1	0.0001
	M2 (ω_0_: FUL_1_; ω_1_: other euFUL)	0.1706	0.1344	_			
*FUL2* vs. other *euFUL*	M0 (ω_0_: all branches)	0.1423	_	_	19.3663	1	0.0000
	M2 (ω_0_: *FUL2*; ω_1_: other *euFUL*)	0.1065	0.1622	_			
*MBP10* vs. other *euFUL*	M0 (ω_0_: all branches)	0.1423	_	_	8.7258	1	0.0031
	M2 (ω_0_: *MBP10*; ω_1_: other *euFUL*)	0.1943	0.1348	_			

*2ΔL, test statistic (two times the difference of log-likelihood values); df, degrees of freedom. When the groups being compared did not encompass the entire data set, Model 2 required a two-step analysis (see the section “Materials and Methods”). In this case subscript A and B were used to distinguish the analyses.*

### Rapidly Evolving Sites Are in Regions Responsible for Protein Complex Formation

We further analyzed the sequences to identify changes at individual amino acid sites, specifically those that involved a change between polar/charged and non-polar, that might have resulted in a change in protein conformation and function and that were correlated with the change from dry to fleshy fruit. The *euFUL* genes belong to the Type II MADS-domain containing proteins, which are characterized by a MADS (M) domain, which functions in DNA binding and DNA-protein dimer specificity, an intervening/interacting (I) domain that also has a role in dimer specificity, a keratin-like (K) domain important for protein–protein interactions, and a C-terminal (C) domain, implicated in protein-multimerization, transcription activation, and additional functions (Cho et al., 1999; Heijmans et al., 2012). The C-termini were excluded from this analysis. We selected comparisons in which our results showed two gene groups evolving at significantly different rates (e.g., *FUL1* vs. *FUL2*; Table 1 and Table S3 in Supplementary Data Sheet S1). In the faster evolving group, we searched for sites in the M, I, and K regions that are undergoing diversifying selection (>1) using mixed effects model of evolution (MEME) (see footnote 6)^14^ (Murrell et al., 2012). The results (Figure S4 in Supplementary Data Sheet S1) suggest that sites undergoing diversifying selection are located mainly between amino acids 90 and 180 (out of ∼210 amino acids in the protein). This region corresponds to the K domain (∼90 to ∼180 amino acids) (Kaufmann et al., 2005). In comparison, the M (∼1 to ∼60 amino acids) and the I domains (∼60 to ∼90 amino acids) had relatively few sites undergoing diversifying selection. Since these TFs function in complexes with other MADS-domain proteins as well as other proteins, novel interactions made possible by amino acid changes in this region might lead to changes in transcriptional activity.

The K domain had 14 sites undergoing diversifying selection in the FUL1 proteins and four of those showed a change in polarity (Figure S4 in Supplementary Data Sheet S1). Of those four, a site that corresponds to the 153rd residue in the tomato protein had negatively charged glutamate (E) in most of the non-Solanoideae (mainly dry-fruited) species (11 out of 15 sequences) while all Solanoideae (mainly fleshy-fruited) species had a non-polar residue: valine (V; 13 species) or methionine (M; 1 species) (Figure S5 in Supplementary Data Sheet S1). This change was due to a single nucleotide change from an A to T in the former and G to A in the latter. All other changes in FUL1 proteins that result in a change in charge appeared to be reversible, and none were correlated with the phylogeny nor with phenotypic changes. We used the PROVEAN tool on all four K-domain sites that showed a change in charge to predict whether these transitions were likely to be deleterious or neutral (Choi, 2012; Choi et al., 2012; Choi and Chan, 2015). Two of these sites, one with a histidine (H) to glutamine/asparagine (Q/N) shift at the 95th residue, and one with a lysine (K) to glutamine/threonine (Q/T) shift at the 157th residue (Figure S5 in Supplementary Data Sheet S1), were predicted to be functionally deleterious while the other two sites, including the 153rd residue with E to V change, were predicted to be neutral. There were five rapidly changing sites in the M domain and six sites undergoing positive selection in the I domain of FUL1. None of the sites in the M domain showed a change in polarity. Only one site in the I domain showed a change in polarity, but this site was predicted to be neutral functionally. MBP10 proteins had 20 sites undergoing diversifying selection in the K domain, only 1 such site in the M domain and 3 in the I domain (Figures S4, S5 in Supplementary Data Sheet S1). Of these, only three sites in the I domain showed a change in charge, all of which were also predicted not to have a negative effect on function.

### Solanaceae *euFULI* and *euFULII* Homologs May Have Experienced Distinct Mechanisms of *Cis*-Regulatory Evolution

**FIGURE 4:**
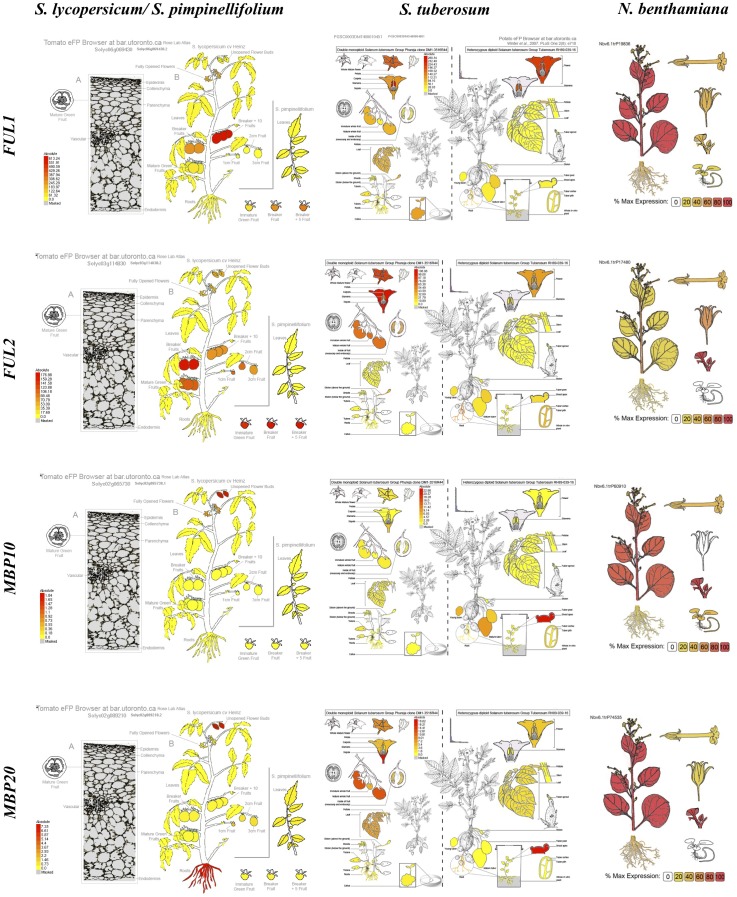
The *euFUL* expression profiles in *Solanum lycopersicum*, *S. pimpinellifolium*, *S*. *tuberosum*, from eFP browser (http://bar.utoronto.ca), and *Nicotiana benthamiana*, from the Gene Expression Atlas (http://benthgenome.qut.edu.au) data.

We compared *euFUL* expression data for the cultivated and wild tomato species, potato and *Nicotiana benthamiana* to identify any patterns that might be the result of changes in the regulatory regions following the duplications of these genes. Not all data from online sources were comparable across species, as different studies included different organs and developmental stages in their analyses, limiting cross-species comparisons. The analysis shows similar spatial expression patterns for *FUL1* and *FUL2* (Figure 4 and Figure S1 in Supplementary Data Sheet S1). These two paralogs are broadly expressed in leaves, flowers and fruits of tomato, potato, and tobacco. Although the eFP browser data (Figure 4) shows no expression for *FUL1* and *FUL2* in tomato leaves, our RT-PCR data (Figure S1 in Supplementary Data Sheet S1) and previous publications (Hileman et al., 2006; Burko et al., 2013) show expression of all four *euFUL* homologs in these organs. Both *euFULI* genes are expressed relatively weakly in the roots of tomato, potato, and tobacco (Massa et al., 2011; Potato Genome Sequencing Consortium et al., 2011; The Tomato Genome Consortium, 2012; Nakasugi et al., 2014) (Figure 4 and Figure S1 in Supplementary Data Sheet S1). Although spatial domains of expression are similar for the *euFULI* genes, they differ in temporal expression over the course of fruit developmental stages in tomato. Although both *FUL1* and *FUL2* are expressed in the fruits of all species, in tomato *FUL2* is highly expressed during the early stages of fruit development and then tapers off, whereas *FUL1* expression increases with time (Figure 4 and Figure S1 in Supplementary Data Sheet S1).

In comparison to the *euFULI* genes, the two *euFULII* paralogs show more striking differences in spatial expression at the organ level (Figure 4 and Figure S1 in Supplementary Data Sheet S1), and also between species. In all species for which expression is reported, *MBP10*, alone among the *euFUL* genes in Solanaceae, is not expressed in fruits, or is expressed at barely detectable levels. In tomato, *MBP20* is expressed strongly in roots while *MBP10* is not. By contrast, in potato tubers, *MBP10* expression is high and *MBP20* is not expressed (Figure 4). The online sources and our RT-PCR data also show subtle intra-specific differences in expression between *MBP10* and *MBP20* in flowers (Figure 4 and Figure S1 in Supplementary Data Sheet S1). In addition, our RT-PCR data show that *MBP10* is expressed relatively weakly in petals and stamens in tomato while *MBP20* is expressed throughout the flower (Figure S1 in Supplementary Data Sheet S1). However, these differences seem to be a matter of expression intensity in comparison to the more striking contrasts seen in roots, tubers, and fruits.

The types of differences in expression between *FUL1* and *FUL2* versus *MBP10* and *MBP20* might be due to differences in the regulatory environment as a result of the different ways in which these duplicates arose. A tandem duplication and inversion may have disrupted regulatory regions in ways that would not be associated with a whole genome duplication or triplication (Tanimoto et al., 1999; Kmita et al., 2000; Vogel et al., 2009; Lupiáñez et al., 2015; Puig et al., 2015). To investigate this, we searched for putative TF binding sites in the promoter regions (2 and 5 kb upstream from the transcription start site) of *euFUL* genes in tomato, potato, and woodland tobacco to compare the differences between the pairs of paralogs (Table S4 in Supplementary Data Sheet S1). Woodland tobacco was used rather than *N. benthamiana* since relatively longer promoter sequence lengths for *euFUL* genes were available for this genome assembly (Sierro et al., 2013). Despite this, the maximum available promoter length for *NsMBP10* was about 3.3 kb. We found that the differences in types and numbers of predicted TF binding sites between *FUL1* and *FUL2* were comparable to the differences between *MBP10* and *MBP20* (Table S4 in Supplementary Data Sheet S1). Nonetheless we did find some differences that may underlie observed differences in expression between paralogs. Some of these differences were presence/absence of binding sites for a particular TF, and some were in the number and distribution of sites. Putative binding sites for AUXIN RESPONSE FACTORS (ARF) were absent from the tomato *FUL2* promoter while they were present in the promoters of all other *euFUL* genes in all species examined. Only *FUL2* in tomato, *FUL1* in potato, and *MBP10* in woodland tobacco contained binding sites for STOREKEEPER (STK). ETHYLENE INSENSITIVE 3 (EIN3) has three sites in tomato *FUL1* and five in tomato *FUL2*, but the distribution of the sites differs. In *FUL1*, there are no sites within 2 kb of the coding sequence, and three within 5 kb, whereas in *FUL2* there is one site in the 2 kb region and four in the full 5 kb region. In woodland tobacco, there are three EIN3 sites in *FUL1*, all of which are within the 2 kb region, and only one in *FUL2*, which is located between 2 and 5 kb. These types of differences may underlie observed differences in expression.

## Discussion

### Solanaceae *euFUL* Gene Tree Shows the History of Duplications in This Lineage

In Solanaceae, there has been a major shift to fleshy fruit in the Solanoideae (Knapp, 2002). However, we do not know the molecular basis of this economically and ecologically important evolutionary event. *FUL* negatively regulates lignification in the dehiscence zone in the dry silique of *A. thaliana*, and functions in cauline leaf development, the transition to flowering and determinacy (Spence et al., 1996; Gu et al., 1998; Liljegren et al., 2000, 2004; Rajani and Sundaresan, 2001; Melzer et al., 2008). Studies of *FUL* ortholog function across the angiosperms have shown that it is labile, and orthologs have acquired diverse roles over evolutionary time. *VEGETATIVE 1* (*VEG1*), an ortholog of *FUL* in pea (*Pisum sativum*), is involved in secondary inflorescence meristem identity (Berbel et al., 2012). *AGAMOUS-like 79* (*AGL79*), the *A. thaliana euFULII* paralog of *FUL*, is mainly expressed in the root and has functions in lateral root development and may also play a role function in leaf shape, leaf number, branching, and time to flowering (Gao et al., 2018). However, the overexpression of an *AGL79* ortholog from snapdragon (*Antirrhinum majus*) in *A. thaliana* resulted in indehiscent siliques, suggesting a role more similar to *A. thaliana FUL* (Müller et al., 2001). Evidence suggests that in tomato, one of the *AGL79* orthologs, *MBP20*, plays a role in leaf development (Burko et al., 2013). *VERNALIZATION 1* (*VRN1*) genes, which are *FUL-like* orthologs in grass species such as wheat (*Triticum* spp.) and barley (*Hordeum vulgare*), function in the vernalization response (Preston and Kellogg, 2008). Evidence to date, therefore, suggests that *euFUL* function is labile, and has changed substantially in different plant lineages during the course of angiosperm evolution. Thus it is not surprising to find a change in function of *euFUL* orthologs in Solanaceae.

There is evidence to suggest that Solanaceae *euFUL* orthologs play a role similar to that of *A. thaliana FUL* in the development of dry dehiscent fruits (Smykal et al., 2007). However, studies suggest that in the fleshy fruit of Solanoideae, *FUL* orthologs play roles in pigmentation as well as ethylene response, cell wall modification, glutamic acid degradation, volatile production, and pericarp and cuticle thickness (Bemer et al., 2012; Shima et al., 2014; Wang et al., 2014). To determine if we could identify changes in *euFUL* sequences or selection that might shed light on this change in function, we analyzed *euFUL* gene evolution in Solanaceae.

We performed a maximum likelihood phylogenetic analysis (Garli v2.1) (Bazinet et al., 2014) on a data set that consisted of 106 Solanaceae members of the *euFUL* gene lineage (Litt and Irish, 2003; Shan et al., 2007), which we obtained through amplification and sequencing (37 sequences), generating transcriptome sequence data (29 sequences), or mining databases (40 sequences). As outgroup we used 10 *euFUL* genes from Convolvulaceae, the sister family to Solanaceae (Figure 2 and Supplementary Table S1) (Stefanoviæ et al., 2003). The resulting tree shows the two major clades of core-eudicot *euFUL* genes, the *euFULI* and *euFULII* lineages (Litt and Irish, 2003; Shan et al., 2007). Within each of these clades there is evidence of a Solanaceae-specific duplication, resulting in two subclades in each lineage. Within each subclade, the order of branches correlates well with the topology of the Solanaceae phylogeny (Olmstead et al., 2008; Särkinen et al., 2013); discrepancies at the genus level are likely due to the short length of some sequences and sequence divergence in some taxa. Each of the subclades includes orthologs from both fleshy- and dry-fruited species, indicating that the subclade duplications preceded the origin of fleshy fruit.

Although duplications in these genes are common (Litt and Irish, 2003; Preston and Kellogg, 2007; Pabón-Mora et al., 2013), we did not find significant evidence of taxon-specific duplications. We did, however, find two genes that did not fall into a specific subclade. A third *Streptosolen* gene grouped sister to the rest of the *euFULI* clade (76% identity among the three *Streptosolen* genes), potentially the result of a taxon-specific duplication followed by sequence divergence. In addition, a *Schizanthus* gene grouped sister to the *euFULII* clade (77% pairwise identity with *Schizanthus MBP20*). This may also be a divergent genus-specific paralog, but since *Schizanthus* is one of the earliest diverging genera (Olmstead et al., 2008; Särkinen et al., 2013), it is also possible this gene might be a remaining paralog from the reported whole genome duplication/triplication that occurred early in Solanaceae diversification (Blanc and Wolfe, 2004; Schlueter et al., 2004; Song et al., 2012; The Tomato Genome Consortium, 2012; Albert and Chang, 2014; Bombarely et al., 2016). Examination of sequences showed that these are not likely to be splice isoforms. We also found potential evidence of loss – not every Solanaceae species we studied had a copy of each *euFUL* gene. We did not, for example, find *FUL2* genes in *Iochroma, Fabiana, Solandra, Juanulloa, Schizanthus*, or *Goetzia*, even though these all had genes in the *FUL1* clade (see Table S1 in Supplementary Data Sheet S1 for a complete list). However, although this may represent paralog loss, it is possible we did not recover all gene copies due to PCR primer mismatches, low expression levels, or the absence of transcript in the sampled tissue.

In addition to the major shift to fleshy fruit in the Solanoideae subfamily, fleshy fruits have independently evolved in *Cestrum* and *Duboisia*, and there has also been a reversal to a dry fruit in *Datura* (Knapp, 2002). Our analysis does not include genes from *Duboisia*, but the *euFUL* genes from *Cestrum* and *Datura* grouped in positions in the tree that were expected based on their phylogenetic position, and did not show any notable differences in sequence from the *euFUL* genes of their close relatives.

### *euFULI* and *euFULII* Clade Duplicates Have Experienced Different Levels of Purifying Selection

We compared dN/dS ratios between and among Solanaceae *euFULI* and *euFULII* lineages, as well as between sequences before and after the transition to fleshy fruit, to investigate if any changes in selection might be correlated with sequence diversification. All ω values from our analyses are closer to 0 than to 1 (Table 1 and Table S3 in Supplementary Data Sheet S1), which indicates that all *euFUL* gene clades are under strong purifying selection (Yang, 1997; Yang and Nielsen, 2000; Torgerson et al., 2002; Almeida and Desalle, 2009). Studies suggest that this is the norm for most protein coding genes, and that under such stringent evolutionary constraints, slight differences in evolutionary rates may result in functional diversification (Yu et al., 2017). Our data show a weakening of purifying selection in *FUL1* genes relative to *FUL2* genes (ω = 0.17 vs. 0.11, *p* < 0.0005) and in *MBP10* genes relative to *MBP20* genes (ω = 0.19 vs. 0.15, *p* < 0.01). Immediately after the *euFULI* duplication, the *FUL1* and *FUL2* lineage genes would have been fully redundant, which might have allowed the reduction in purifying selection on the *FUL1* genes resulting in potential functional divergence. Similarly, the duplication that resulted in the two *euFULII* gene clades would have resulted in redundancy in the *MBP10* and *MBP20* lineages, possibly allowing the more rapid diversification of *MBP10* genes.

Although studies indicate that the *euFULI* genes of tomato have novel functions compared to those in dry fruit (Gu et al., 1998; Smykal et al., 2007; Bemer et al., 2012; Shima et al., 2014; Wang et al., 2014), it remains unclear whether the new functions are the result of changes in coding sequences, regulatory regions, or downstream gene targets. Our analysis shows that *euFUL* genes in both dry- and fleshy-fruited species are evolving at similar rates (Table S3 in Supplementary Data Sheet S1). This suggests conservation of the coding sequences in both fleshy- and dry-fruited species despite the central roles in the development of these distinct fruit morphologies.

Sixty-four of the sequences in our analysis were from fleshy-fruited species whereas only 42 were from dry-fruited species. Although, we had broad representation across the dry grade, it is possible with additional representation from dry fruited species, more evolutionary patterns would be revealed (Anisimova et al., 2001; Domazet-Loso, 2003; Nielsen et al., 2005).

### FUL1 and MBP10 Proteins Show Amino Acid Changes in Conserved Functional Domains

An analysis of selection across an entire sequence may indicate different types of selection for the whole gene, but this overlooks the fact that key residues may be undergoing rapid evolution that may result in functional changes (Ota and Nei, 1994; Nei et al., 1997; Yang and Bielawski, 2000; Piontkivska et al., 2002; Martinez-Castilla and Alvarez-Buylla, 2003; Jeffares et al., 2006). Other empirical studies have further described functional changes due to a change in a single amino acid residue (Ingram, 1957; Hanzawa et al., 2005; Hichri et al., 2011; Zhao et al., 2012; Fourquin et al., 2013; Dai et al., 2016; Sakuma et al., 2017) specifically associated with changes in polarity (Schröfelbauer et al., 2004; Hoekstra et al., 2006) or conformation (Aseev et al., 2012). Studies in *A. thaliana*, show that a single amino acid mutation in GLABRA1 (GL1) results in the inhibition of trichome formation (Dai et al., 2016) and a change of a single residue is sufficient to convert the function of TERMINAL FLOWER 1 (TFL1), which inhibits flower formation, to that of the closely related FLOWERING LOCUS T (FT), which promotes flowering (Hanzawa et al., 2005). Three-dimensional modeling has also shown that a single amino acid change in a highly conserved domains may lead to changes in protein–protein interactions (Teng et al., 2009; David et al., 2012; Li et al., 2014). We searched for individual sites in the predicted amino acid sequences that showed evidence of positive selection within the gene groups that, although under purifying selection, were found to have statistically significantly accelerated evolutionary rates (i.e., the *FUL1* and *MBP10* clades) to determine if any amino acid changes at these sites had the potential to result in a change in protein function.

Our findings show that more residues are rapidly changing in the K domain compared to the M and I domains (Figure S4 in Supplementary Data Sheet S1). The K domain is predicted to have an α-helix structure that facilitates protein–protein interactions (Figure S5 in Supplementary Data Sheet S1) (Yang et al., 2003a,b; Kaufmann et al., 2005; Immink et al., 2010). The α-helix structure depends on conserved hydrophobic residues spaced through the domain (Eisenberg et al., 1982). Therefore, changes to protein residues that alter charge and/or conformation in this region can lead to changes in such interactions. Most of the rapidly evolving sites did not show an amino acid change specifically associated with the shift to fleshy fruit, but rather showed changes and reversals over the course of gene evolution. Interestingly, in the FUL1 proteins, we found one site in the K domain, corresponding to the 153rd residue in the tomato protein (Slugina et al., 2018), at which 11 out of 15 sequences from dry-fruited species have a negatively charged glutamate (E) residue. In comparison, 100% of the fleshy clade contains a non-polar residue: valine (V) (13 species) or methionine (1 species). However, since the remaining four FUL1 sequences from dry-fruited species have non-polar glutamine (Q) or V at this site, the change from charged to non-polar is not associated with the shift to fleshy fruit. In addition, a PROVEAN analysis predicted the changes at this site to be neutral with regards to function.

Two other sites in the FUL1 K domain show changes that are predicted to have functionally deleterious consequences according to our PROVEAN analysis (Choi, 2012; Choi et al., 2012; Choi and Chan, 2015). These include a charged histidine (H) to a non-polar glutamine/asparagine (Q/N) transition at the 95th residue and a charged lysine (K) to non-polar glutamine/threonine (Q/T) transition at the 157th residue (Figure S5 in Supplementary Data Sheet S1). Polar residues are important for protein–protein interactions of the K domain α-helix (Sheinerman et al., 2000; Curran and Engelman, 2003; Ma et al., 2003; Zhou et al., 2018) and changes might disrupt interactions with other proteins (Liu et al., 2014). However, since these changes are not correlated with the fruit type, it seems unlikely that any alteration to protein function affects fruit morphology. It is also plausible that any negative effect at these sites is masked by the FUL2 paralog, which is likely to be functionally redundant (Bemer et al., 2012; Wang et al., 2014). This is consistent with FUL1 evolving relatively faster (Table 1 and Table S3 in Supplementary Data Sheet S1), thus enabling divergence compared to FUL2, which appears to be more highly functionally conserved based on stricter sequence conservation.

None of the sites undergoing positive change in the K domain of MBP10 showed a change in charge, suggesting these changes are not likely to affect protein function. We also observed residues in the M domain that are under diversifying selection in both the FUL1 and MBP10 clades. These residues are located not in the α-helix region that directly binds to DNA, but in the β-sheet region of the MADS domain (Figure S4 in Supplementary Data Sheet S1) (Immink et al., 2010). β-sheets are important for protein arrangement in three dimensional space. Therefore, any changes in this region might change protein conformation, influencing DNA binding of the α-helix as well as the ability of the euFUL proteins to form higher order complexes (Pellegrini et al., 1995). However, these shifts were reversible, with no phylogenetic pattern or change in charge, and there was no correlation with the fruit type. Therefore it is unlikely that these shifts have significant functional impact.

A previous report that investigated the evolution of MADS-box genes in *A. thaliana* also found rapidly evolving sites in the M and K domains of Type II MADS-box proteins, which might have been involved in the functional diversification of this group, but did not report changes in the I domain (Martinez-Castilla and Alvarez-Buylla, 2003). Residues in this domain that are directly involved in forming an α-helix structure are expected to be highly conserved, whereas the remaining residues may not be under such constraints (Yang et al., 2003a,b; Kaufmann et al., 2005). We found residues in the conserved region of the I domain that are undergoing diversifying selection in both FUL1 and MBP10 clades. Of these, one site in FUL1 and three sites in MBP10 had undergone changes in charge but none were predicted to negatively affect the function (Figures S4, S5 in Supplementary Data Sheet S1). In addition, as with the sites in the M and K domains, none of these was correlated with the Solanaceae phylogeny or changes in fruit morphology. It has been reported that higher rates of substitution in lineages that show weakened purifying selection or even diversifying selection may be occurring at residues of minimal functional importance (Jacobsen et al., 2016). This might explain the apparent ease of reversibility and lack of phylogenetic signal among the rapidly changing sites we observed.

### The *MBP10* and *MBP20* Clades Are the Result of a Tandem Duplication Event

The *FUL1* and *FUL2* genes of tomato are located on different chromosomes (6 and 3, respectively), which is consistent with the proposed Solanaceae whole genome duplication (Blanc and Wolfe, 2004; Schlueter et al., 2004; Song et al., 2012) or triplication (The Tomato Genome Consortium, 2012; Albert and Chang, 2014; Vanneste et al., 2014; Bombarely et al., 2016) followed by loss of one paralog. The lack of a *FUL2* ortholog in our dataset from *Goetzia* or *Schizanthus* (Figure 2), the two earliest diverging genera that we included in our analyses, raises the possibility that the *FUL1/FUL2* clades originated via a duplication that occurred after the diversification of these genera, and not as a result of a whole genome event that preceded the diversification of the family. Whole genome sequences from multiple early diverging lineages will be needed to determine the timing and nature of these early events.

We did not recover an *MBP10* ortholog from any of the genera that diverged prior to *Brunfelsia* (Figure 2 and Figure S2 in Supplementary Data Sheet S1). Our investigation revealed that in tomato, both *MBP10* and *MBP20* are located on chromosome 2, about 14.3 million base pairs apart. The 1 million base-pair region surrounding each gene shows synteny, but the order of the homologous regions is reversed (Figure 3). Together, this suggests that the *MBP10/MBP20* clades are the result of a tandem duplication accompanied by an inversion (Purugganan et al., 1995; Vision et al., 2000; Achaz et al., 2001; Prince and Pickett, 2002). Supporting this, a previous report that investigated genomic duplication events in tomato also found evidence for large-scale intra-chromosomal duplications in chromosome 2 (Song et al., 2012). Although the authors suggest this event was concurrent with a whole genome duplication at the origin of the family, they give a large window, 36–82 million years ago (MYA), for the timing of this event. The stem age of the family is predicted to be approximately 49 MYA (Särkinen et al., 2013), indicating that this duplication might have happened later in Solanaceae diversification. Our data suggest that this duplication event is independent of the reported whole genome events, occurring prior to the diversification of the *Brunfelsia* clade but after the event that produced the *FUL1* and *FUL2* clades (Figure 2 and Figure S2 in Supplementary Data Sheet S1).

The expected topology for the *euFULII* clade, based on a duplication prior to the divergence of the *Brunfelsia* clade, would be a paraphyletic grade of pre-duplication *euFULII* genes, from species that diversified prior to *Brunfelsia*, and nested *MBP10* and *MBP20* clades that would include post-duplication genes from all species that diversified subsequent to the duplication. However, in our tree, the pre-duplication genes do not form such a basal grade (Figure 2). Rather, they form a clade with the post-duplication *MBP20* genes. The results of our PAML analyses indicate that the *MBP20*-clade genes show less sequence divergence than *MBP10* genes; this higher degree of similarity among pre-duplication sequences and post-duplication *MBP20* genes may underlie their grouping into one clade (Pegueroles et al., 2013).

Our results indicate that the *euFULII* duplication occurred prior to the origin of the clade containing *Brunfelsia*. We would therefore expect to find both an *MBP10* and an *MBP20* in all species of that clade. However, we did not find an *MBP10* ortholog in members of this clade other than *Brunfelsia*. *MBP10* appears to have been lost from the genome of *Petunia*, based on analyses of multiple fully sequenced genomes (Bombarely et al., 2016), and potentially from *Plowmania* and *Fabiana.* We were able to recover *MBP10* orthologs from *Nicotiana* and most other later-diverging genera. However, our analysis includes fewer species from the dry grade of the Solanaceae phylogeny than the fleshy-fruited Solanoideae clade (17 out of 45) and even fewer species that diverged prior to *Brunfelsia* (7). In the *MBP10* clade in particular, our analysis includes 13 orthologs from species in the fleshy-fruited clade but just four from the dry-fruited species, and our analysis only includes sequence data from four genera that diverged prior to the origin of the Brunfelsia clade (*Streptosolen*, *Cestrum*, *Goetzia*, *Schizanthus*) (Figure 1, 2). Thus there may be genera that originated prior to *Brunfelsia* that contain *MBP10* that our sampling did not include. Floral and fruit transcriptomes, which provided *MBP10* orthologs from later diverging species, yielded no *MBP10* sequences from *Cestrum* and *Schizanthus*; nonetheless, whole genome sequences of early diverging species are needed to determine the timing of the *MBP10*/*MBP20* duplication.

### *euFULII* Expression Divergence May Be Associated With *Cis*-Regulatory Re-coupling

Our analysis of Solanaceae *euFUL* homologs show that *FUL1* and *FUL2* are broadly expressed in leaves, flowers, and fruit (Figure 4 and Figure S1 in Supplementary Data Sheet S1). This overall similarity in expression may indicate a conservation of *cis*-regulatory elements in gene copies following duplication (Haberer et al., 2004). Supporting this, our investigation into the number of putative TF binding sites in the promoter region of *euFULI* homologs did not reveal statistically significant differences (Table S4 in Supplementary Data Sheet S1). In tomato fruit development, *FUL1* expression increases with time, whereas *FUL2* expression reaches a maximum at early stages and then decreases over later stages (Figure 4 and Figure S1 in Supplementary Data Sheet S1). This variation in expression associated with the developmental stages might be due to changes in *cis*-elements as a result of the accumulation of random mutations over time (Force et al., 1999; Haberer et al., 2004).

Our analysis did find differences in the number and location of predicted binding sites for specific TFs or families, for instance for ARF, STK, and EIN3 TFs, which may account for the types of differences in expression seen between *euFUL* paralogs. The 5 kb region upstream of the *FUL1* transcription start site in tomato contains three putative ARF binding sites but the corresponding region of *FUL2* in tomato contains no such motifs (Table S4 in Supplementary Data Sheet S1). ARF TFs, important in tomato fruit development, are activated in response to auxin and may upregulate or repress downstream genes (de Jong et al., 2010, 2015; Liu et al., 2018); the absence of binding sites from the *FUL2* promoter is the type of factor that might underlie differences in expression observed between *FUL1* and *FUL2*. Predicted STK binding sites are only found in the promoters of potato *FUL1*, tomato *FUL2* and woodland tobacco *MBP10.* STK and STK-like proteins appear to function in storage protein synthesis, glucose reception, and vegetative and reproductive development (Zourelidou et al., 2002; Curaba et al., 2003; Bömer et al., 2011; Chung et al., 2016; Nietzsche et al., 2017). Meanwhile, the 2 kb upstream region of *FUL2* contains a putative site for EIN3. This protein is involved in the development of tomato in response to ripening-associated ethylene production (Tieman et al., 2001). No such motifs are found in the corresponding region of *FUL1.* In contrast, the 2–5 kb region in *FUL2* contains four putative sites for EIN3 while the corresponding region in *FUL1* contains three such sites (Table S4 in Supplementary Data Sheet S1). Such variation in number and location of TF binding sites has been shown to be associated with the temporal differences in gene expression (Lebrecht et al., 2005; Liu et al., 2006; Giorgetti et al., 2010; Guertin and Lis, 2010; White et al., 2013; Ezer et al., 2014; Levo et al., 2015; Payne and Wagner, 2015).

Whereas the *euFULI* members largely overlap in spatial expression with some variation associated with developmental stages, the *euFULII* homologs show less consistent spatial expression patterns. Only *MBP20* is expressed in tomato roots and potato fruit while only *MBP10* is expressed in potato tubers (Figure 4). However, these “on” or “off” expression patterns cannot be explained by the presence or absence of any putative TF binding sites (Table S4 in Supplementary Data Sheet S1). These two paralogs, which appear to be the result of a tandem duplication and inversion, are located approximately 14.3 Mbp apart (Figure 3) on chromosome 2. Although gene clusters resulting from tandem duplications are often coexpressed, this is not the case when there are large physical distances between the genes (Lercher et al., 2003). An investigation into the expression of human transgenes in mice also found changes in expression as a consequence of an inversion, possibly through disrupting enhancer activity or changes to chromatin structure (Tanimoto et al., 1999; Vogel et al., 2009; Puig et al., 2015). Chromosomal rearrangements such as inversions may also result in novel connections between coding regions and other promoters or long distance regulatory motifs while disrupting the original regulatory mechanisms (Kmita et al., 2000; Lupiáñez et al., 2015). This sort of re-coupling of one of the two paralogs might lead to the types of contrasting expression patterns observed for *MBP10* and *MBP20*. However, the expression patterns are not consistent across species (Figure 4 and Figure S1 in Supplementary Data Sheet S1) and this might be due to additional changes following the inversion (Cosner et al., 1997; Lupski, 1998; Haberle et al., 2008; Chiang et al., 2012). An in-depth analysis of the entire loci and their genomic environment for all paralogs in multiple species would be necessary to determine if the tandem duplication and inversion are associated with changes in proximity to heterochromatin, additional rearrangements, or other phenomena that might have altered gene expression.

### *MBP10* Shows Signs of Pseudogenization

The first intron of some MADS-box genes contains *cis*-elements important for the regulation of expression (Gazzani et al., 2003; Michaels et al., 2003; Schauer et al., 2009). Studies have found that deletions in the first intron of a *FUL-like* gene in *Aegilops tauschii* alters expression and results in the loss of the vernalization requirement (Fu et al., 2005; Takumi et al., 2011). Consistent with this, the first introns of angiosperm *euFUL* orthologs are generally in the range of 1–10 kb (Table 2) (Takumi et al., 2011). In contrast, tomato *MBP10* has a short first intron of 80 bp. We compared the putative TF binding sites in the first introns of *MBP10* and *MBP20* in tomato to characterize potential loss of such sites, which might suggest reduced gene regulation. The first intron of *MBP10* is predicted to have no TF binding sites, while the first intron of *MBP20* is predicted to contain 88 TF binding sites (Figure S3 in Supplementary Data Sheet S1). These included binding sites for MYB, HSF, Dof, WRKY, and MADS-box TFs. Specific TFs predicted to bind to these sites include MYB2 and C1 (MYB), which play roles in anthocyanin accumulation and lignin biosynthesis, PBF (Dof), which plays a role in endosperm storage protein accumulation, and SPF1 (WRKY), thought to function in fruit ripening (Bovy et al., 2002; Fei et al., 2004; Hwang et al., 2004; Lee et al., 2010; Xu et al., 2014; Jun et al., 2015). A similar pattern was found in analysis of the first intron of *MBP10* in *Nicotiana obtusifolia*, which is 110 bp (Table 2). This analysis found three putative TF binding sites for MYB2 and one for PBF. By contrast, the first intron of *N. obtusifolia MBP20* is predicted to have 133 TF binding sites and include a repertoire similar to those found for tomato *MBP20*. To determine whether the difference in TF binding site number between the paralogs represented a gain of sites in the *MBP20* genes or a loss in the *MBP10* genes, we also searched for TF binding sites in the first intron of *AGL79*, the single *euFULII* ortholog in *A. thaliana* (Gao et al., 2018). We found that it contains 49 predicted TF binding sites for five different TFs in four families: MYB (MYB2, GAMYB), HSF (HSF1), WRKY (SPF1), and GT-box (GT-1). Although this number is substantially smaller than the number of sites predicted in the first introns of the Solanaceae *MBP20* genes, the results suggest that there has been a loss of TF binding sites in *MBP10*.

**Table 2:** Approximate lengths of the first introns of several *FUL* homologs.

Gene	Length (bp)
*Solanum lycopersicum FUL1*	5,000
*S. lycopersicum FUL2*	4,400
*S. lycopersicum MBP10*	80
*S. lycopersicum MBP20*	2,500
*S. pimpinellifolium MBP10*	80
*Nicotiana obtusifolia FUL1*	5,300
*N. obtusifolia FUL2*	3,800
*N. obtusifolia MBP10*	110
*N. obtusifolia MBP20*	3,000
*Arabidopsis thaliana FUL*	900
*A. thaliana AGL79*	1,700
*Aegilops tauschii VRN-D1* (Takumi et al., 2011)	8,600

Core-eudicot *euFUL* and basal-eudicot *FUL-like* genes frequently have broad expression patterns and are generally expressed in fruit (Ferrándiz et al., 2000; Shchennikova et al., 2004; Kim et al., 2005; Hileman et al., 2006; Bemer et al., 2012; Pabón-Mora et al., 2012, 2013; Scorza et al., 2017). Therefore, the absence or extremely weak expression of *MBP10* in fruits of all species, and its weak expression in most organs of tomato and potato is notable (Figure 4 and Figure S1 in Supplementary Data Sheet S1). This relatively weak expression may at least in part be due to the loss of TF binding sites in the first intron and suggests a potentially reduced role in regulating fruit-related developmental processes. Importantly, the loss of putative TF binding sites and low expression, combined with the faster evolutionary rate, suggest *MBP10* might be in the process of becoming a pseudogene. Further support for this hypothesis comes from an examination of the *MBP10* sequences, which suggests that at least two of the sequences in our study (from *N. sylvestris* and *Dunalia spinosa*) show a frameshift that would result in an premature stop codon.

## Conclusion

Our results suggest that there was a weakening in purifying selection following the *euFUL* gene duplications in Solanaceae, resulting in coding sequence diversification in *FUL1* and *MBP10* clades relative to *FUL2* and *MBP20*. Expression of the *euFULI* genes is broad, while the *euFULII* genes have contrasting patterns at the organ level, potentially resulting from *cis*-regulatory changes associated with the inversion event. We also found evidence to suggest that the *MBP10* clade is becoming a pseudogene. Although at least some clades of Solanaceae *euFUL* genes took on new functions associated with the development of fleshy fruit we did not find any amino acid shifts that were correlated with the change in fruit type. It is also possible that the novel functions are a consequence of downstream changes, perhaps as the result of changes in binding partners or targets. Therefore, the mechanism underlying the shift in *euFUL* function from dry to fleshy fruit in Solanaceae awaits additional analyses.

## Author Contributions

AL designed and supervised research, and assisted in writing the paper. DM contributed to the design of the study, generated *Cestrum diurnum, C. nocturnum*, and *Schizanthus grahamii* transcriptome libraries, retrieved sequences from PCR-based methods and database mining, analyzed the data, and wrote the paper. CE assisted with PAML analysis, contributed suggestions for analyses, and made suggestions on the paper. AR generated *Dunalia spinosa, Fabiana viscosa, Grabowskia glauca*, and *Salpiglossis sinuata* transcriptome libraries, contributed suggestions for analyses, contributed in recording the associated protocols, and commented on the paper. JM retrieved sequences from PCR-based methods. MS generated the *Nicotiana obtusifolia* transcriptome libraries and additional sequences using PCR-based methods. NP-M generated *Brunfelsia australis* and *Streptosolen jamesonii* transcriptome libraries, contributed suggestions for analyses, and made suggestions on this paper.

## Conflict of Interest Statement

The authors declare that the research was conducted in the absence of any commercial or financial relationships that could be construed as a potential conflict of interest.

## Footnotes

1 ftp://ftp.solgenomics.net/manuscripts/Litt_2018

2 https://www.ncbi.nlm.nih.gov/blast

3 https://db.cngb.org/blast4onekp/

4 https://sites.google.com/a/ualberta.ca/onekp

5 http://abacus.gene.ucl.ac.uk/software/paml.html

6 http://datamonkey.org/meme

7 http://provean.jcvi.org

8 http://www.sbg.bio.ic.ac.uk/~phyre2

9 https://www.geneious.com

10 http://alggen.lsi.upc.es/recerca/frame-recerca.html

11 http://bar.utoronto.ca

12 http://benthgenome.qut.edu.au/

13 http://plantpan2.itps.ncku.edu.tw

14 http://datamonkey.org/meme
