# Supplementary material for: Evolution and Diversification of FRUITFULL Genes in Solanaceae
Source: Front Plant Sci. 2019 Feb 21;10:43. doi: 10.3389/fpls.2019.00043 (PMC6394111; doi:10.3389/fpls.2019.00043)
Supplement: Supplementary file 1 [file Data_Sheet_1.pdf]

Supplementary Material

Evolution and Diversification of *FRUITFULL* Genes in Solanaceae

Dinusha C. Maheepala, Christopher A. Emerling, Alex Rajewski, Jenna Macon, Maya Strahl, Natalia Pabón-Mora, Amy Litt*

*** Correspondence:** Amy Litt: amy.litt@ucr.edu

List of figures and tables:

Figure S1: *euFUL* expression in *S. lycopersicum* and *S. pimpinellifolium*.

Figure S2: The presence/absence of *MBP10/MBP20*.

Figure S3: Putative transcription factor binding sites for tomato *MBP20* first intron.

Figure S4: Individual sites in euFUL proteins are undergoing rapid evolution. Number of branches under positive selection in (A) FUL1, and (B) MBP10 proteins.

Figure S5: Rapidly evolving amino acid residues with a change in charge in FUL1 and MBP10 plotted on the predicted structures of the relevant ortholog in *Solanum lycopersicum.*

Table S1: Sources and accession numbers of sequence data.

Table S2: Sampled tissue and repository for data generated in this study.

Table S3: Evolutionary rates of *euFUL* gene clades.

Table S4: Putative transcription factor (TF) binding sites in the 2/5kb promoter regions of tomato (*Sl*), potato (*St*) and woodland tobacco (*Ns*).

Table S5. Primers sequences used for PCR and cloning in this study.

Figure S1: *euFUL* expression in *S. lycopersicum* and *S. pimpinellifolium*.
(A) A composite of gel images of RT-PCR for *FUL1* (35 cycles), *FUL2* (30 cycles), *MBP10* (30 cycles), *MBP20* (35 cycles) and *ACTIN* (28 cycles) in *Solanum pimpinellifolium*. The same cDNA was used for all five amplifications of a given tissue. (B) Transcript numbers of *euFUL* genes converted to log counts per million (LogCPM) from RNAseq libraries (unpublished data) of *S. lycopersicum* var. Ailsa Craig (AC) and *S. pimpinellifolium* (PIMP) fruit. We compiled libraries from five different stages of fruit development (Gillaspy, 1993;Tanksley, 2004) in each of the two species. Pre-anth.: 1 day pre-anthesis; DPA: days post-anthesis.

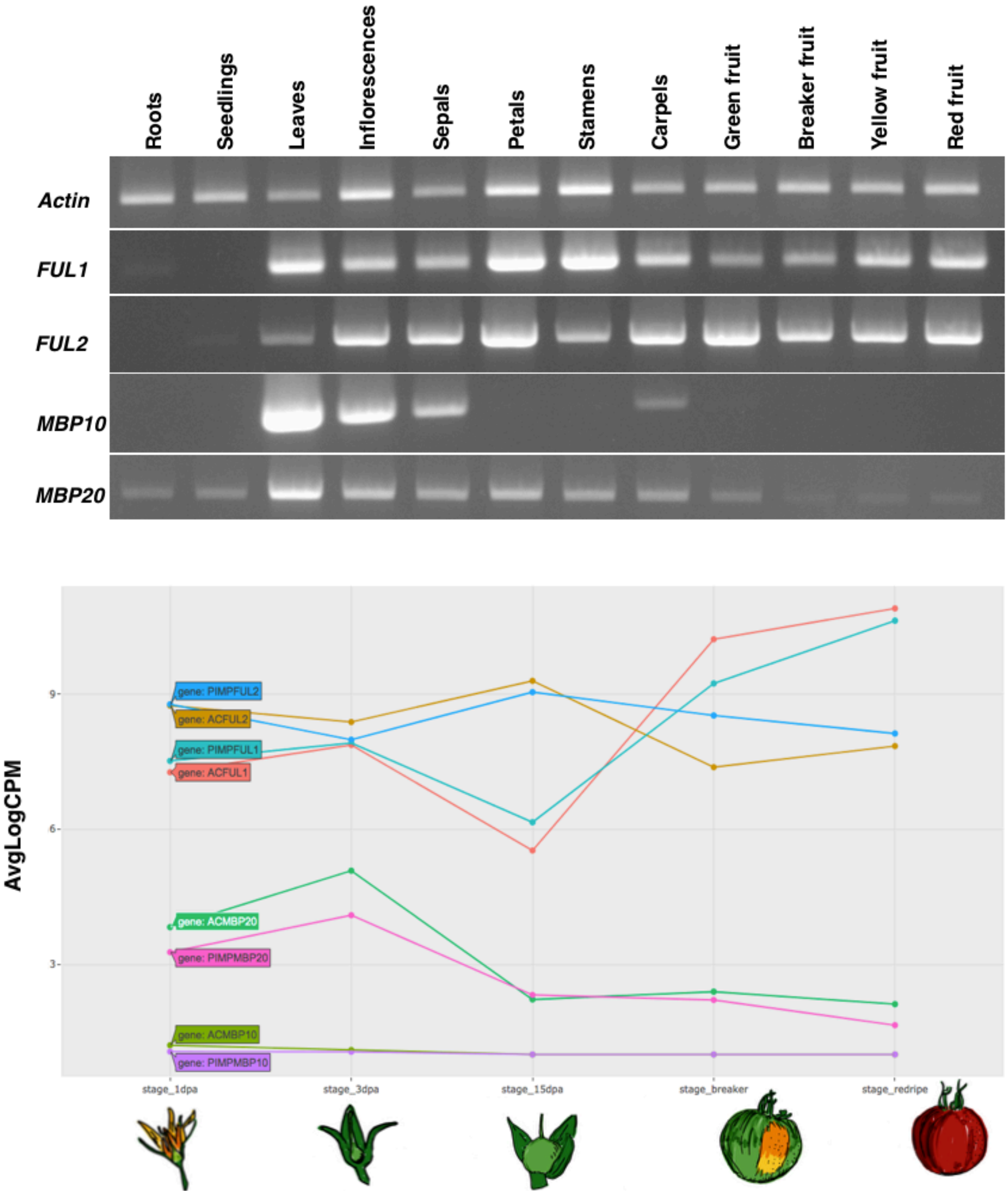

Figure S2: The presence/absence of *MBP10/MBP20*. The star indicates where in the phylogeny we have evidence for a tandem gene duplication related to the origin of the *MBP10/MBP20* clades. The cyan squares and the red dots represent the taxa that we have included in our analysis and those in which we have have found *MBP10*, respectively.

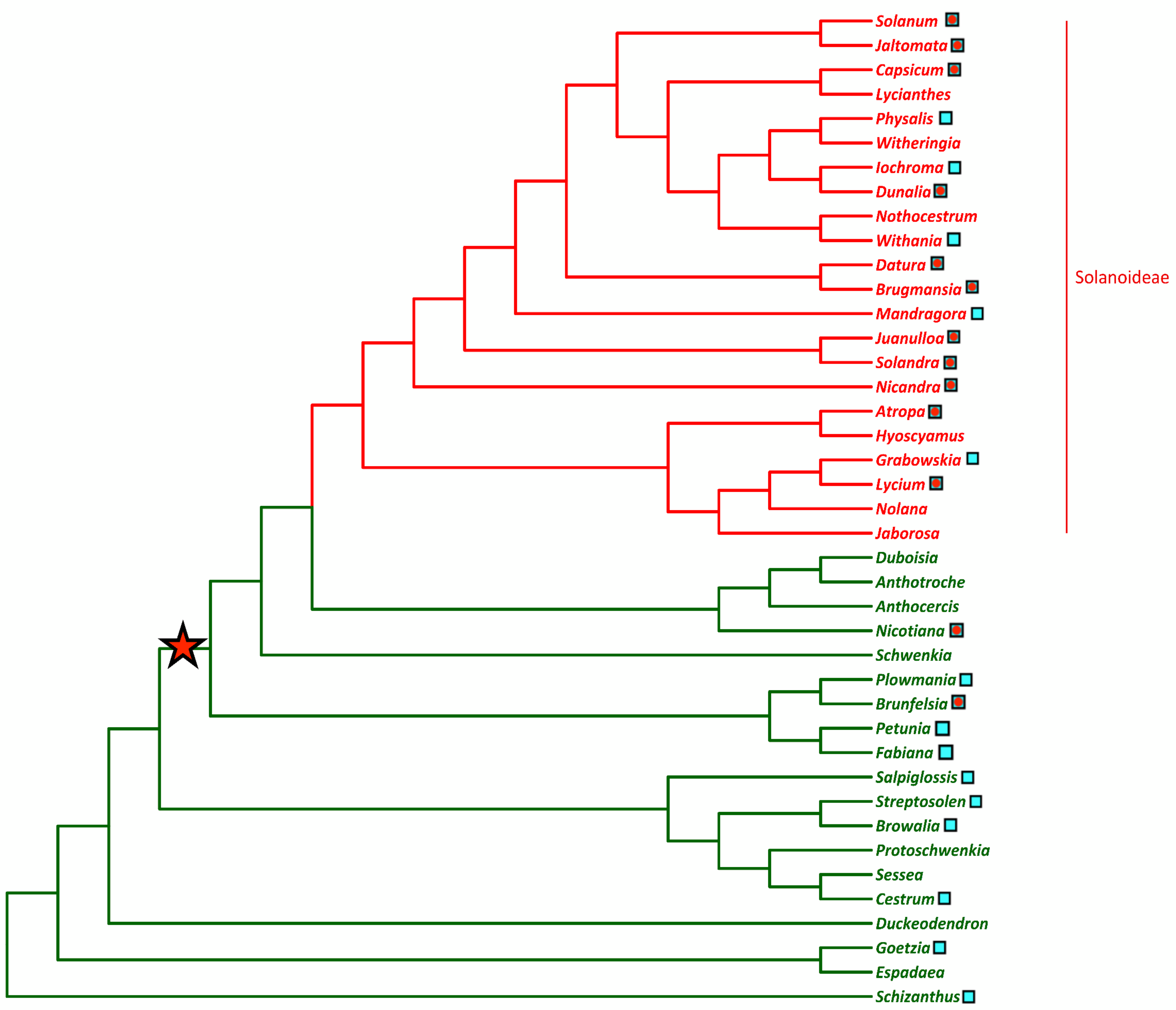

Figure S3: Putative transcription factor binding sites for tomato *MBP20* first intron.

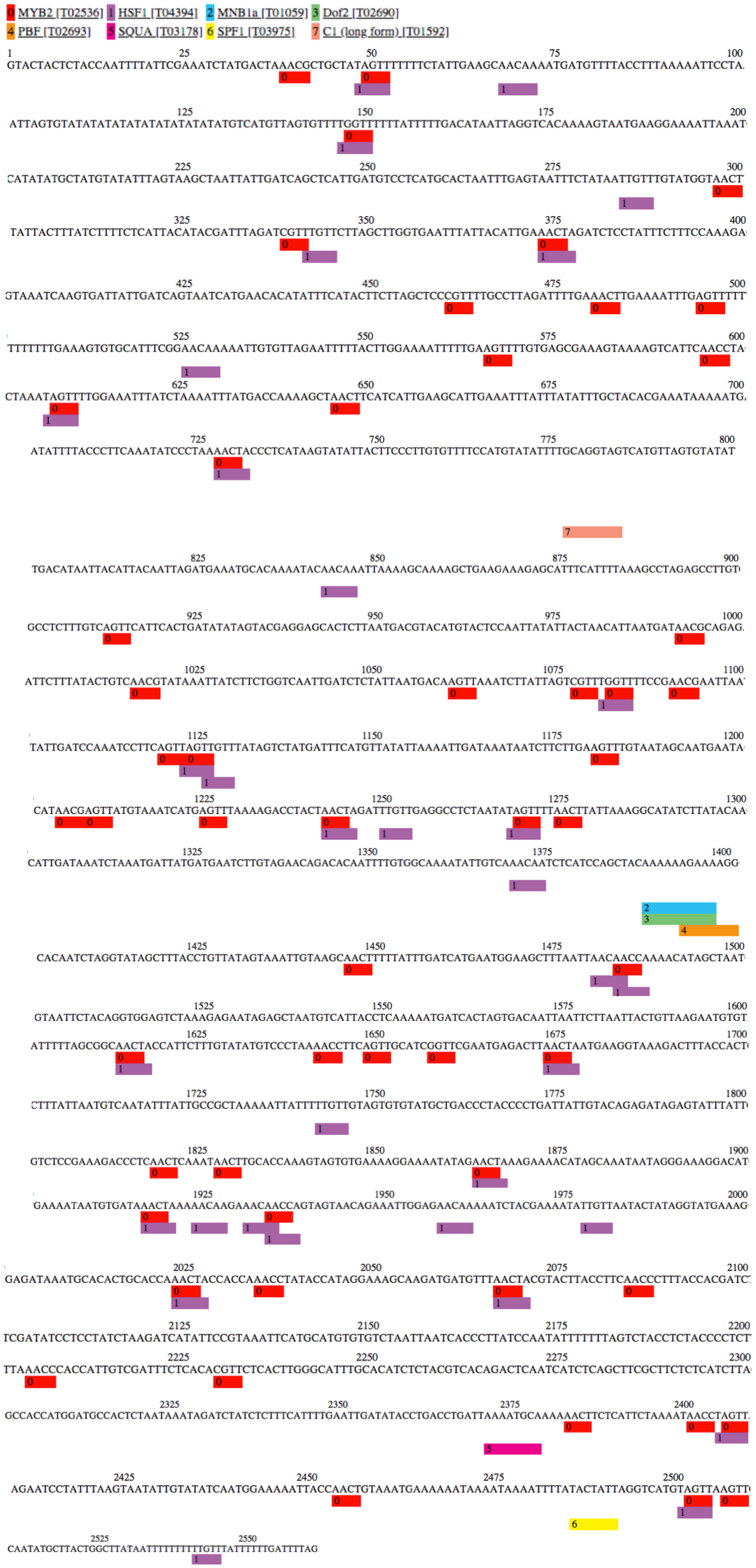

Figure S4: Individual sites in euFUL proteins are undergoing rapid evolution. Number of branches under positive selection in (A) FUL1, and (B) MBP10 proteins.

(A)

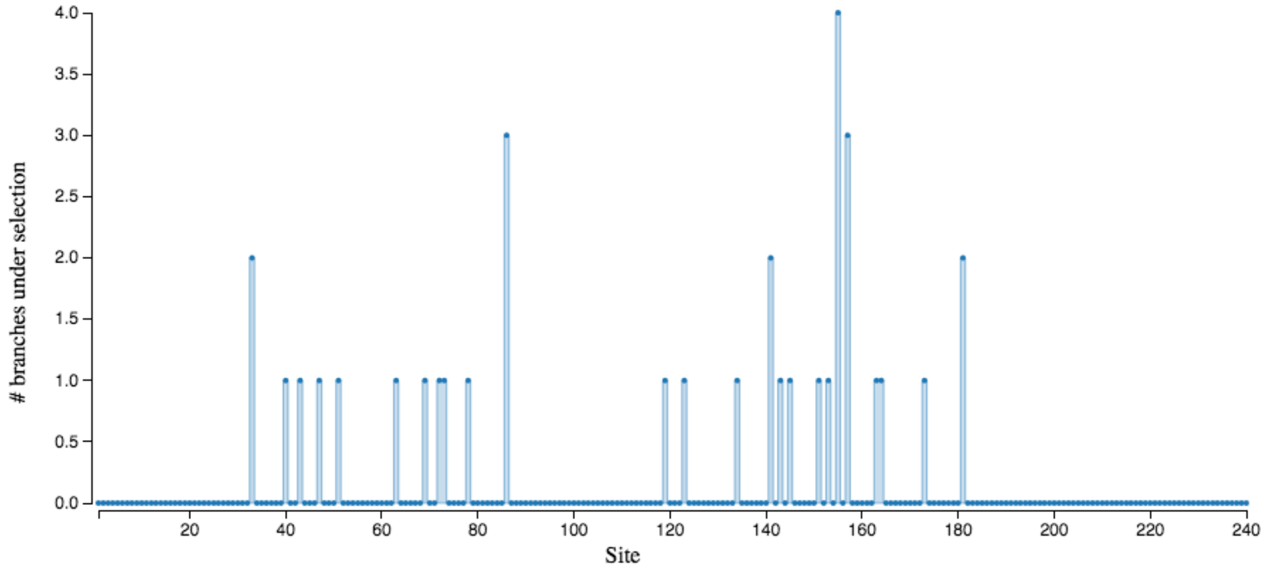

(B)

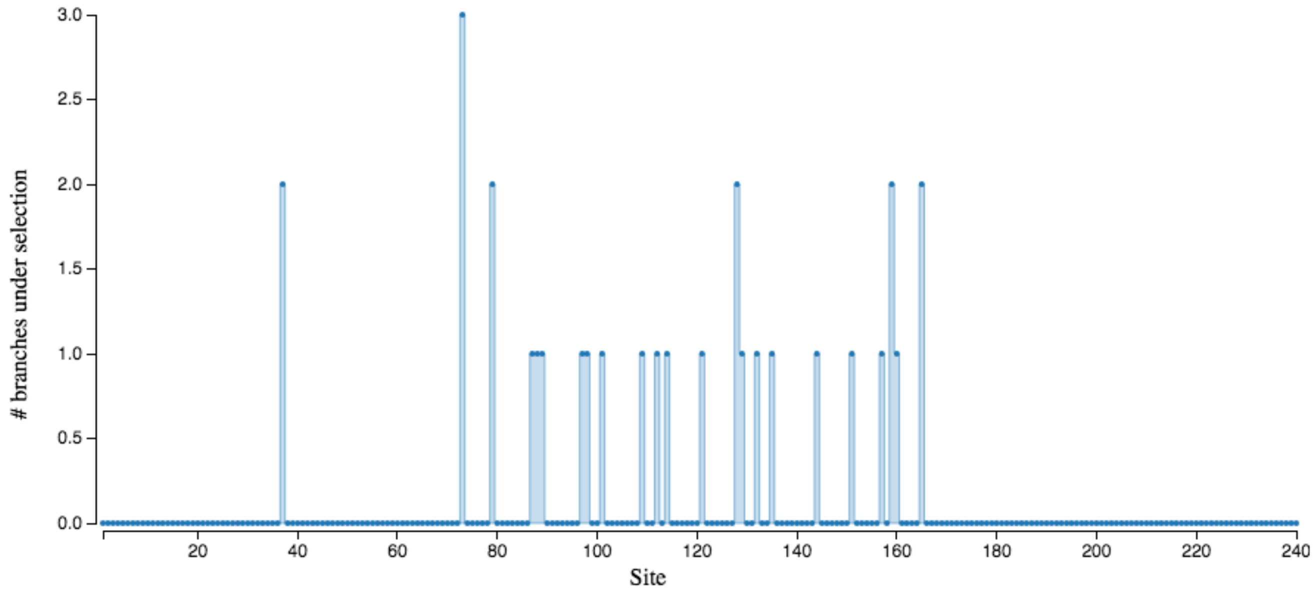

Figure S5: Rapidly evolving sites that show a change in charge in FUL1 (A) MADS, (B) I, and (C) K domains and MBP10 (D) MADS, (E) I, and (F) K domains plotted on the predicted structures of the relevant ortholog in tomato. Green helix: α-helix; blue arrow: β– sheet; red ellipse: sites with potentially deleterious changes in function.

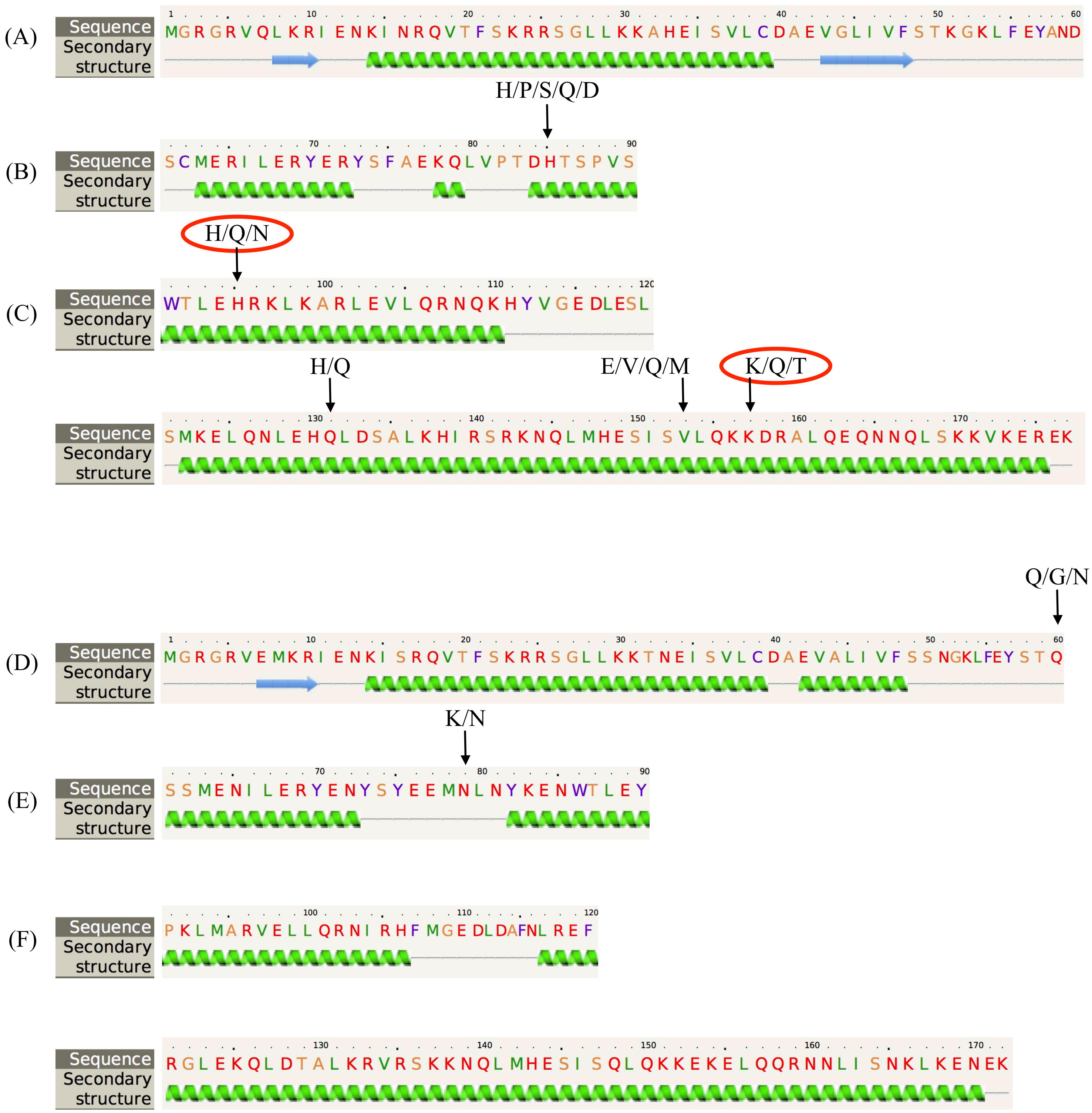

Table S1. Sources of euFUL sequence data. NA = sequences obtained from online sources; NYBG = The New York Botanical Garden; UCR = University of California, Riverside; UdeA = University of Antioquia, Colombia.

Species	Gene clade	Source	Source of seed/ tissue
Solanaceae			
Atropa belladonna	*FUL2*	oneKP (BOLZ_scaffold_2025221)	NA
Atropa belladonna	*MBP10*	oneKP (BOLZ_scaffold_2003062)	NA
Atropa belladonna	*MBP20*	oneKP (BOLZ_scaffold_2003065)	NA
Browallia americana	*MBP20*	Degenerate primer PCR (NYBG; GenBank MH931101)	NYBG
Brugmansia suaveolens	*FUL2*	Degenerate primer PCR (NYBG; GenBank MH931102)	NYBG
Brugmansia suaveolens	*MBP10*	Degenerate primer PCR (UCR; GenBank MH931103)	NYBG
Brugmansia suaveolens	*MBP20*	Degenerate primer PCR (NYBG; GenBank MH931104)	NYBG
Brunfelsia australis	*FUL1*	Transcriptomes (UdeA; GenBank MH931105)	UdeA
Brunfelsia australis	*FUL2*	Transcriptomes (UdeA; GenBank MH931106)	UdeA
Brunfelsia australis	*MBP10*	Transcriptomes (UdeA; GenBank MH931107)	UdeA
Brunfelsia australis	*MBP20*	Transcriptomes (UdeA; GenBank MH931108)	UdeA
Capsicum annuum	*FUL2*	GenBank (NM_001324623.1)	NA
Cestrum aurantiacum	*FUL1*	Degenerate primer PCR (NYBG; GenBank MH931109)	NYBG
Cestrum diurnum	*FUL1*	Transcriptomes (UCR; GenBank MH931110)	Chileflora.com
Cestrum diurnum	*FUL2*	Transcriptomes (UCR; GenBank MH931111)	Chileflora.com
Cestrum diurnum	*MBP20*	Transcriptomes (UCR; GenBank MH931112)	Chileflora.com
Cestrum nocturnum	*FUL1*	Transcriptomes (UCR; GenBank MH931113)	Chileflora.com
Cestrum nocturnum	*MBP20*	Transcriptomes (UCR; GenBank MH931114)	Chileflora.com
Datura inoxia	*FUL1*	Degenerate primer PCR (UCR; GenBank MH931117)	NYBG
Datura inoxia	*FUL2*	Degenerate primer PCR (NYBG; GenBank MH931115)	NYBG
Datura inoxia	*MBP20*	Degenerate primer PCR (NYBG; GenBank MH931116)	NYBG
Datura metel	*FUL2*	oneKP (NVS_scaffold_2038250)	NA
Datura metel	*MBP10*	oneKP (JNVS_scaffold_2043321)	NA
Datura metel	*MBP20*	oneKP (JNVS_scaffold_2040412)	NA
Dunalia spinosa	*FUL1*	Transcriptomes (UCR; GenBank MH931118)	Chileflora.com
Dunalia spinosa	*FUL2*	Transcriptomes (UCR; GenBank MH931119)	Chileflora.com
Dunalia spinosa	*MBP10*	Transcriptomes (UCR; GenBank MH931120)	Chileflora.com
Dunalia spinosa	*MBP20*	Transcriptomes (UCR; GenBank MH931121)	Chileflora.com
Fabiana viscosa	*FUL1*	Transcriptomes (UCR; GenBank MH931122)	Chileflora.com
Fabiana viscosa	*MBP20*	Transcriptomes (UCR; GenBank MH931123)	Chileflora.com
Goetzia sp.	*FUL1*	Degenerate primer PCR (NYBG; GenBank MH931124)	Fairchild Tropical Garden
Goetzia sp.	*MBP20*	Degenerate primer PCR (NYBG; GenBank MH931125)	Fairchild Tropical Garden
Grabowskia glauca	*FUL1*	Transcriptomes (UCR; GenBank MH931126)	Chileflora.com
Grabowskia glauca	*FUL2*	Transcriptomes (UCR; GenBank MH931127)	Chileflora.com
Grabowskia glauca	*MBP20*	Transcriptomes (UCR; GenBank MH931128)	Chileflora.com
Iochroma fuchsioides	*FUL1*	Degenerate primer PCR (NYBG; GenBank MH931129)	NYBG
Iochroma fuchsioides	*MBP20*	Degenerate primer PCR (NYBG; GenBank MH931130)	NYBG
Jaltomata procumbens	*MBP10*	Degenerate primer PCR (NYBG; GenBank MH931131)	Chileflora.com
Jaltomata procumbens	*MBP20*	Degenerate primer PCR (NYBG; GenBank MH931132)	Chileflora.com
Juanalloa mexicana	*FUL1*	Degenerate primer PCR (NYBG; GenBank MH931133)	Chileflora.com
Juanalloa mexicana	*MBP10*	Degenerate primer PCR (NYBG; GenBank MH931134)	Chileflora.com
Juanulloa mexicana	*MBP20*	Degenerate primer PCR (UCR; GenBank MH931135)	Chileflora.com
Lycium barbarum	*FUL2*	oneKP (LWCK_scaffold_2017804)	NA
Lycium barbarum	*MBP10*	oneKP (LWCK_scaffold_2003151)	NA
Lycium sp.	*FUL2*	oneKP (OSMU_scaffold_2017828)	NA
Lycium sp.	*MBP20*	oneKP (OSMU_scaffold_2017069)	NA
Mandragora officianarum	*MBP20*	Degenerate primer PCR (UCR; GenBank MH931136)	alchemy-works.com
Nicandra physalodes	*FUL1*	Degenerate primer PCR (UCR; GenBank MH931137)	NYBG
Nicandra physalodes	*FUL2*	Degenerate primer PCR (NYBG; GenBank MH931138)	NYBG
Nicandra physalodes	*MBP10*	Degenerate primer PCR (NYBG; GenBank MH931140)	NYBG
Nicandra physaloides	*MBP20*	Degenerate primer PCR (UCR; GenBank MH931139)	NYBG
Nicotiana langsdorffii x Nicotiana sanderae	*FUL1*	GenBank (DQ471787.1)	NA

Nicotiana obtusifolia	*FUL1*	Transcriptomes (NYBG; GenBank MH931141)	US Nicotiana Germplasm Collection
Nicotiana obtusifolia	*FUL2*	Transcriptomes (NYBG; GenBank MH931142)	US Nicotiana Germplasm Collection
Nicotiana obtusifolia	*MBP10*	Transcriptomes (NYBG; GenBank MH931143)	US Nicotiana Germplasm Collection
Nicotiana obtusifolia	*MBP20*	Transcriptomes (NYBG; GenBank MH931100)	US Nicotiana Germplasm Collection
Nicotiana sylvestris	*FUL2*	GenBank (NM_001302579.1)	NA
Nicotiana sylvestris	*MBP10*	GenBank (XM_009763875.1)	NA
Nicotiana sylvestris	*MBP20*	GenBank (XM_009776014.1)	NA
Nicotiana tabacum	*FUL1*	GenBank (DQ534202.1)	NA
Nicotiana tabacum	*FUL2*	GenBank (NM_001325205.1)	NA
Nicotiana tomentosiformis	*FUL2*	GenBank (XM_009627559.2)	NA
Nicotiana tomentosiformis	*MBP10*	GenBank (XM_009618497.2)	NA
Petunia exserta	*FUL1*	Degenerate primer PCR (NYBG; GenBank MH931144)	NYBG
Petunia exserta	*FUL2*	Degenerate primer PCR (UCR; GenBank MH931145)	NYBG
Petunia exserta	*MBP20*	Degenerate primer PCR (NYBG; GenBank MH931146)	NYBG
Petunia hybrida	*FUL1*	GenBank (AF176782.1)	NA
Petunia hybrida	*FUL2*	GenBank (AF176783.1)	NA
Petunia hybrida	*MBP20*	GenBank (AF335245.1)	NA
Physalis pubescens	*FUL2*	Degenerate primer PCR (UCR; GenBank MH931147)	NYBG
Plowmania nyctaginoides	*FUL2*	Degenerate primer PCR (NYBG; GenBank MH931149)	NYBG
Plowmania nyctaginoides	*MBP20*	Degenerate primer PCR (NYBG; GenBank MH931150)	NYBG
Salpiglossis sinuata	*FUL1*	Transcriptomes (UCR; GenBank MH931151)	Chileflora.com
Salpiglossis sinuata	*FUL2*	Transcriptomes (UCR; GenBank MH931152)	Chileflora.com
Salpiglossis sinuata	*MBP20*	Transcriptomes (UCR; GenBank MH931153)	Chileflora.com
Schizanthus grahamii	*FUL1*	Transcriptomes (UCR; GenBank MH931154)	Chileflora.com
Schizanthus grahamii	*MBP*	Transcriptomes (UCR; GenBank MH931155)	Chileflora.com
Schizanthus grahamii	*MBP20*	Transcriptomes (UCR; GenBank MH931156)	Chileflora.com
Solandra maxima	*FUL1*	Degenerate primer PCR (NYBG; GenBank MH931157)	NYBG
Solandra maxima	*MBP10*	Degenerate primer PCR (UCR; GenBank MH931158)	NYBG
Solandra maxima	*MBP20*	Degenerate primer PCR (UCR; GenBank MH931159)	NYBG
Solanum betaceum	*FUL1*	Degenerate primer PCR (NYBG; GenBank MH931160)	NYBG
Solanum cheesmanii	*FUL1*	oneKP (UGJI_scaffold_2125762)	NA
Solanum commersonii	*FUL1*	GenBank (AF002666.1)	NA
Solanum dulcamara	*FUL1*	oneKP (GHLP_scaffold_2055028)	NA
Solanum dulcamara	*FUL2*	oneKP (GHLP_scaffold_2043858)	NA
Solanum lycopersicum	*FUL1*	GenBank (X60757.1, NC_015443.2)	NA
Solanum lycopersicum	*FUL2*	GenBank (AK327202.1, NC_015440.2)	NA
Solanum lycopersicum	*MBP10*	GenBank (XM_004233345.3, NC_015439.2)	NA
Solanum lycopersicum	*MBP20*	GenBank (XM_010317904.2, NC_015439.2)	NA
Solanum pimpinellifolium	*FUL1*	SolGenomics (Sopim06g069430.0.1, contig:unspecified:1090932:1:2183:1)	NA
Solanum pimpinellifolium	*FUL2*	SolGenomics (Sopim03g114830.0.1, contig:unspecified:5836421:1:4775:1)	NA
Solanum pimpinellifolium	*MBP10*	SolGenomics (Sopim02g065730.0.1, contig:unspecified:6626854:1:18349:1)	NA
Solanum pimpinellifolium	*MBP20*	SolGenomics (Sopim02g089210.0.1, contig:unspecified:1205759:1:362:1)	NA
Solanum ptychanthum	*FUL2*	oneKP (DLJZ_scaffold_2010261)	NA
Solanum ptychanthum	*MBP10*	oneKP (DLJZ_scaffold_2053583)	NA
Solanum quitoense	*FUL1*	Degenerate primer PCR (NYBG; GenBank MH931161)	NYBG
Solanum quitoense	*MBP20*	Degenerate primer PCR (NYBG; GenBank MH931162)	NYBG
Solanum sisymbriifolium	*FUL2*	Degenerate primer PCR (UCR; GenBank MH931163)	NYBG
Solanum tuberosum	*FUL1*	GenBank (NM_001288213.1)	NA
Solanum tuberosum	*FUL2*	GenBank (XM_006345039.2)	NA
Solanum tuberosum	*MBP10*	GenBank (XM_006365593.2)	NA
Solanum xanthocarpum	*FUL2*	oneKP (LQJY_scaffold_2015692)	NA
Streptosolen jamesonii	*FUL*	Transcriptomes (UdeA; GenBank MH931164)	Parque Arvi, Vereda Santa Elena, El Tambo, Colombia
Streptosolen jamesonii	*FUL1*	Transcriptomes (UdeA; GenBank MH931165)	Parque Arvi, Vereda Santa Elena, El Tambo, Colombia
Streptosolen jamesonii	*FUL2*	Transcriptomes (UdeA; GenBank MH931166)	Parque Arvi, Vereda Santa Elena, El Tambo, Colombia

Streptosolen jamesonii	*MBP20*	Transcriptomes (UdeA; GenBank MH931167)	Parque Arvi, Vereda Santa Elena, El Tambo, Colombia
Withania somnifera	*FUL2*	Degenerate primer PCR (UCR; GenBank MH931169)	alchemy-works.com
Withania somnifera	*MBP20*	Degenerate primer PCR (NYBG; GenBank MH931168)	alchemy-works.com
Arabidopsis thaliana			
Arabidopsis thaliana	*FUL*	GenBank (NM_125484.4)	NA
Arabidopsis thaliana	*AGL79*	GenBank (NM_113925.3, NC_003074.8)	NA
Convolvulaceae			
Convolvulus arvensis	*FUL*	oneKP (CPOC_scaffold_2010291)	NA
Cuscuta pentagonia	*FUL*	oneKP (AHRN_scaffold_2082598)	NA
Ipomoea coccinea	*FUL*	oneKP (ERWT_scaffold_2042911)	NA
Ipomoea hederacea	*FUL*	oneKP (QSLH_scaffold_2053329)	NA
Ipomoea indica	*FUL*	oneKP (OQBM_scaffold_2015411)	NA
Ipomoea lindheimeri	*FUL*	oneKP (NAUM_scaffold_2053058)	NA
Ipomoea nil	*FUL*	oneKP (NHAG_scaffold_2046547)	NA
Ipomoea pubescens	*FUL*	oneKP (EMBR_scaffold_2056425)	NA
Ipomoea purpurea	*FUL*	oneKP (VXKB_scaffold_2010684)	NA
Ipomoea quamoclit	*FUL*	oneKP (ALUC_scaffold_2003652)	NA

Table S2. Sampled tissue and repository for data generated in this study.

Species	Data repository	Sampled tissue
Browallia americana	GenBank	Leaves
Brugmansia suaveolens	GenBank	Leaves
Brunfelsia australis	GenBank	Vegetative and reproductive meristems, floral buds, leaves or fruits
Cestrum aurantiacum	GenBank	Leaves
Cestrum diurnum	GenBank, SolGenomics	Fruits, leaves
Cestrum nocturnum	GenBank, SolGenomics	Inflorescences
Datura inoxia	GenBank	Leaves
Dunalia spinosa	GenBank, SolGenomics	Leaves
Fabiana viscosa	GenBank, SolGenomics	Leaves
Goetzia sp.	GenBank	Leaves
Grabowskia glauca	GenBank, SolGenomics	Leaves
Iochroma fuchsioides	GenBank	Leaves
Jaltomata procumbens	GenBank	Leaves
Juanalloa mexicana	GenBank	Leaves
Mandragora officianarum	GenBank	Leaves
Nicandra physalodes	GenBank	Leaves
Nicotiana obtusifolia	GenBank, SolGenomics	Leaves
Petunia exserta	GenBank	Leaves, flowers
Physalis pubescens	GenBank	Leaves
Plowmania nyctaginoides	GenBank	Leaves
Salpiglossis sinuata	GenBank	Leaves
Schizanthus grahamii	GenBank, SolGenomics	Inflorescences, leaves
Solandra maxima	GenBank	Leaves
Solanum betaceum	GenBank	Leaves
Solanum quitoense	GenBank	Leaves
Solanum sisymbriifolium	GenBank	Leaves
Streptosolen jamesonii	GenBank	Vegetative and reproductive meristems, floral buds, leaves or fruits
Withania somnifera	GenBank	Leaves

Table S3: Evolutionary rates of *euFUL* gene clades. Comparisons in which gene clades are evolving at statistically different rates ($p < 0.05$) are highlighted in red.

Comparison	Model	ω0	ω1	ω2	2ΔL	df	P-value
euFULI vs *euFULII*	M0 (ω_0: all branches)	0.1423	_	_	4.4050	2	0.1105
	M2 (ω_0: *euFULI*; ω_1: *euFULII*)	0.1311	0.1577	_			
FUL1 vs *FUL2*	$M2_A$ (ω_0: background; ω_1: *FUL1* and *FUL2*)	0.1577	0.1311	_	15.5040	1	0.0001
	$M2_B$ (ω_0: background; ω_1: *FUL1*; ω_2: *FUL2*)	0.1577	0.1710	0.1064			
MBP10 vs *MBP20*	$M2_A$ (ω_0: background; ω_1: *MBP10* and *MBP20*)	0.1311	0.1577	_	7.0291	1	0.0080
	$M2_B$ (ω_0: background; ω_1: *MBP10*; ω_2: *MBP20*)	0.1279	0.1939	0.1514			
FUL1 vs other *euFUL*	M0 (ω_0: all branches)	0.1423	_	_	5.3906	1	0.0001
	M2 (ω_0: *FUL1*; ω_1: other *euFUL*)	0.1706	0.1344	_			
FUL2 vs other *euFUL*	M0 (ω_0: all branches)	0.1423	_	_	19.3663	1	0.0000
	M2 (ω_0: *FUL2*; ω_1: other *euFUL*)	0.1065	0.1622	_			
MBP10 vs other *euFUL*	M0 (ω_0: all branches)	0.1423	_	_	8.7258	1	0.0031
	M2 (ω_0: *MBP10*; ω_1: other *euFUL*)	0.1943	0.1348	_			
MBP20 vs other *euFUL*	M0 (ω_0: all branches)	0.1423	_	_	0.7890	1	0.3743
	M2 (ω_0: *MBP20*; ω_1: other *euFUL*)	0.1519	0.1390	_			
All dry vs all fleshy	M0 (ω_0: all branches)	0.1423	_	_	3.1106	1	0.0777
	M2 (ω_0: all dry; ω_1: all fleshy)	0.1309	0.1530	_			
*FUL1*dry vs *FUL1*fleshy	$M2_A$ (ω_0: background; ω_1: all *FUL1*)	0.1344	0.1706	_	0.0011	1	0.9735
	$M2_B$ (ω_0: background; ω_1: *FUL1*dry; ω_2: *FUL1*fleshy)	0.1344	0.1712	0.1701			
*FUL2*dry vs *FUL2*fleshy	$M2_A$ (ω_0: background; ω_1: all *FUL2*)	0.1622	0.1065	_	0.1109	1	0.5283
	$M2_B$ (ω_0: background; ω_1: *FUL2*dry; ω_2: *FUL2*fleshy)	0.1622	0.0999	0.1109			
*MBP10*dry vs *MBP10*fleshy	$M2_A$ (ω_0: background; ω_1: all *MBP10*)	0.1348	0.1943	_	0.2540	1	0.6142
	$M2_B$ (ω_0: background; ω_1: *MBP10*dry; ω_2: *MBP10*fleshy)	0.1348	0.1753	0.2009			
*MBP20*dry vs *MBP20*fleshy	$M2_A$ (ω_0: background; ω_1: all *MBP20*)	0.1390	0.1518	_	2.9476	1	0.0860
	$M2_B$ (ω_0: background; ω_1: MBP20dry; ω_2: *MBP20*fleshy)	0.1391	0.1318	0.1766			

Table S4. Putative transcription factor (TF) binding sites in the 2/5kb promoter regions of tomato (Sl), potato (St) and woodland tobacco (Ns). Cells highlighted in red had zero predicted transcription factor binding sites.

TF family	# Putative binding sites in the promoter (2/5kb upstream of ATG)																							
	SlFUL1		SlFUL2		SlMBP10		SlMBP20		StFUL1		StFUL2		StMBP10		StMBP20		NsFUL1		NSFUL2		NsMBP10		NsMBP20	
	2kb	5kb	2kb	5kb	2kb	5kb	2kb	5kb	2kb	5kb	2kb	5kb	2kb	5kb	2kb	5kb	2kb	5kb	2kb	5kb	2kb	3.3kb	2kb	5kb
MYB	41	72	30	48	46	76	31	51	45	78	47	62	38	79	31	56	50	71	40	63	52	60	43	82
NAC	9	13	3	19	5	7	8	9	5	8	5	13	4	8	8	15	6	18	15	17	2	2	16	19
Dof	8	15	11	13	15	16	14	14	9	15	13	16	11	16	9	13	12	15	5	10	13	15	12	14
GATA	2	17	11	17	13	14	7	7	5	17	12	14	2	13	3	9	2	9	12	13	12	13	5	12
MADS	5	12	7	11	3	4	6	11	4	5	5	11	11	11	4	18	6	12	2	10	3	7	1	4
AP2	14	28	6	22	14	20	4	9	12	22	5	21	16	17	21	30	14	27	25	44	5	19	27	38
EIN3		3	1	5	2	5	4	4	2	2	4	5	4	5	3	6	3	3		1	4	4	3	4
WOX	4	5	5	5	5	5	5	5	5	5	5	5	2	2	5	5	4	5	3	4	2	3	5	5
ARF	2	3			1	1	1	2	2	3	2	2	2	3	2	2	2	3	3	4	1	1	1	3
bHLH	4	6	3	9	6	7	7	8	6	9	3	40	39	39	36	36	6	8	8	13	3	5	40	40
WRKY	2	25	10	24	10	16	10	25	2	26	27	29	28	29	28	28	11	19	22	35	9	10	29	31
SBP	21	21	20	21	16	16	15	15	21	21	19	20	3	5	14	14	18	19	20	21	4	15	3	13
HD-ZIP	27	32	24	28	28	31	32	32	31	32	29	2	25	28	29	32	23	30	19	26	20	28	23	27
bZIP	19	20	7	15	25	29	24	27	19	33	10	40	52	53	48	51	9	27	12	41	7	10	30	31
Trihelix	5	5	3	4	2	5	4	6	5	5	2	5	2	6	4	6	5	5	5	5	2	2	4	5
YABBY	2	4	2	5	3	4	2	4	2	4	3	4		4	3	4	4	4	1	3	3	4	1	2
HSF		1	1	1	1	1	1	1	1	1	3	3	1	1		1	1	3	1	1	1	1		2
TCP	7	19	2	5	19	20	2	9	6	20		12	8	8	7	8	2	5	4	4	3	9	6	8
TBP	8	8	8	8	7	8	8	8	8	8	8	8	6	8	8	8	8	8	7	7	7	8	6	8
C2H2	7	10	6	8	7	7	7	9	6	9	4	8	7	12	5	8	3	7	2	7	7	8	7	11
LBD				1						1				1					1	1				2
BES1	1	1			1	1				1		2	2	2	2	2							2	2
TALE	1	3	1	1	3	4	2	2	1	4	1	1	4	4	3	3	1	4	3	4	1	1	5	5
CAMTA				2			3	3					3	3	3	3		3	3	3	3	3		1
SRS	1	2	1	1	2	2	2	2	2	2	2	2		3	1	2	1	1	2	3	2	2	2	3
NF-Y/ CBF	1	1	1	2	1	2	1	2	1	1	1	1	2	2	1	1	1	1	1	2	1	1	1	1
CSD	1	1	1	1	1	1	1	1	1	1	1	1	1	1	1	1	1	1			1	1	1	1
DHN	1	1	1	1	1	1	1	1	1	1	1	1	1	1	1	1	1	1	1	1	1	1	1	1
STK			1	1						1												2		
FAR1				1			2	2					2	2	2	2						1		1
GRAS	1	1					1	1	1	1	1	1			1	1	1	1	1	1				
TCX/ CPP									4	5	5	5	3	5	5	5	4	5	1	2	5	5	1	3
GRF	1	1								1		1					1	1	1	1	1	1	1	1
Totals	195	330	166	279	237	303	205	270	207	342	218	335	279	371	288	371	200	316	220	347	175	242	276	380

Table S5. Primers sequences used for PCR and cloning in this study.

Primer Name	Target	Sequence
AN221 (An221-Actin_121_Fwd)	Actin	GATGGATCCTCCAATCCAGACACTGTA
AN222 (An222-Actin_122_Rev)	Actin	GTATTGTGTTGGACTCTGGTGATGGTGT
AN104 (Litt_Fwd_MADS_3)	MADS-Box	GTNCARYTNARRMGNATNGARAAYAAGAT
AN105 (Oligo_dT_1228)	MADS-Box	GGCCAGTGAATTGTAATACGACTCACTATAGGGAGGCGGTTTTTTTTTTTTTTTTTTTTTT
AN106F (Litt_Fwd_nested_AP1MDS1)	MADS-Box	GCICWTGARMTNTCNRTNYTNTGYGATGC
AN108 (Litt_Rev_nested_AGL8R)	MADS-Box	AGRTGRYKAASCATCCAIKGIGGCA
UC1_MBP20R_1	MBP20	BTHNTTGCTCCAAATGGTCC
UC2_MBP20R_2	MBP20	BTHNTTCCTCCAAAAGSCCC
UC3_MBP10R_1	MBP10	GKTTGCTKCTTCTCATTTYCTT
UC4_FUL1R_1	FUL1	TGTTGAAAAATAAATGAAGGTGA
UC5_FUL2R_1	FUL2	GGSGGCATCACAGAAGYGTT
UC6_FUL2R_2	FUL2	CATGGCGGCATYACAGTGTT
UC19_FUL2R_3	FUL2	KAASYRTYYRKKGNGGCATBACAG
UC20_FUL2R_4	FUL2	KAASCATCCAKGGNGGCATBACAG
UC21_FUL1R_2	FUL1	CAYCCAKKGKGGCATYRMWVTATTA
UC22_FUL1R_3	FUL1	CATCCAKKGKGGCATCACAGTATTA
UC23_MBP10R_2	MBP10	WRTTARMACYADKRCGAKTTTGMCC
UC24_MBP10R_3	MBP10	WATTAGAACCADGRCGAKTTTGMCC
UC25_MBP20R_3	MBP20	CCAAWHKTHARRTYWKRAVBHYRNH
UC26_MBP20R_4	MBP20	CCAATTGTTAGGTTAGGAAGTTGGT
UC27_M13F	TOPO vector	GTA AAA CGA CGG CCA G
UC28_M13R	TOPO vector	CAG GAA ACA GCT ATG AC

References

Gillaspy, G., Ben-David, H., and Gruissem, W. (1993). Fruits: A Developmental Perspective. Plant Cell 5, 1439.

Tanksley, S. D. (2004). The genetic, developmental, and molecular bases of fruit size and shape variation in tomato. Plant Cell 16 Suppl, S181–9.
