# Supplementary material for: Evolution and Diversification of FRUITFULL Genes in Solanaceae
Source: Front Plant Sci. 2019 Feb 21;10:43. doi: 10.3389/fpls.2019.00043 (PMC6394111; doi:10.3389/fpls.2019.00043)
Supplement: Supplementary file 2 [file Data_Sheet_2.docx]

Supplementary File 2

Solanaceae euFUL gene alignment

StreptosolenFUL1 ---------------------------------------------------------------------------------------------------GAGATCTCTGTGCTTTGTGATGCTGAGGTTGGTTTGATTGTTTTTTCCACTAAAGGCAAACTCTTTGAGTTT---TCCTCTGATTCCTGCATGGAAAAGATCCTTGAAAGATATGAAAGATACTCATTTGCTGAGCGGCAGCTGGTTAATACTGATCATAGCTCC---CCGGGAGGCTGGACTCTGGAACATGCAAAGCTTAAGGCCAGAATTGAGGTTCTGCAGAGAAACGAAAGGCACTATATGGGAGAAGAATTGGATTCGTTGAGTATGAAGGAACTTCAGAATGTGGAGCACCAGCTTGATTCTGCTCTTAAACACATTCGATCAAGAAAGAACCAACTGATGCACCAGTCCATTTCTGAGCTTCAGAAGAAGGACAAAGCATTGCAGGAGCAAAATAAACAGCTTTCGAAGAAGGTGAAGGAAAGGGAGAAAGAGGTG---------------------------------------------------------------------------------------------------------------------------------------------------------------------------------

Schizanthus_grahamiiFUL1 ---------------------------------------------------------------------------------------------------------------------GATGCTGAGGTTGGTTTGATTGTTTTCTCAACTAAGGGAAAACTCTACGAGTAT---GCCACCGATTCCTGCATGGAAAGGATTCTCGAAAGGCACGAACGATACTCGTATGCTGAGAGGCACCTTGTGGCTACTGATCATAGCTCC---ACGGGAAGCTGGACTCTGGAACATGCCAAACTTAAGGCCAGAGTTGAGGTTTTGCAGAGAAACCAAAGGCATTACATGGGAGAAGACTTGGACACGCTAAGTCTGAAAGAGCTTCAGAATCTAGAGCACCAGCTGGATTCTGCTCTTAAACACATTCGGTCAAGAAAGAACCAACTGATGCATGAATCCATATCTGAGCTGCAAAAAAAGGACAAAGCATTGCAGGAGCAAAACAACCAGCTTTCTAAGAAGGTTAAGGAGAGGGAGAAAGAGCAG---------------------------------------------------------------------------------------------------------------------------------------------------------------------------------

Petunia_exsertaFUL1 ---------------------------------------------------------------------------------------------GCGCTTGAAATTTCNGTGTTGTGTGATGCTGAAGTTGGTTTAACTGTTTTTTCTACTAAAGGCAAACTCTTTGAGTAT---GCTACTGATTCTTGCATGGAGAGGATTCTTGAAAGATATGAAAGATACTCATATGCTGAGAGGCAGCTTGTTTCTACTGATCATAGCTCC---CCGGGAAGCTGGAATCTGGAACATGCAAAACTTAAGGCCAGAATTGAGGTTTTGCAGAGAAACCAAAGGCATTATATGGGAGAAGATTTGGACTCGTTAAGTATGAAAGAACTTCAGAATTTGGAACAACAACTAGATTCTTCTCTTAAACACATTCGATCAAGAAAGAACCAATTGATGCATGAGTCCATTTCTGAGCTTCAAAAAAAGGACAAATCATTGCAAGAGCAAAACAACCTTCTTTCAAAGAAGGTGAAGGAGAGGGAGAAAGAGTTG---------------------------------------------------------------------------------------------------------------------------------------------------------------------------------

Petunia_hybridaFUL1 ATGGGAAGAGGAAGAGTGCAGATGAAGAGAATTGAGAATAAAATTAATAGACAAGTTACTTTTTCAAAACGTCGATCTGGATTATTGAAGAAAGCTCATGAAATCTCTGTGCTTTGTGATGCTGAAGTTGGTTTAATTGTTTTTTCTACTAAAGGCAAACTCTTTGAGTAT---GCTACTGATTCTTGCATGGAGAGGATTCTTGAAAGATATGAAAGATACTCATATGCTGAGAGGCAGCTTGTTTCTACTGATCATAGCTCC---CCGGGAAGCTGGAATCTGGAACATGCAAAACTTAAGGCCAGAATTGAGGTTGTGCAGAGAAACCAAAGGCATTATATGGGAGAAGATTTGGACTCGTTAAGTATGAAAGACCTTCAGAATTTAGAACAACAGCTGGATTCTTCTCTTAAACACATTCGATCAAGAAAGAACCAATTGATGCATGAGTCCATTTCTGAGCTTCAAAAAAAGGACAAATCATTGCAAGAGCAAAACAACCTTCTTTCAAAGAAGGTGAAGGAGAGGGAGAAAGAGTTG---------------------------------------------------------------------------------------------------------------------------------------------------------------------------------

Solandra_maximaFUL1 --------------------------------------------------------------------------------------------TGCGCATGAGATGTCAATCTTTTGTGATGCTGAGGTTGGTTTGATTGTTTTTTCTACTAAAGGCAAACTCTTTGAATAT---GCCACTGATTCTTGCATGGAAAGGATACTTGAAAGATATGAAAGATACTCATTTGCTGAGAGGCTGCTTGTTCCTCCTGATCATAGCTCC---CCGGGAAGCTGGACTCTGGAACAGGCAAAACTTAAGGCCAGACTTGAGGTTCTGCAGAGGAACCAAAAGCATTATGTGGGAGAAGATTTGGACTCGTTAAATATGAAAGAACTTCAGAAACTGGAGCAACAGCTTGATTCTGCTCTTAAACACATTCGATCAAGAAAGAACCAATTGATGCATGAGTCCATTTCTATGCTTCAAAAAAAGGACAAAGCATTGCAGGAGCAAAACCACCAGCTTTCCAAGAAGGTGAAGGAGAGGGAGAAAGAGCTG---------------------------------------------------------------------------------------------------------------------------------------------------------------------------------

Goetzia_sp.FUL1 ---------------------------------------------------------------------------------------------GCGCTTGAACTTTCGGTGTTCTGCGATGCTGATGTTGGTTTAACCGTTTTCTCTACTAAAGGCAAACTCTACGAGTAT---GCCTCTGACTCTTGCATGGAAAAGATTGTTGAAAGGTACGAAAGATATTCATATGCTGGGAGAGAGCTTGTTGCGACTGATAGTAGCTCA---CCGCGGAACTGGACTCTGGGACATGCCAAGCTTAAGGCAAGACTTGAGGTTTTGCAGAGAAACCAAAGGCATTATATGGGAGAAGACTTGAACTCTTTAAGCATGAAAGACCTTCAGAACTTAGAGCACCAGCTCGATTCTGCTCTTAAACACATTCGATCAAGAGAGAACCAATTGATGCATGAGTGTATATCTCAACTGCAGAAAAAGGGCAAAGCATTGCAGGAGCAAAACAACCAGCTATCAAAGAAGGCGAAG---AAGGAGAAAGAGCCG---------------------------------------------------------------------------------------------------------------------------------------------------------------------------------

FabianaFUL1 ATGGGAAGAGGAAGAGTGCAGATGAAGAGAATTGAGAACAAAATTAATAGACAAGTTACTTTTTCAAAACGTCGATCTGGATTATTGAAGAAAGCTCATGAAATCTCTGTTCTTTGTGATGCTGAAGTTGGTTTAATTGTTTTTTCTACTAAAGGCAAACTCTGTGAGTAT---GCTACTGATTCTTGCATGGCGAGGATTCTTGAAAGATATGAAAGATACTCATATGCTGAGAGGCAGCTTGATTCTACTGATCATAGCTCC---CCGGGAAGCTGGAATCTGGAACATGCAAAACTTAAGGCAAGAATTGAGTTTTTGCAGAGAAACCAAAGGCATTATATGGGAGAAGACTTGGACTCGTTAAGTATGAAAGAACTTCAGAATTTGGAACAACAGTTAGATTCTGCTCTTAAACGCATTCGTGCAAGAAAGAACCAATTGATGCATGAGTCCATTTCTGAGCTTCAAAAAAAGGACAAAGCATTGCGAGAGCAAAACAACCTTCTTGCAAAGAAGGTGAAGGAGAGGGAGAAAGAGTTG---------------------------------------------------------------------------------------------------------------------------------------------------------------------------------

BrunfelsiaFUL1 ---------------------------------------------------------------------------------------------------------------------------------------------------------------------------------------------ATGGAGAGGATTCTTGAAAGATATGAAAGATACTCATATGCTGAGAGGCAGCTTGTTCCTACTGAAGATAGCTCC---CAGGGAGACTGGAATCTGGAACATGCAAAACTTAAGGCCAGAATTGAGATTTTGCAGAGAAACCAAAGGCATTATATGGGAGAAGACTTGGACTCATTAAGTATGAAGGAACTCCAGAATTTGGAGCACCAGCAAGATTCTGCTCTTAAACACATTCGCTCAAGAAAGAACCAATTGATGAATGAGTCCATTTCTGAGCTTAAAAAAAAGGACAAAGAATTGCAGGAGCAAAACAACCAGCTTTTGAAGAAGGTGAAGGAGAGGGAGAAAGAGCTG---------------------------------------------------------------------------------------------------------------------------------------------------------------------------------

Solanum_lycopersicumFUL1 ATGGGAAGAGGAAGAGTCCAGTTGAAGCGAATAGAGAACAAAATTAACCGTCAAGTTACCTTCTCGAAACGTCGATCTGGTTTGCTGAAGAAAGCCCATGAGATCTCTGTGCTTTGTGATGCTGAGGTTGGTTTGATTGTTTTTTCTACTAAAGGAAAACTCTTTGAATAT---GCCAACGATTCCTGCATGGAGAGGATACTTGAAAGATATGAAAGATACTCATTTGCTGAGAAACAGCTTGTTCCTACTGATCATACCTCC---CCGGTAAGCTGGACCCTTGAACATCGAAAACTTAAGGCCAGACTTGAGGTTCTGCAGAGGAACCAAAAGCATTATGTGGGAGAAGATTTGGAGTCTTTAAGTATGAAGGAACTTCAGAATCTGGAGCACCAGCTTGATTCAGCTCTTAAACACATTCGATCAAGAAAGAATCAATTGATGCATGAGTCCATTTCTGTGCTTCAAAAAAAGGACAGAGCATTGCAGGAGCAAAACAACCAGCTTTCGAAGAAGGTGAAGGAGAGGGAGAAG---------------------------------------------------------------------------------------------------------------------------------------------------------------------------------------

Cestrum_diurnumFUL1 --------------------------------------------------------------------------------------------TGCGCATGAGATGTCAGTTTTTTGTGATGCTGAGGTTGGTTTGATTGTTTTTTCTACGAAAGGCAAACTCTTTGAGTAT---GCCACTGATTCTTGCATGGAAAAGACCCTTGAAAGATATGAAAGATACTCATATGTTGAGCGCCAACTTGTTGCTACTGATCCTGCCTCT---CTGGGAAGCTGTACTTTGGAGCATGCTAAACTTAAGGCCAGACTTGAGGTTCTCCAGAGAAACCAAAAGCATTATATGGGAGAAGATTTGAATTCTTTAAGTATGAAAGAACTTCAGAATGTTGAGCACCAGCTTGATTCTTCTCTTAAACACATTCGATCAAGGAAGAACCAATTGATGCATGAGTCTATTTCTGAGCTTCAAAAGAAGGACAAGGCATTGCAGGAGCAAAATAACCAGCTTTTGAAGAAGATGAGGGAAAGGGAGAAAGAGCTA---------------------------------------------------------------------------------------------------------------------------------------------------------------------------------

Cestrum_aurantiacumFUL1 ---------------------------------------------------------------------------------------------GCGCTTGAAATGTCGATGCTTTGTGATGCTGAGGTTGGTTTGATTGTTTTTTCTACGAAAGGCAAACTCTTTGAGTAT---GCACCTGATTCTTGCATGGAAAAGATCCTTGAAAGATATGAAAGATACTCATATGCTGAGCGCCAACTTGTTGCTACTGATCCTGCCTCT---CCGGGAAGATGGACTTTGGAGCATGCGAAACTTAAGGCCAAACTTGAGGTTCTCCAGAAAAACCAAAAGCATCATATGGGAGAAGATTTGGATTCTTTAAGTATAAAAGAACTTCAGAATGTTGAGCACCAGCTTGATTCTGCTCTTAAACACGTTCGATCAAGGAAGAATCAATTGATGCATGAGTCTATTTCTGAGCTTCAAGAGAAGGACAAGGCATTGCAGGAGAAAAATAACCAGCTTTCGAAGAAGATGAAGGAAAGGGAGAAAGAGCTA---------------------------------------------------------------------------------------------------------------------------------------------------------------------------------

Cestrum_nocturnumFUL1 ATGGGAAGAGGAAGGGTGCAGTTGAAAAGAATAGAGAACAAAATAAACCGGCAAGTGACTTTCTCTAAAAGACGATCTGGTTTGCTCAAGAAAGCTCATGAGATCTCTGTGCTTTGTGATGCTGAGGTTGGTTTGATTGTTTTTTCTACGAAAGGCAAACTCTTTGAGTAT---GCCACTGATTCTTGCATGGAAAAGATCCTTGAAAGATATGAAAGATACTCATTTGCTGAGCGCCAACTTGTTGCTACTGATCCTGCCTCT---CCGGGAAGATGGACTTTGGAGCATGCGAAACTTAAGGCCAGACTTGAGGTTCTCCAGAAAAACCAAAAGCATTATATGGGAGAAGATTTGGATTCTTTAAGTATGAAAGAACTTCAGAATGTTGAGCACCAGCTTGATTCTGCTCTTAAACACGTTCGATCAAGGAAGAATCAATTGATGCATGAGTCTATTTCTGAGCTTCAAAAGAAGGACAAGGCATTGCAGGAGAAAAATAACCAGCTTTCGAAGAAGATGAAGGAAAGGGAGAAAGAGCTA---------------------------------------------------------------------------------------------------------------------------------------------------------------------------------

Nicotiana_obtusifoliaFUL1 -----------------------------------------------------------------------------------------------------------------------------------------------TTCTACAAAAGGCAAACTCTTTGAATAT---GCCACTGATTCTTGCATGGAGAGGATCCTTGAAAGATACGAAAGATACTCATATGCTGAGAGGAAGCTTGTTACTACTGATCATAGCTCC---CCGGGAAGCTGGAACCTGGAACATGCAAAACTTAAGGCTAGAGTTGAGGTTTTACAGAGAAACCAAAGGCATTATATGGGAGAAGATTTGGACTCGTTAAGTACGAAAGAACTTCAGAATTTGGAGCAGCAGCTGGATTCTGCTCTTAAACTCATTCGCTCAAGAAAGAATCAATTGATGCATGAGTCTATTTCTGAGCTTCAAAAAAAGGACAAAGCACTGCAGGAGCAAAACAACCAGCTTTCCAAGAAGGTGAAGGAGAGGGAGAAAGAGCTG---------------------------------------------------------------------------------------------------------------------------------------------------------------------------------

GrabowskiaFUL1 ATGGGGAGAGGAAGAGTGCAGCTGAAGAGAATAGAGAACAAAATTAATCGACAAGTGACTTTCTCTAAACGTCGATCTGGTTTGTTGAAGAAAGCCAATGAGATCTCTGTGCTTTGTGATGCTGAGGTTGGTTTGATTGTTTTTTCTACTAAAGGCAAACTCTTTGAATAT---GCTACTGATTCTTGCATGGAAAGGGTGCTTGAAAGATATGAAAGATACTCATACGCTGAGAGGCAGCTTGTTCCTACTGATCCTACCTCC---CCGGGAAGCTGGACTCTGGAACATGCAAAACTTAAGGCCAGACTTGAGGTTTTGCAAAGAAACCAAAAGCATTATATGGGAGAAGACTTGGACTTATTAAGTATGAAAGAACTTCAGAATGTGGAGCACCAGCTTGATTCTGCTCTTAAACACATTCGCTCAAGAAAGAGCCAATTGATGCATGAGTCCATTTCTGTGCTTCAAAAAAAGGACAAAGCATTGCAGGAGCAAAACAACCAGCTTTCCAAGAAGGTGAAGGCAAAGGAGAA----------------------------------------------------------------------------------------------------------------------------------------------------------------------------------------

Nicotiana_langsdorffii_sanderaeFUL1 ------------------------------------------------------------------------------------------------------------------------------------------------------------------------------------------------------------------------------------------------------------------------------------------------------------------------------------------------------------------------------------------------------------------------------------------TCAAGAAAGAACCAATTGATGCATGAGTCCATTTCTGAGCTTCAAAAAAAGGACAAAGCACTGCAGGAGCAAAACAACCAGCTTTCCAAGAAGGTGAAGGAGAGGGAGAAAGAGCTG---------------------------------------------------------------------------------------------------------------------------------------------------------------------------------

Nicotiana_tabacumFUL1 ATGGGAAGAGGAAGGGTGCAGTTGAAGAGAATTGAGAACAAAATTAATAGGCAAGTTACTTTCTCAAAACGTCGATCTGGTTTGCTTAAGAAAGCTCATGAGATCTCTGTGCTTTGTGATGCTGAGGTTGGTTTGATTGTTTTTTCTACAAAAGGCAAACTCTTTGAATAT---GCCACTGATTCTTGCATGGAGAGGATCCTTGAAAGATACGAAAGATACTCATATGCTGAGAGGCAACTTGTTACTACTGATCATAGCTGC---CCGGGAAGCTGGACCCTGGAACATGCAAAACTTAAGGCTAGACTTGAGGTTTTGCAGAGAAACCAAAGGCATTATACGGGAGAAGATTTGGACTCGTTAAGTACGAAGGAACTTCAGAATTTGGAACACCAGCTGGATTCTGCTCTTAAACACATTCGCTCAAGCAAGAACCAATTGATGCATGAGTCTATTTCTGAGCTTCAAAAAAAGGACAAAGCACTGCAGGAGCAAAACAACCAGCTTTGCAAGAAGGTGAAGGAGAGGGAGAAAGAGTTG---------------------------------------------------------------------------------------------------------------------------------------------------------------------------------

Juanalloa_mexicanaFUL1 --------------------------------------------------------------------------------------------TGCGCTTGAGATATCGGTTCTGTGCGATGCTGAGGTTGGTTTGATTGTTTTTTCTAGTAAAGGCAAACTCTTTGAATAT---GCCACTGAATCATGCATGGAAAGGATACTTGAAAGATATGAAAGATACTCATTTGCTGAGAGACAGCTTGTTCCTACTGATCATAGCTCC---CCGGGAAGCTGGACTCTGGAACAGGCAAAACTTAAGGCCAGACTTGAGGTTCTGCAGAGGAACCGAAAGCATTATGTGGGAGAAGATTTGGACTCGTTAACTATGAAAGAACTTCAGAATCTGGAGCACCAGCTTGATTCTGCTCTTAAACACATTCGATCAAGAAAGAACCAATTGATGCATGAGTCCATTTCTGTGCTTCAAAAAAAGGACAAAGCATTGCAGGAGCAAAACAACCTGCTTTCCAAGAAGGTGAAGGAGAGGGAGAAAGAGCTG---------------------------------------------------------------------------------------------------------------------------------------------------------------------------------

Datura_inoxiaFUL1 --------------------------------------------------------------------------------------------------TGAGCTGTCGGTGCTATGCGATGCTGAGGTTGGTTTGATTGTTTTTTCCACTAAAGGCAAACTCTTTGAATAC---GCTACAGATTCTTGCATGGAAAGGATACTGGAAAGATATGAAAGATACTCATTTGCTGAGAGGCAGGTTGCTCCTACTGATCATACCTCC---CCGAGAAGCTGGATTCTGGAACAGGCAAAACTTAAGGCCAGACTTGAGGTTCTGCAGGGGAACCAAAAGCATTATGTTGGAGAAGATTTGGAGTCATTAAATATGAAAGAACTTCAGAATCTGGAACACCAGCTTGATTCTGCTCTCAAACACATAAGATCAAGAAAGAACCAATTGATGCATGAGTCCATTTCTGTGCTTCAAAAAAAGGACAAAGCACTGCAGGACCAAAACAACCAGCTTTCCAAGAAGGTGAAGGAGAGGGAGAAAGAGTTG---------------------------------------------------------------------------------------------------------------------------------------------------------------------------------

Nicandra_physalodesFUL1 -------------------------------------------------------------------------------------------------ATGAAATTTCGGTGCTGTGTGATGCCGAGGTTGGTTTGATTGTTTTCTCAACTAAAGGGAAACTCTTTGAATAT---GCTACCGATTCTTGCATGGAAAGGATACTTGAAAGATATGAAAGATACTCATTTGCTGAGAGGCAGCTTGCTCCTACTGATCATAGCACC---CCGGGAAGTTGGACTCTGGAACACGCAAAACTTAAGGCCAGACTTGAGGTTCTCCAGAGGAACCAAAAGCATTATGTGGGAGAAGATTTGGACTCGTTAAATATGAAAGAACTTCAGAATCTGGAACATCAGCTTGATTCTGCTCTTAAACATATTCGATCAAGAAAGAACCAATTGATGCATGAGTCAATTTCTGTGCTTCAAAAAAAGGACAAAGCATTGCAGGAGCAAAACAACCAGCTTTCCAAGAAGGTGAAGGAGAGGGAGAAAGAAATG---------------------------------------------------------------------------------------------------------------------------------------------------------------------------------

DunaliaFUL1 ------------------------------------------------------------------------------------------------------------------------------------------------TCTACTAAAGGCAAACTTTTTGAATAT---GCCAATGATTCTAGCATGGAAAGGATACTTGAAAGATATGAAAGATACTCATATGCTGAGAGGCAGCTTGTTCCTACTGATCATTCCTCC---CCGGAAAGCTGGACTCTGGAGCATGCAAAACTTAAGGCCAGACTTGAGGTTCTACAGAGGAACCAAAAGCATTACGTGGGAGAAGATTTGGAGTCGTTAAATATGAAAGAACTTCAGAATCTGGAGCACCAGCTTGATTCTGCTCTTAAACACATTCGATCAAAGAAGAACCAATTGATGCATGAGTCCATTTCTGTGCTTCGAAAAAAGGACAAAGCATTGGCGGAGCAAAACAACCAACTTTCCAAGAAGGTGAAGGAGAGGGAGAAAGAGCTG---------------------------------------------------------------------------------------------------------------------------------------------------------------------------------

Iochroma_fuchsiodeasFUL1 ---------------------------------------------------------------------------------------------GCGCTTGAGCTTTCGGTTTTTTGTGATGCTGGGGTTGGTTTGATTGTTTTTTCTACTAAAGGCAAACTCTTTGAATAT---GCCAATGATTCT---------------------------------------------------------------------------------NAGGGAAGCTGGACTCTGGAGCATGCAAAACTTATGGCCAGACTTGAGGTTCTGCAGAGGAACCAAAAGCATTATGTGGGAGAAGATTTGGAGTTGTTAAATATGAAAGAACTTCAGAATCTGGAGCACCAGCTTCATTCTGCTCTTAAACACATTCGATCAAAGAAGAACCAATTGATGCATGAGTCCATTTCTGTGCTTCAAAAAAAGGACAAAGCATTGGCGGAGCAAAACAACCAACTTTCCAAGAAGGTGAAGGAGAGGGAGAAAGAGCTG---------------------------------------------------------------------------------------------------------------------------------------------------------------------------------

Solanum_cheesmaniiFUL1 ATGGGAAGAGGAAGAGTGCAGCTGAAGAGAATAGAGAACAAAATAAATCGACAAGTTACTTTCTCAAAACGTCGATCTGGTTTGTTGAAGAAAGCTCATGAGATCTCTGTGCTTTGTGATGCTGAGGTTGGTTTGATTGTTTTTTCAAATAAAGGAAAACTCTTTGAATAC---GCCAATGATTCCTGCATGGAAAGGACACTTGAAAGATATGAAAGATACTCATTTGCTGAGAGGCAGCTTGTCCCTGCTGATCAAACCTCC---CCGGGAAGCTGGACTCTGGAACATGCAAAACTTAAGGCCAGACTTGAAGTTCTGCAGAGGAACCAAAAGCATTATGTGGGAGAGGATTTGGATTCGTTAAATATGAAAGAACTTCAGAATCTGGAGCATCAACTTGATTCTGCTCTTAAACATATGAGATCAAGAAAGAACCAATTGATGCATGAGTCCATTTCTGTGCTTCAAAAAAAGGACAAAGCATTGCAGGATCAAAACAACCAGCTTTCCAAGAAGGTGAAGGAGAAGGAGAAAGAGGTG---------------------------------------------------------------------------------------------------------------------------------------------------------------------------------

Solanum_pimpinellifoliumFUL1 ATGGGAAGAGGAAGAGTCCAGTTGAAGCGAATAGAGAACAAAATTAACCGTCAAGTTACCTTCTCGAAACGTCGATCTGGTTTGCTGAAGAAAGCCCATGAGATCTCTGTGCTTTGTGATGCTGAGGTTGGTTTGATTGTTTTTTCTACTAAAGGAAAACTCTTTGAATAT---GCCAACGATTCCTGCATGGAGAGGATACTTGAAAGATATGAAAGATACTCATTTGCTGAGAAACAGCTTGTTCCTACTGATCATACCTCC---CCGGTAAGCTGGACCCTTGAACATGCAAAACTTAAGGCCAGACTTGAGGTTCTGCAGAGGAACCAAAAGCATTATGTGGGAGAAGATTTGGAGTTCTTAAGTATGAAGGAACTTCAGAATCTGGAGCACCAGCTTGATTCAGCTCTTAAACACATTCGATCAAGAAAGAATCAATTGATGCATGAGTCCATTTCTGTGCTTCAAAAAAAGGACAGAGCATTGCAGGAGCAAAACAACCAGCTTTCGAAGAAGGT-------------------------------------------------------------------------------------------------------------------------------------------------------------------------------------------------------

Solanum_dulcamaraFUL1 --------------------------------------------------------------------------------------------------TGAGATCTCTGTGCTTTGTGATGCTGAGGTTGGTTTGATTGTTTTTTCCACTAAAGGAAAACTCTTTCAATAT---ACCAATGATTCCTGCATGGAAAGGATACTTGAAAGATATGAAAGATACTCATTTGCTGAGAGGCAGCTTGTTCCTACTGATCATACCTCC---CCGGGAAGCTGGACTCTGGAACATGCAAAACTTAAGGCGAGACTTGAGGTTCTGCAGAGGAACCAAAAGCATTACGTAGGAGAAGATTTGGAATCGTTAAATATGAAAGAACTTCAGAAACTGGAACAACAACTTGATTCTTCTCTTAAACACATTCGATCAAGAAAGAACCAACTGATGCATGAGTCTATTTCTGTGCTTCGAAAAAAGGACAAAGCATTGCAGGAGCAAAACAACCAGCTTTCCAAGAAGGTGAAGGAGAGGGAGAAAGAAGGG---------------------------------------------------------------------------------------------------------------------------------------------------------------------------------

Solanum_commersoniiFUL1 --------------------------------------------------------------------------------------------------TGAGATCTCTGTGCTTTGTGATGCTGAGGTTGGTTTGATTGTTTTTTCCACTAAAGGAAAACTCTTTGAATAT---GCAACTGATTCATGCATGGAGAGGTTACTTGAAAGATATGAAAGATACTCATTTGCTGAGAAGCAGCTTGTTCCTACTGATCATACATCC---CCGGGAAGCTGGACTCTTGAAAATGCAAAACTTAAGGCCAGACTTGAGGTTCTGCAGAGGAACGAAAAGCTTTATGTGGGAGAAGATTTGGAGTCGTTAAATATGAAAGAACTTCAGAATCTTGAACACCAGCTTGCTTCTGCTCTTAAACACATTCGATCAAGAAAGAACCAATTGATGCATGAGTCCATTTCTGTGCTTCAAAAACAGGACAGAGCATTGCAGGAGCAAAACAACCAGCTTTCCAAGAAGGTGAAGGAGAGGGAGAAAGAGGTG---------------------------------------------------------------------------------------------------------------------------------------------------------------------------------

Solanum_tuberosum_FUL1 --------------------------------------------------------------------------------------------AGCTCATGAGATCTCTGTGCTTTGTGATGCTGAGGTTGGTTTGATTGTTTTTTCCACTAAAGGAAAACTCTTTGAATAT---GCCAATGATTCATGCATGGAGAGGCTACTTGAAAGATATGAAAGATACTCATTTGCTGAGAGGCAGCTTGTTCCTACTGATCATACATCC---CCGGGAAGCTGGACTCTGGAACATGCAAAACTTAAGGCCAGACTTGAGGTTCTTCAGAGGAACCAAAAGCATTATGTGGGAGAAGATTTGGAGTCGTTAAATATGAAAGAACTTCAGAATCTTGAACACCAGCTTGATTCTGCTCTTAAACACATTCGATCAAGAAAGAACCAATTGATGCATGAGTCCATTTCTGTGCTTCAAAAACAGGACAGAGCATTGCAGGAGCAAAACAACCAGCTTTCCAAGAAGGTGAAGGAGAGGGAGAAAGAGGTG---------------------------------------------------------------------------------------------------------------------------------------------------------------------------------

Solanum_betaceumFUL1 --------------------------------------------------------------------------------------------TGCGCTTGAACTGTCTATGCTCTGCGATGCTGAGGTTGGTTTGATTGTTTTTTCCACTAAAGGAAAACTCTTTGAATAT---GCGAATGATTCCTGCATGGAAAGGATACTTGAAAGATATGAAAGATACTCATTTGCTGAGAGGCAGTTTGTTCCTACTGATCATACCTCC---CCGGGAAGCTGGACTCTGGAACATGCAAAACTTAAGGCCAGACTTGAAGTTCTGCAGAGGAACCAAAAGCATTATGTGGGAGAGGATTTGGAGTCATTAAATATGAAAGAACTTCAGAATCTGGAGCACCAACTTGATTCTGCTCTTAAACACATTCGATCAAGAAAGAACCAATTGATGCATGAGTCCATTTCTGTGCTTCAAAAAACGGACAAAGCATTGCAGGAGCAAAACAACCAGCTTTCCAAGAAGGTGAAGGAGAGGGAGAAAGAGGTG---------------------------------------------------------------------------------------------------------------------------------------------------------------------------------

Solanum_quitoenseFUL1 ------------------------------------------------------------------------------------------GCGCTTGAGATGTCTGTCTTTTGCGATGCTGAGGTTGGTTTGATTGTCTTTTCAAATAAAGGAAAACTCTTTGAATAT---GCCAATGATTCCTGCATGGAAAGGATACTCGAAAGATATGAAAGATACTCATTTGCTGAGAGGAAGCTTGTTCCTACTGACCATACCTCG---TCGGGAAGCTGGACTCTGAAACATGCAAAACTTAAGGCTAGACTTGAGGTTCTGCAGAGGAACCAAAAGCATTATGTGGGAGAGGATTTGGAGTTATTAAATATGAAAGAACTTCAGAATCTGGAGCACCAACTTGATTCTGCTCTTAAACACATAAGATCTAGAAAGAACCAAGTGATGCATGAGTCCATTTCTGTGCTTCAAAAAAAGGACAAAGCATTGCAGGAGCAAAACAATCAGCTTTCCAAGAAGATGAAAGAGAGGGAGAAAGAGGTG------------------------------------------------------------------------------------------------------------------------------------------------------------------------------------

StreptosolenFUL2 ATGGGGAGAGGAAGAGTGCAAATGAAGAGAATTGAGAACAAGATCAATAGGCAAGTTACTTTCTCGAAGAGGAGAAGTGGGTTGCTGAAGAAAGCTCATGAGATCTCTGTGCTTTGTGATGCTGAGGTTGGTTTGATTGTTTTTTCCACTAAAGGAAAACTCTTTGAGTAC---TCTACTGATTCTTGCATGGAAAGGATTCTTGAAAGGTACGAACGATACTCATATGCTGAGAGGCAGCTCAATCCTGCTGATCAGGACTCC---CCGGCTAGCTGGACTCTGGAGCATGCTAAGCTTAAGGCTAGAATTGAGGTTTTGCAAAGAAACCAAAGGCATTATGCTGGAGAAGAACTGGACTCTCTAAGTATGAAAGAACTTCAGAATCTGGAGCATCAGCTCGATTCTGCTGTCAAACACATTCGATCAAGAAAGAATCAATTGATGCATGAATCTATTTCTGAGCTGCAAAAGAAGGACAAGGCATTGCAAGAGCAAAACAACAAGCTCACGAAGCAGGTTAAGGAAAGAGAAAAAGAGATTGCTCAGCAGAATCAGTGGGAGCAACAAAACCATGATCATCTCAACTCATCTTCATTTGTGTTGTCACAGCCTATGAACTCTCTTCACATTGGGGAAGCATACCCGGCTGCAGGAGACAATGGAGAAATTGAAGGATCTTCGCGGCATCAACCACCTAACGTGATGCCGCCATGGATG

Petunia_exsertaFUL2 --------------------------------------------------------------------------------------------------------GTCGGTGCTCTGCGATGCTGAAGTTGGACTAATTGTTTTCTCCACTAAAGGCAAACTCTTTGAGTAT---TCTACTGATTCTTGCATGGAAAGGATTCTTGAAAGGTATGAAAGATACTCATATGCTGAGAGGCAGCTTAGTGCCACTGATAATGATACT---CCGGGGAGCTGGACTCTGGAACATGCTAAGCTTAAGGCCAGGCTTGAAGTATTGCAAAGAAACCAAAAGCATTATGCGGGAGAAGACTTGGATTCCTTAAGCATGAAAGAGCTTCAAAATTTGGAGCAGCAGCTCGATTCTGCTCTTAAACAGATTCGATCAAGAAAGAACCAATTGATGCATGAGTCTATTTCTGAGCTGCAAAAGAAGGACAAGGCATTGCAAGAGCAAAACAACAAGCTCTCGAAGCAGGCGAAGGAAAGGGAGAAAGAGCTAGCCCAGCAGAGTCAGTGGGAACCACAGAGTCATGAT---CTCAACTCATCTTCATTCGTTTTGTCACAGCCCTTGAACTCTCTTCACCTTGGGGAAGCATACCCTAGTGCAGGAGACAATGGAGAAGTTGAAGGGTCTTCAAGGCAGCAACCACCAAACGTGATGCCCCCCTGGATG

Petunia_hybridaFUL2 ATGGGGAGAGGAAGAGTGCAACTCAAGAGAATTGAAAACAAAATCAATCGACAAGTTACGTTTTCGAAACGACGATCTGGGTTGTTGAGGAAAGCTCATGAGATTTCTGTGCTTTGTGATGCTGAAGTTGGACTAATTGTTTTCTCCACTAAAGGCAAACTCTTTGAGTAT---TCTACTGATTCTTGCATGGAAAGGATTCTTGAAAGGTATGAAAGATACTCATATGCTGAGAGGCAGCTTAGTGCCACTGATAATGATACT---CCGGGGAGCTGGACTCTGGAACATGCTAAGCTTAAGGCCAGGCTTGAAGTTTTGCAAAGAAACCAAAAGCATTATGCGGGAGAAGACTTGGATTCCTTAAGCATGAAAGAGCTTCAAAATTTGGAGCAGCAGCTCGATTCTGCTCTTAAACAGATTCGATCAAGAAAGAACCAATTGATGCATGAGTCTATTTCTGAGCTGCAAAAGAAGGACAAGGCATTGCAAGAGCAAAACAACAAGCTCTCGAAGCAGGTGAAGGAAAGGGAGAAAGAGCTAGCCCAGCAGAGTCAGTGGGAACCACAGAGTCACGAT---CTCAACTCATCTTCATTCGTTTTGTCACAGCCCTTGAACTCTCTTCACCTTGGGGAAGCATACCCTAGTGCAGGAGACAATGGAGAAGTTGAAGGGTCTTCAAGGCAGCAACCACCAAACGTGATGCCCCCATGGATG

Plowmania_nyctaginoidesFUL2 ---------------------------------------------------------------------------------------------GCGCTTGAAATATCGGTTCTTTGTGATGCTGAAGTTGGTTTAATTGTTTTTTCTACTAAAGGCAAACTCTTTGAGTAC---TCTACTGATTCTTGCATGGAAAGGATTCTTGAGAGGTATGAAAGATATTCATATGCTGAGAGGCAGCTTAGTACTACTGATCAAGACACC---CCGGGAAGCTGGACACTGGAACATGCTAAGCTTAAGGCCAGACTTGAGATTTTGCAAAGAAACCAAAATCATTACGCGGGAGAGGATTTGGACACATTGAGTATGAAAGAGCTTCAAAATCTGGAGCACCAGCTTGATTCTGCTCTCAAACACGTTCGATCAAGAAAGAACCAATTGATGCATGAATCCATTTCTGAGCTGCAAAAGAAGGACAAGGCATTGCAAGAGCAAAACAACAAGCTCTCGAAGCAGGTTAAGGNAAGGGAGAAAGAGCTGGCACAGCTGAATCAGTGGGAGCAACAGCACCATGAT---TTCAATTCATCTTCATTCATTTTGTCACAGCCCTTGAACTCTCTTCACCTTGGTGAAGCATACCCAACTGCAGGAGACAATGGAGAAGTTGAAGGATCTTCGCGGCAGCAGCACCCGCCAGTGATGCCCCCCTGGATG

Withania_somniferaFUL2 --------------------------------------------------------------------------------------------------TGAGATGTCGATGTTGTGCGATGCTGAAGTTGGTTTGATTGTTTTCTCAAATAAAGGCAAACTATTTGAGTAT---TCTACTGATTCTTGCATGGAAAGAATTCTTGAAAGGTATGAAAGGTACTCATATGCTGAGAGGCAGCTTACTGCTACTGATGTTGAAACC---CCGGGGAGCTGGACTTTGGAACATGCTAAACTTAAGGCCAGACTTGAGGTTTTGCAAAGAAACCAAAGGCATTATGCGGGAGAGGACTTGGACTCGTTGAGTATGAAAGAGCTTCAGAATCTGGAGCACCAGCTTGATTCTGCTATTAAGCATATTAGATCAAGAAAGAACCAATTGATGCATGAATCCATTTCTGAGCTGCAAAAGAAGGACAAAGCATTGCAAGAACAAAACAACAATCTTTCAAAGAAGGTGAAAGAAAGGGAGAAAGAGTTGGCCCAACGGACTCCATGGGAGCAACAGAGCCATGATCATCTCAACTCATCTTCATTCCTTTTGCCACACCCCTTGAACGATCTTCATCTAGGGGAAGCATACCCAACTGCCGGAGACAATGGAGAAGTTGAAGGATCATCGCAGCAGCAGCCACAAAACGTGATGCCCCCCTGGATG

Physalis_pubescensFUL2 --------------------------------------------------------------------------------------------------TGAGATCTCCGTGCTTTGTGATGCTGAAGTTGGTTTGATCGTTTTCTCAAATAAAGGCAAACTATTTGAGTAT---TCTACTGATTCTTGCATGGAAAGAATTCTTGAAAGGTATGAGAGGTACTCATATGCTGAGAGGCAGCTTAATGCTACTGATATCGAAACC---CCGGGAAGCTGGACTTTGGAACATGCTAAGCTTAAGGCCAGACTTGAGGTTTTGCTAAGAAACCAAAGGCATTATGCGGGAGAGGACTTGGACTCATTGGGTATGAAAGAGCTTCAGAATCTGGAGCACCAGCTTGATTCTGCTCTTAAGCATATTAGATCAAGAAAGAACCAATTGATGCATGAATCCATTTCTGAGCTGCAAAAGAAGGACAAGGCATTGCAGGAACAAAACAACAATCTTACGAAGAAGGTGAAGGAAAGGGAGAAAGAGTTGGCCCAGCGGACTCCGTGGGAGCAGCAGAGCCATGATCATCTCAACTCATCTTCATTCGTTTTGCCACACCCCTTGAACAGCCACCACCTTGGGGAAGCATACCCAACTGCAGGAGACAATGGAGAAGTTGAAGGATCCTCGCAGCAGCAGCAGCAACAAGTGATGCCGCCATGGATG

Nicotiana_sylvestrisFUL2 ATGGGGAGAGGAAGAGTGCAACTGAAGAGAATTGAGAACAAGATCAATCGACAAGTCACCTTCTCAAAAAGAGCATCTGGTTTGCTTAAGAAAGCTCATGAAATCTCTGTGCTTTGTGATGCTGAGGTTGGTTTAATTGTTTTTTCTACTAAAGGGAAACTCTTTGAGTAT---TCCACTGATTCTTGCATGGAAAGGATTCTTGAAAGGTATGAAAGGTACTCATATGCTGAGAGGCAGCTTACTGCTACTGATGATGAAACC---CCGGGGAGCTGGACTTTGGAACATGCTAAGCTTAAGGCCAGACTTGAGGTTTTGCAAAGAAACCAAAGGCATTATGCAGGAGAAGATTTGGACTCATTAAGTATGAAAGAGCTTCAGAATCTTGAGCACCAGCTCGATTCTGCTCTTAAGCACATTCGATCAAGAAAGAATCAATTGATGCATGAATCCATTTCTGAGCTGCAAAAGAAGGACAAGGCATTGCAAGAGCAAAACAACAATCTCTCAAAGCAGGTGAAAGAAAGGGAGAAAGAGCTAGCTCAGCAGACTCAATGGGAGCAACAGAGCCATGATCATCTCAACTCATCTTCATTCGTTTTAACACAGCCCTTGAGCTCTCTTCACCTCGGGGAAGCGTACCCGACTGCAGGAGACAACGGAGAAGTGGAAGGATCATCGCGGCAACAACAACAAAACGTGATGCCGCCATGGATG

Nicotiana_obtusifoliaFUL2 ATGGGGAGAGGAAGAGTGCAACTGAAGAGAATTGAGAACAAGATCAATCGACAAGTCACCTTCTCAAAAAGGCGATCTGGGTTGCTCAAGAAAGCTCATGAGATCTCTGTGCTTTGTGATGCTGAGGTTGGTTTAATTGTTTTTTCTACAAAAGGCAAACTCTTTGAGTAT---TCCACCGATTCTTGCATGGAAAGGATTCTTGAAAGGTATGAAAGATACTCATATGCCGAGAGGCAGCTTACTGCTACTGATCATGAAACC---CCGGGGAGCTGGACTTTGGAACATGCTAAGCTTAAGGCAAGACTTGAGGTTTTGCAAAGAAACCAAAGGCATTATGCAGGAGAAGATTTGGACACATTAAGTATGAAAGAGCTGCAGAATCTTGAGCACCAGCTCGATTCTGCTTTAAAGCACATTCGATCAAGAAAGAATCACTTGATGCATGAATCCATTTCTGAGCTGCAAAAGAAGGACAAGGCATTGCAAGAGCAAAACAACAAGCTCTCGAAGCAGGTGAAAGAAAGGGAGAAAGAGATGGCTCAGCAGACTCAGTGGGAGCAACAGAGCCATGATCATCTCAACTCATCTTCATTCGTTTTGTCACAGCCCTTGAGCTCTCTTCACCTTGGGGAAGCGTACCCGACTGCAGGAGACAACGGAGAAGTTGAAGGATCATCGCGGCAACAACAACAGAACGTGATGCCGCCATGGATG

Nicotiana_tabacumFUL2 ATGGGGAGAGGAAGAGTGCAACTGAAGAGAATTGAGAACAAGATCAATCGACAAGTCACCTTCTCAAAAAGACGATCTGGTTTGCTCAAGAAAGCTCATGAGATCTCTGTACTTTGTGATGCTGAGGTTGGTTTAATTGTTTTTTCTACAAAAGGCAAACTCTTTGAGTAT---TCCACCGATTCTTGCATGGAAAGGATTCTTGAAAGGTATGAAAGGTACTCATATGCTGAGAGGCAGCTTACTGCTACTGATCATGAAACC---CCGGGGAGCTGGACTTTGGAACATGCTAAGCTTAAGGCAAGATTTGAGGTTTTGCAAAGAAACCAAAGGCATTATGCAGGAGAAGATTTGGACTCATTAAGTATGAAAGAGCTGCAGAATCTTGAGCACCAGGTCGATTCTGCTTTAAAGCACATTCGATCAAGAAAGAATCAATTGATGCATGAATCCATTTCTGAGCTGCAAAAGAAGGACAAGGCATTGCAAGAGCAAAACAACAAGCTCTCGAAGCAGGTGAAAGAAAGGGAGAAAGAGCTGGCTCAGCAGACTCAGTGGGAGCAACAGAGCCATGATCATCTCAACTCATCTACATTCGTTTTGTCACAGCCCTTGAGCTCTCTTCACCTTGGGGAAGCGTACTCAACTGCAGGAGACAACGGAGAAGTTGAAGGATCATCGCGGCAACAACAACAGAACGTAATGCCGCCATGGATG

Nicotiana_tomentosiformisFUL2 --------------------------------------------------------------------------------------------------TGAGATCTCTGTACTTTGTGATGCTGAGGTTGGTTTAATTGTTTTTTCTACAAAAGGCAAACTCTTTGAGTAT---TCCACCGATTCTTGCATGGAAAGGATTCTTGAAAGGTATGAAAGGTACTCATATGCTGAGAGGCAGCTTACTACTACTGATCATGAAACC---CCGGGGAGCTGGACTTTGGAACATGCTAAGCTTAAGGCAAGAATTGAGGTTTTGCAAAGAAACCAAAGGCATTATGCAGGAGAAGATTTGGACTCATTAAGTATGAAAGAGCTGCAGAATCTTGAGCACCAGGTCGATTCTGCTTTAAAGCACATTCGATCAAGAAAGAATCAATTGATGCATGAATCCATTTCTGAGCTGCAAAAGAAGGACAAGGCATTGCAAGAGCAAAACAACAAGCTCTCGAAGCAGGTGAAAGAAAGGGAGAAAGAGCTGGCTCAGCAGACTCAGTGGGAGCAACAGAGCCATGATCATCTCAACTCATCTACATTCGTTTTGTCACAGCCCTTGAGCTCTCTTCACCTTGGGGAAGCGTACTCAACTGCAGGAGACAACGGAGAAGTTGAAGGATCATCGCGGCAACAACAACAGAAC------------------

Solanum_pimpinellifoliumFUL2 ATGGGTAGAGGAAGAGTACAATTGAAGAGAATTGAGAACAAAATTAATCGTCAAGTTACTTTTTCAAAGAGGCGATCTGGTTTGCTTAAAAAAGCTCATGAGATCTCTGTGCTTTGCGATGCTGAAGTTGGACTCATTGTTTTCTCAACTAAAGGAAAACTCTTTGAGTAT---TCTACTGACTCTTGCATGGAAAGGATTCTTGAAAGGTATGAAAGGTACTCATATGCTGAAAGGCAGCTTAATGCTACTGATATTATAACC---CCGGGTAGCTGGACTTTGGAACATGCTAAGCTTAAGGCCAGACTTGAGGTTTTGCAAAGAAACCAAAAGCATTATGCAGGAGAAGAGTTGGACACATTGAGTATGAAAGAGCTTCAGAATCTGGAACACCAGCTCGATTCTGCTCTTAAGCACATTCGATCTAGAAAGAACCAATTGATGCATGAATCCATTTCTGAGCTTCAAAAGAAGGACAAGGCATTGCAAGAACAAAACAACAATCTTTCAAAGCAGGTTAAGGAAAGGGAGAAAGAGATGGCCCAACAGACTCCGTGGGAGCAACAGAGTCATGATCATCTCAATTCATCTTCGTTTGTTTTGCCACACCCCTTTAACAATCTTCACATAGGGGAAGCATACCCAAATGCAGGAGACAATGGAGAAGTAGAAGGATCATCGCGGCAACAACAACAAAACGTGATGCCTCCATGGATG

Solanum_tuberosumFUL2 --------------------------------------------------------------------------------------------------TGAGATCTCTGTGCTTTGCGATGCTGAAGTTGGACTCATTGTTTTTTCAACTAAAGGAAAACTCTTTGAGTAT---TCCACCGACTCTTGCATGGAAAGGATTCTTGAAAGGTATGAAAGGTACTCATATGCTGAAAGGCAGCTTAATGCTACTGATATTGAAACC---CCGGGGAGCTGGACTTTGGAACATGCTAAGCTTAAGGCCAGACTTGAGGTTTTGCAAAGAAACCAAAAGCATTATGCAGGAGAAGAGTTGAACACATTGAGTATGAAAGAGTTTCAGAATCTGGAACACCAGCTCGATTCTGCTCTTAAGCACATTCGATCAAGAAAGAACCAATTGATGCATGAATCCATTTCTGCGCTGCAAAAGAAGGACAAGGCATTGCAAGAACAAAATAACAATCTTTCAAAGCAGGTGAAGGAAAGGGAGAAAGAGATGTCCCAACAGACTCCGTGGGAGCAACAGAGTCATGATCATCTCAATTCATCTTCGTTTGTTTTGCCACACCCCTTTAACAACCTTCACATGGGGGAAGCATACCCAACTGCAGGAGACAATGGAGAAGTTGAAGGATCATCGCGGCAGCAACAACAAAAC------------------

Solanum_sisymbriifoliumFUL2 -------------------------------------------------------------------------------------------------------------------------------------------------------------------------------------------GCATGGAAAGGATTCTTGAAAGGTATGAAAGGTACTCATATGCTGAGAGGCAGCTTAATGCTACTGATATCGAAACC---CCGGGGAGCTGGACTTTGGAACATGCTAAGCTTAAGGCCAGACTTGAGGTTTTGCAAAGAAACCAAAAGCATTATGCAGGAGAAGAGTTGGACTCATTGAGTATGAAAGAGCTTCAGAATCTGGAGCACCAGCTCGATTCTGCTCTTAAGCACATTCGATCAAGAAAGAACCAATTGATGCATGAATCCATTTCTGAGCTGCAAAAGAAGGACAAGGCATTGCAAGAACAAAACAACAATCTTTCAAAGCAGGTGAAGGAAAGGGAGAAAGAGCTGGCCCAGCAAACTCCATGGGAGCAACAGAGTCATGACCATCTCAATTCATCTTCATTCGTTTTGCAACACCCCTTTAACAACCTTCACTTAGGGGAAGCATACCCAACTGCAGGAGACAATGGGGAAATTGAAGGATCATCGAGGCAGCAACAACAAAACGTGATGCCGCCATGGATG

Solanum_xanthocarpumFUL2 ATGGGAAGAGGAAGAGTACAGCTTAAGAGAATTGAAAACAAAATCAATCGTCAAGTCACTTTTTCAAAGAGGCGATCTGGTTTACTCAAGAAAGCTCATGAGATCTCTGTGCTTTGTGATGCTGAAGTTGGACTCATTGTTTTTTCAACTAAAGGAAAATTATTTGAGTAT---TCAACAGACTCATGCATGGAAAGGATTCTTGAAAGGTATGAAAGGTACTCATATGCTGAGAGGCAGCTTAATGCTACTGATATCGAAACC---CCGGGGAGCTGGACTTTGGAACATGCTAAGCTTAAGGCCAGACTTGAGGTTTTGCAAAGAAACCAAAAGCATTATGCAGGAGCAGAGTTGGACTCATTGAGTATGAAAGAGCTTCAGAATCTGGAGCACCAGCTCGATTCTGCTCTTAAGCACATTCGATCAAGAAAGAACCAATTGATGCATGAATCCATTTCTGAGCTGCAAAAGAAGGACAAGGCATTGCAAGAACAAAACAACAATCTTTCAAAGCAGGTGAAGGAAAGGGAGAAAGAGCTGGCCCATCAAACTCCTTGGGAGCAACAGAGTCATGATCATCTCAATTCATCTTCGTTCGTTTTGCCACACCCCTTTAACAACCTTCACTTAGGGGAAGCATACCCAACTGCAGGAGACAACGGAGAAATTGAAGGATCATCGAGGCAGCAACAACAAAACGTGATGCCGCCATGGATG

Solanum_dulcamaraFUL2 ----------------------------------------------------------------------------------------------------------------------------------------------------------------------------------------------------------------------------------------------------------------------CC---CCGGGGAGCTGGACTTTGGAATATGCTAAACTTAAGGCCAGACTTGACGTTTTGCAAAGAAACCAAAAGCATTATGCAGGAGAAGAGTTGGACTCATTGAGTATGAAAGAGCTTCAAAATCTGGAACACCAGCTCGATTCTTCTCTTAAGCATATTCGATCGCGAAAGAACCAATTGATGCATGAATCCATTTCTGAGCTGCAAAAGAAGGACAAGGCACTGCAAGAACAAAACAACAATCTTTCAAAGCAGGTGAAGGAAAGGGAGAAAGAGATGGCCCAGCAGACTCCGTGGGAGCAACA-------------------------------------------------------------------------------------------------------------------------------------------------------

Solanum_ptychanthumFUL2 ATGGGGAGAGGAAGAGTACAACTTAAGAGAATTGAAAACAAAATTAATCGTCAAGTAACTTTTTCAAAGAGACGATCTGGTTTACTTAAGAAAGCTCATGAGATCTCTGTGCTTTGCGATGCGGAAGTTGGACTCATTGTTTTTTCAACTAAAGGAAAACTCTTTGAGTAT---TCCACTGACTCTTGCATGGAAAGGATACTTGAAAGGTATGAAAGGTACTCATATGCTGAGAGGCAACTTAATGCTACTGATATCGAAACC---CCGGGGAGCTGGACTTTGGAACATGCTAAACTTAAGGCCAGACTTGAGGTTTTGCAAAGAAACCAAAAGCATTATGCAGGAGAAGAGTTGGACACATTGAGTATGAAAGAACTTCAGAATCTGGAGCACCAGCTCGATTCTGCTCTTAAGCATATTCGATCACGAAAGAACCAATTGATGCATGAATCCATTTCTGAGCTGCAAAAGAAGGACAAGGCATTGCAAGAACAAAACAACAATCTTTCAAAGCAGGTGAAGGAAAGGGAGAAAGAGATGGCCCAGCAGACTCCGTGGGAGCAACAGAGTCAGGATCATCTCAATTCATCTTCATTCATTTTGCCACACCCTTTTAACAACCTTCACCTAGGGGAAGCATACCCAACTGCAGGAGACAATGGAGAAGTTGAAGGATCGTCACGGCAGCAACAACAAAACGTGATGCCACCATGGATG

BrunfelsiaFUL2 ATGGGAAGAGGAAGAGTTCAGCTGAAGAGAATTGAGAACAAAATCAATCGACAAGTTACGTTTTCGAAACGTCGATCTGGTTTGTTGAAAAAAGCTCATGAAATTTCAGTACTTTGTGACGCTGAAGTTGGATTAATTGTTTTTTCCACTAAAGGCAAACTCTTTGAGTAT---TCAAATGATTCTTGCATGGAAAGGATTCTTGAGAGGTATGAAAGATACTCATATGCTGAGAGGCAGCTTAATGCTACTGATCATGACACC---CCGGGGAGCTGGACACTGGAACATGCTAAGCTTAAGGCCAGACTTGAAGTTTTGCAAAGAAATCATAAGCACTATGCGGGGGAACACTTGGACTCATTAAGTATGAAAGAGCTTCAGAATCTGGAGCATCAGCTCGATTCTGCTCTTAAACAAGTTCGATCAAGAAAGAACCAATTGATGCACGAATCCATTACTGAGCTACAAAAGAAGGACAAGGCGTTGCAAGAACAAAACAACAAGCTCTCTAAGCAGGTGAAGGAAAGGGAGAAAGAGCTAGCCCAGCAGAGTCAGTGGGAGCAACAAAGCCATGAT---CTCAACTCATCTTCATTCGTTCTGACACAGCCCTTGAACTCTCTTCACATTGGTGAAGCATACCCAACAACAGGAGACAATGGAGAAGTTGAAGGATATTCGCGGCAACAACCTCAAAACGTGATGCCCCCATGGATG

Brugmansia_suaveolensFUL2 --------------------------------------------------------------------------------------------TGCGCTTGAGATCTCTGTGTTGTGTGATGCTGAGGTTGGTTTGATTGTTTTTTCCACTAAAGGAAAACTCTTTGAGTAC---TCTACTGATTCTTGCATGGAAAGGATTCTTGAAAGGTACGAACGATACTCATATGCTGAGAGGCAGCTCAATCCTACTGATCAGGACTCC---CCGGCGAGCTGGACTCTGGAGCATGCTAAGCTTAAGGCTCGAATTGAGGTTTTGCAAAGAAACCAAAGGCATTATGCGGGGGAAGACCTGGACCCTCTAAGTATGAAAGAGCTTCAGAATCTGGAGCATCAGCTCGATTCTGCTCTCAAACATATTCGATCAAGAAAGAACCAATTGATGCATGAACCTATTTCTGAGCTGCAAANGAAGGACAAGGCATTGCAAGAGCAAAACAACAAGCTTTCGAAGCAGGTAAAGGAAAGAGAAAAAGAGATTGCTCAGCAGAATCAGTGGGAGCAACAAAACCATGATCATCTCAACTCATCTTCATTTGTGTTGTCACAGCCTATGAACTCTCTTCACATTGGGGAAGCATACCCGACTGCTGGAGACAATGGAGAAATTGAAAGATCTTCGCGGCAACAACCACCAAAC------------------

Cestrum_diurnumFUL2 ------------------------------------------------------------------------------------------------------ATCACTGTGCTTTGTGATGCTGAAGTTGGTTTGATTGTTTTTTCCACTAAAGGCAAACTCTTTGAGTAC---TCTACTGATTCTTGCATGGAAAGGATTCTTGAAAGGTATGAAAGGTACTCTTATGCTGAGAGGCAGCTCAATCCTACTCAT---GACACC---CCGGGTAGCTGGATTCTGGAACATGCTAAGCTTAAGGCTAGACTTGAGGTTTTGCAAAGAAACCAAAGGCATTATGCCGGAGAAGACTTGGACTCATTAAGTACGAAGGAACTTCAGAATCTGGAGCACCAACTTGATTCTGCTCTCAAACACATTCGATCAAGAAAGAACCAATCGATGCATGAATCAATCTCAGAACTGCAAAAAAAGGAGAAGGCATTGCAAGAGCAAAACAACAAACTCTCAAAACAGGTAAAGGAAAGGGAGAAAGAGCTGGCTCAGCAGAATC--------------------------------------------------------------------------------------------------------------------------------------------------------------------

Atropa_belladonnaFUL2 ATGGGGAGAGGAAGAGTACAGTTGAAGAGGATTGAGAACAAAATTAATCGGCAAGTGACCTTCTCGAAAAGGCGATCTGGGTTGTTGAAGAAAGCKCWTGARMTSTCKGTSCTWTGTGATGCTGAAGTTGGTTTAATTGNTTTTTCAACTAAAGGCAAACTCTTTGAGTAT---TCCACTGATTCTTGCATGGAAAGGATTCTTGAAAGGTATGAAAGGTACTCATATGCTGAGAGGCAGCTTAATGCTACTGCTATCGAAACC---CCGGGGAGCTGGACTCTGGAACRTGCTAAGCTTAAGGCCAGACTTGAGGTCTTGCAAAGAAACCAAAGGCATTATGCGGGAGAAGACTTGGACTCGTYGAGTATGAAAGAGCTTCAGAATTTGGAGCACCAACTCGATTCTGCTCTTAAGCACATTCGATCAAGAAAGAACCAATTGATGCATGAATCCATTTCTGAGCTGCAAAAGAAGGACAAGGCATTACAAGAGCAAAACAACAATCTCTCAARGCAGGTGAAGGAAAGGGAGAATGAGATAGCCCAGCAGAATCAGTGGGAGCAACAAAGCCATGATCATCTCAACTCATCTTCATTCGTTATGTCACACCCCTTGAACAACCTTCACCTAGAGGAAGCATACCCGACTGCAGGAGACAATGGAGAAGTTGAAGGATCGTCGCGACAGCAACAACAAAACGTGATGCCCCCCTGGATG

Solanum_lycopersicumFUL2 ATGGGTAGAGGAAGAGTACAATTGAAGAGAATTGAGAACAAAATTAATCGTCAAGTTACTTTTTCAAAGAGGCGATCTGGTTTGCTTAAAAAAGCTCATGAGATCTCTGTGCTTTGCGATGCTGAAGTTGGACTCATTGTTTTCTCAACTAAAGGAAAACTCTTTGAGTAT---TCTACTGACTCTTGCATGGAAAGGATTCTTGAAAGGTATGAAAGGTACTCATATGCTGAAAGGCAGCTTAATGCTACTGATATTATAACC---CCGGGTAGCTGGACTTTGGAACATGCTAAGCTTAAGGCCAGACTTGAGGTTTTGCAAAGAAACCAAAAGCATTATGCAGGAGAAGAGTTGGACACATTGAGTATGAAAGAGCTTCAGAATCTGGAACACCAGCTCGATTCTGCTCTTAAGCACATTCGCTCTAGAAAGAACCAATTGATGCATGAATCCATTTCTGAGCTTCAAAAGAAGGACAAGGCATTGCAAGAACAAAACAACAATCTTTCAAAGCAGGTTAAGGAAAGGGAGAAAGAGATGGCCCAACAGACTCCGTGGGAGCAACAGAGTCATGATCATCTCAATTCATCTTCGTTTGTTTTGCCACACCCCTTTAACAATCTTCACATAGGGGAAGCATACCCAAATGCAGGAGACAATGGAGAAGTAGAAGGATCATCGCGGCAACAACAACAAAACGTGATGCCTCCATGGATG

Capsicum_annuumFUL2 ATGGGAAGAGGAAGAGTTCAATTGAGGAGGATTGAAAATAAGATAAATAGGCAAGTGACTTTTTCGAAGAGGCGATCTGGTTTGTTGAAGAAAGCTCATGAGATCTCTGTCCTTTGTGATGCTGAAGTTGGCTTGATTGTTTTTTCTTCTAAAGGGAAACTATTTGAGTAT---TCTACTGACTCTTGCATGGAAAGGATTCTTGAGAGGTATGAAAGGTACTCATATGCTGAGAGGCAGCTTAATGCAACTGATGTCGAAACC---CCGGGGAGTTGGACTTTGGAACATGCTAAGCTTAAGGCCAGGCTTGAGGTTTTGCAAAGAAACCAAAGGCATTATGCGGGAGAAGACTTGGACTCATTGAGTATGAAAGAGCTTCAGAATCTGGAGCAGCAACTCGATTCTGCTCTTAAGCACATTCGATCAAGAAAGAACCAATTGATGCATGAATCCATTTCTGAGCTGCAAAAGAAGGACAAGGCATTGCAAGAACAAAACAACAATCTTTCAAAGCAGATGAAGGAAAGGGAGAAACAGCTGGCCCAGCAGACTCCGTGGGAGCAACAGAACCATGACCATCTCAACTCATCTTCATTTGGTCTGCCACATCCCTTTAACAACAATCACCTAGGGGAAGTATATCCAACTGCAGGAGACAATGGAGAAGTTGAAGGATCATCGCGGCAGCAACAACAAAACGTGATGCCGCCATGGATG

DunaliaFUL2 ATGGGGAGAGGAAGAGTTCAGCTGAAGAGGATTGAGAACAAAATCAATAGGCAAGTCACTTTCTCCAAGAGGCGATCTGGTTTGCTAAAGAAAGCTCATGAGATCTCTGTGCTTTGTGATGCTGAAGTTGGTTTGATTGTTTTCTCAACTAAAGGCAAATTATTTGAGTAT---TCCACTGATTCTTGCATGGAAAGGATTCTTGAAAGGTATGAAAGGTACTCATATGCTGAGAGGCAGCTTAATGCTACTGATGTCGAAACC---CCGGACAGCTGGACTTTGGAACATGCTAAGCTTAAGGCCAGACTTGATGTTTTGCAAAGAAACCAAAGGCATTATGCGGGAGAAGACTTAGACTCATTGAGTATGAAAGAGCTTCAGAATCTGGAGCACCAGCTCGATTCTGCTCTTAAGCACATTCGATCAAGAAAGAACCAATTGATGCATGAATCCATTTCTCAGCTGCAAAAGAAGGACAAGGCATTGCAAGAACAAAACAACAATCTTTCAAAGCAGGTGAAGGAAAGGGAGAAAGAGCTGGTCCAGCAGACTCCGTGGGAGCAACAGAGCCATGATCATCTCAATTCATCCTCATTCGTTTTGCCACACCCCTTGAACAACCTTCACCTAGGGGAAGCATACCCAACTGCAGGAGGCAATGGAGAAGTTGAAGGATCATCGCAGCAGCACCAACAAAACGTGATGCCGCCATGGATG

Datura_inoxia_FUL2 --------------------------------------------------------------------------------------------TTCGCTTGAAATTTCGGTGCTTTGTGATGCTGAAGTTGGTTTGATTGTTTTCTCATCTAAAGGCAAACTCTTTGAGTAT---TCCACTGATTCTTGCATGGAAAGGATTCTTGAAAGGTATGAAAGGTACTCATATGCTGAGAGACAGCTTAATGCTACTGAT---GAAACC---CCGGGGAGCTGGACTTTGGAACATGCTAAGCTTAAGGCCAGACTTGAGGTTTTGCAAAGAAACCAAAAGCATTACGCAGGAGAAGACTTGGAATCATTGAGCATGAAAGAGCTTCAGAATCTGGAGCACCAGCTTGATTCTGCTCTTAAGCACATTAGATCAAGAAGGAATCAATTGATGCATGAATCAATTTCTGAGCTGCAAAAGAAGGACAAGGCATTACAAGAACAAAACAACAATCTTTCAAAGCAGGTGAAGGAAAGGGAGAAAGGACTGGCCCAGCAGACTCAGTGGGAGCAACAGAGCCATGATCATCTCAACTCATCTTCATTCATTTTGCCACACCCCTTGAACAACCTTCACCTTGGGGAAGCATACCCAACTGCAGGAGACAATGGAGAAGTTGAAGGATCGTCGAGGCAGCAACAACACAACGTGATGCCCCCCTGGATG

Lycium_barbarumFUL2 ATGGGGAGAGGAAAATTGCAACTGAAGAGGATTGAGAATAAAATAAATCGGCAAGTGACGTTCTCTAAGAGGCGATCTGGGTTGCTTAAGAAAGCTCAGGAGATCTCTGTGCTTTGTGATGCTGAAGTTGGGTTGATTGTTTTTTCAACTAAAGGCAAACTCTTTGAGTAT---TCCACCGATTCTTGCATGGAAAGGATTCTTGAAAGGTATGAAAGGTACTCATATGCTGAGAGGCGGCATAATCCTACTGATCAGGAAACC---CCGGGGAGCTGGACTCTGGAATATGCTAAGCTTAAGGCCAGACTTGAAGTTTTGCAAAGAAACCAAAGGCATTATGTGGGAGAAGACTTGGAGTCGTTAAGTATGAAGGAGCTTCAGAATCTGGAGCACCAGCTTGATTCGGCTCTGAAGCACATCCGATCAAGAAAGAACCAATTGATGCATGAATCCATTTCTGAGCTGCAAAAGAAGGACAAGGCATTGCAAGAGCAAAACAACAATCTCTCAAAGCAGGTGAAGGAAAGGGAGAAAGAGATAGCCCGGCAGAGTCAGTGGGAGCAACAGAGCCATGATCATCTCAACTCATCTTCATTCGTTTTGTCACACCCCTTGAACAACCTTCACCTAGGGGAAGCATACCCGGATGCAGGAAACCAGGGAGAAGTTGAAGGATCATCGCGGCACCAACCACAAAACGTCATGCCGCCATGGATG

Lycium_sp.FUL2 ATGGGGAGAGGAAAAGTGCAACTGAAGAGGATTGAGAATAAAATAAATCGGCAAGTGACGTTCTCTAAGAGGCGATCTGGGTTGCTTAAGAAAGCTCAGGAGATCTCTGTGCTTTGTGATGCTGAAGTTGGGTTGATTGTTTTTTCAACTAAAGGCAAACTCTTTGAGTAT---TCCACTGATTCTTGCATGGAAAGGATTCTTGAAAGGTATGAAAGGTACTCATATGCTGAGAGGCAGCATAATCCTACTGATCAGGAAACC---CCGGGGAGCTGGACTCTAGAATATGCTAAGCTTAAGGCCAGACTTGAAGTTTTGCAAAGAAACCAAAGGCATTATGTGGGAGAAGACTTGGAGTCGTCAAATATGAAGGAGCTTCAGAATCTGGAGCACCAGCTTGATTCGGCTCTGAAGCACATCCGATCAAGAAAGAACCAATTGATGCATGAATCCATTTCTGAGCTGCAAAAGAAGGACAAGGCATTGCAAGAGCAAAACAACAATCTCTCAAAGCAGGTGAAGGAAAGGGAGAAAGAGATAGCCCAGCAGAGTCAGTGGGAGCAACAGAGCCATGATCATCTCAATTCATCTTCATTCGTTTTGTCACACCCCTTGAACAACCTTCACCTAGGGGAAGCATACCCGGATGCAGGAAACCATGGAGAAGTTGAAGGATCATCGCGGCACCAATCACAAAACGTCATGCCACCATGGATG

GrabowskiaFUL2 ATGGGGAGAGGAAAAGTGCAACTGAAGAGGATTGAGAATAAAATAAATCGGCAAGTGACGTTCTCTAAGAGGCGATCTGGGTTGCTTAAGAAAGCTCATGAGATCTCTGTGCTTTGTGATGCTGAAGTTGGGTTAATTGTTTTTTCAACTAAAGGCAAACTCTTTGAGTAT---TCAACTGATTCTTGCATGGAAAGGATTCTTGAAAGGTATGAAAGGTACTCATACACTGAGAGGCAGCTTAATCCTACTGATCAGGAAACC---TTGGGGAGCTGGACTCTGGAATATTCTAAGCTTAAGGCCAGACTTGAAGTTTTGCAAAGAAACCAAAGGCATTATGCGGGAGAAGATTTGGAGTCATTAAGTATGAAGGAGCTTCAGAATCTGGAGCACCAGCTCGATTCTGCTGTGAAGCACATCCGATCAAGAAAGAACCAATTGATGCATGAATCCATTTCTGAGCTGCAAAAGAAGGACAAGGCATTGCAAGAGCAAAACAAC------------------------------------------------------------------------------------------------------------------------------------------------------------------------------------------------------------------------

Datura_metelFUL2 --------------------------------------------------------------------------------------------------------------------------------------------------------------------------------------------------------------------------------------------------------------------------------------------------------------------------------------------------------------------------------------------------------------------------------------------------------------GCATGAATCAATTTCTGAGCTGCAAAAGAAGGACAAGGCATTGCAAGAACAAAACAACAATCTTTCAAAGCAGGTAAAGGAAAGGGAGAAAGGGCTGGCTCAGCAGACTCAGTGGGAGCAACAGAGCCATGATCATCTCAACTCTTCTTCGTTCGTTTTGCCACACCCCTTGAACAACCTTCACCTTGGGGAAGCATACCCGACTGCAGGAGATAATGGAGAAGTTGAAGGATCGTTGCGGCAGCAACAACACAACGTGATGCCGCCATGGATG

Nicandra_physalodesFUL2 --------------------------------------------------------------------------------------------TGCGCATGAAATTTCGGTGCTGTGTGATGCTGAAGTTGGACTTATTGTTTTTTCTACTAAAGGAAAACTATTTGAGTAT---TCAACTGATTCTTGCATGGAAAGGATTCTTGAAAGGTATGAAAGGTACTCATATGCTGAGAGGCAGCTTAATGCTACTGAGCTCGAAACC---CCGGGGAGCTGGACTTTGGAACATGCTAAGCTTAAGGCCAGACTTGAAGTTCTTCAAAGAAACCAAAGGCATTATGCGGGAGAAGATTTGGATTCATTAAGTATGAAAGAGCTTCAGAATCTGGAGCACCAGCTCGATTCTGCTCTTAAGCACATTCGATCAAGAAAGAACCAATTGATGCATGAATCCATTTCTGAGCTGCAAAAGAAGGACAAGGCATTGCAAGAGCAAAACAACAATCTTTCAAAGCAGGTGAAGGAAAGGGAGAAAGAGATGGCCCAGCAGAGTCAATGGGAGCAACAGAGTCATGATCATCTCAATTCATCTTCATTCGCTTTGTCACACCCCTTGAATAACCTTCACCTAGGAGAAGCATACCCACCTGCAGGAGACAATGGAGAAATCGAAGGATCGTCAAGGCAGCAACAACAAAACGTGATGCCCCCCTGGATG

Nicotiana_obtusifoliaMBP10 -------------------------------------------------------------------------------------------------------------------------------------------------------------------------------CCACCGGATCCAGCATGGAAAGTATCCTCGAAAGATACGAAAGTTATTCATATGCTGAGAGGAAGTTGAATGCAAATGACTCTGAACCT---AAGGAAAACTGGACTCTGGAGTACCCAAAGCTCATGTCAAGGATTGAACTTCTCCAAAGAAATATAAGGCATTATATGGGAGAGGATTTGGGTACCTTCGGTCTGCGAGAGTTTGATGGTTTGGAGCAACAACTCGATACAGCTTTGAAGCGAATACGCACCAGGAAGAACCAACTGATGCATGAGTCCATTTCCCAGCTACGGAAAAAGGAAAAAGAGCTGCAAGAGCAAAACCACTTAATGTCGAAGAAGCTGAAAGGAAATGAGAAG---------------------------------------------------------------------------------------------------------------------------------------------------------------------------------------

Nicotiana_tomentosiformisMBP10 -------------------------------------------------------------------------------------------------------------------------------------------------------------------------------CCACCGAATCCAGCATGGAAAGTATCCTCGAAAGATACGAAAGTTATTCATATGCTGAGAGGAAGTTGAATGCAAATGACTCTGAACCT---AAGGAAAACTGGACTCTGGAGTACCCAAAGCTCATGTCAAGGATAGAACTTCTGCAAAGAAATATAAGGCATTATATGGGAGAGGATCTGGATTCCTTCGGTCTGCGGGAGTTTCATGGTTTAGAGCAACAGCTTGATACAGCTTTGAAGCGAATACGAACTAGGAAGAATCAACTGATGCATGAGTCCATTTCCCAACTGCAGAAAAAGGAAAAAGAGTTGCAAGAGCAAAACCACTTAATGTCGAAGAAGCTGAAAGGAAATGAAAAG---------------------------------------------------------------------------------------------------------------------------------------------------------------------------------------

BrunfelsiaMBP10 ATGGGAAGGGGTAAGGTTCAATTGAAGAGGATCGAAAACAAGATTAGCAGGCAAGTTACTTTCTCAAAGAGACGCTCCGGTTTGTTGAAGAAAGCTCATGAGATCTCAGTCTTGTGTGATGCGGATGTTGCTTTGATTGTCTTCTCTGCAAAAGACAAGCTCTTTGAGTAC---TCCACTGAATCTGGCATGGAAAATATCCTGGAAAGATACGAAACATACTCATACGCCGAGAGGAAGCTGAATGCGAATGACTCTGAACCTAATGAGGTAAACTGGAATCTTCAGTACCAAAAGCTCATGGCAAGGAATGAACTTCTGCAAAAAAATATAAGGCATTATATTGGAGAGGATTTGGATTCCCTCGGTATGCGAGAGTTTCAAGGTTTAGAGCAACAGCTCGATACAGCTTTGAAGCGAATACGAACAAGGAAGAACCAACTGATGCATGATTCCATTTCCCAGCTGCAGAAAAAGGAAAAAGAGCTGCAAGAGCAAAAGAACTTGATGTCGAAGAAGCTGAAAGAAAATGAGAAA---------------------------------------------------------------------------------------------------------------------------------------------------------------------------------------

Solanum_ptychanthumMBP10 --------------------------------------------------------------------------------------------------------------------------------------------------------------------------------------ATCCAGTATGGAAAATATACTGGAAAGATATGAAAGTTACTCATATGCGGAGAGGAACTTGAAT------------------TATAAGGAAAAGTGGAGTCTCGAGTACCCAAAGCTCACGGCTAGGGTTGAACTTCTGCAAAGAAATATAAGGCATTTTATGGGAGAAGATCTGGACGCCTTTAATTTGCGTGAGTTTCAGGGTTTAGAGCAACAGCTCGATACCGCTCTGAAGCGAGTACGAACTAAGAAGAATCAACTGATGCATGAGTCCATTTCCCAGCTGCAGAAAAAGGAAAAAGAACTGCAAGAGCGAAACAACTTAATTTCCAAAAAGCTTAAAGAAAATGAGAAG---------------------------------------------------------------------------------------------------------------------------------------------------------------------------------------

Solanum_tuberosumMBP10 ------------------------------------------------------------------------------------------------------------------TGTGATGCTGACGTGGCATTAATTGTCTTCTCTTCAAATGGCAAGCTCTTTGAGTAC---TCCACTCAATCCAGCATGGAAAATATATTGGAAAGATATGAAAGTTACTCATCTGCGGAAAGGAACTTGAAT------------------TATAAGGAAAACTGGACTCTCGAGTACCCAAAGCTCATGGCAAGAGTTGAACTTCTGCAAAGAAATATAAGGCATTTTATGGGAGAAGATCTGGATGCCTTTAATCTGCGTGAATTTCAGGGTTTAGAGCAACAACTCGATACAGCTCTGAAACGAGTGCGATCTAGGAAGAATCAACTGATGCATGAGTCCATTTCCCAGCTGCAGAAAAAGGAAAAAGAACTGCAAGAGCGAAACAACTTAATTTCTAAGAAGCTTAAAGAAAATGAGAAG---------------------------------------------------------------------------------------------------------------------------------------------------------------------------------------

Solanum_lycopersicumMBP10 ATGGGGCGGGGTAGGGTGGAGATGAAGCGTATCGAAAATAAAATAAGCAGACAAGTTACATTCTCAAAGAGACGATCCGGTTTGTTGAAGAAAACCAACGAGATCTCTGTGCTATGTGATGCTGAGGTGGCATTAATTGTTTTCTCTTCAAATGGAAAACTATTTGAGTAC---TCTACTCAATCAAGCATGGAAAATATATTGGAAAGATATGAAAATTACTCATACGAGGAGATGAACTTGAAT------------------TATAAGGAAAATTGGACTCTTGAGTACCCAAAGCTCATGGCAAGAGTTGAACTTCTGCAAAGAAATATAAGGCATTTTATGGGAGAAGATCTGGACGCCTTTAATCTGCGTGAATTTCGGGGTTTAGAGAAACAGCTCGATACAGCTCTAAAGCGAGTGCGATCTAAGAAGAACCAACTGATGCACGAGTCCATTTCCCAGCTGCAGAAAAAGGAAAAAGAACTGCAACAGCGAAACAACTTAATTTCTAACAAGCTTAAAGAAAATGAGAAG---------------------------------------------------------------------------------------------------------------------------------------------------------------------------------------

Solanum_pimpinellifoliumMBP10 ATGGGGCGGGGTAGGGTGGAGATGAAGCGTATCGAAAATAAAATAAGCAGACAAGTTACATTCTCAAAGAGACGATCCGGTTTGTTGAAGAAAACCGACGAGATCTCTGTGCTATGTGATGCTGAGGTGGCATTAATTGTTTTCTCTTCAAATGGAAAACTATTTGAGTAC---TCTACTCAATCAAGCATGGAAAATATATTGGAAAGATATGAAAATTACTCATACGAGGAGATGAACTTGAAT------------------TATAAGGAAAATTGGACTCTTGAGTACCCAAAGCTCATGGCAAGAGTTGAACTTCTACAAAGAAATATAAGGCATTTTATGGGAGAAGATCTGGACGCCTTTAATCTGCGTGAATTTCGGGGTTTAGAGCAACAGCTCGATACAGCTCTAAAGCGAGTGCGATCTAAGAAGAACCAACTGATGCACGAGTCCATTTCCCAGCTGCAGAAAAAGGTAAAAGAACTGCAACAGCGAAACAACTTAATTTCTAACAAGCTTAAAGAAAATGAGAAG---------------------------------------------------------------------------------------------------------------------------------------------------------------------------------------

Jaltomata_procumbensMBP10 --------------------------------------------------------------------------------------------TGCGCTTGAACTTTCGATACTTTGTGATGCTGAGGTGGCATTGATTGTTTTCTCCCCCAATGGAAAGCTCTTTGAGTAC---TCCACTGAATCCAGCATGGAAAATATACTGGAAAGATACGAAAATTACTCATATGCGGAGAGGAAGTTGAATGGAAATGATTCTCAAACTTATAAGGAAAACTGGACTCTAGAGTACCCAAAGCTCATGGCAAGGGTTGAACTTCTTCAAAGAAATATAAGGCATTTTATGGGAGAGGATCTGGATGCCTTCAATCTGCGAGAGTTTCAGGGTTTAGAGCAACAACTCGATACAGCTCTCAAGCGAATACGAACCAGGAAGAATCAACTGATGCATGCGTCCATTTCCCTGCTGCAGAAAACGGAAAAAGAACTGCAAGAGCGAAACAACTTAATTTCCAAGAAGCTTAAAGAAAATGAGAAG---------------------------------------------------------------------------------------------------------------------------------------------------------------------------------------

Juanalloa_mexicanaMBP10 --------------------------------------------------------------------------------------------TGCGCATGAACTGTCTGTGCTGTGTGATGCTGAGGTGGCATTGATTGTCTTCTCCCCCAAGGGCAAGCTCTTTGAGTAC---TCCACTGAATCCAGCATGGAAAATATACTGGAAAGATACGAAAGTTACTCATATGCAGAGAGGAAGTTGAATACAAATGACTCTCAAACTTATAAGGAAAACTGGACGCTAGAGTACCCAAAGCTCCTGGCAAGGGTTGAACTTCTGCAAAAAAATATAAGGCATTTTATGGGAGAGGATCTGGATGCCTTCAATCTGCGTGGGTTTCAGGGTTTAGAGCAACAGCTCGATACAGCTCTGAAGCGAATACGAACCAGGAAGAACCAACTGATGCATGAGTCCATTTCCCTGCTGCACAAAAAGGAAAAAGAACTGCAAGAGCGAAACAACTTAATTTCCAAGAAGCTTAAAGAAAATGAGAAG---------------------------------------------------------------------------------------------------------------------------------------------------------------------------------------

Nicandra_physaloidesMBP10 --------------------------------------------------------------------------------------------TGCGCTTGAACTGTCAATTTTGTGTGATGCTGATGTGGCATTGATTGTCTTCTCCTCCAATGGCAAGCTCTTTGAGTAC---TCCACTCAGTCCAGCATGGAAAGTATCCTGGAAAGATATGAAAGTTACTCGCATGGGGAGAGGAAATTGAATGCAAATGATTCTCAAACTTATAAGCCAAACTGGGCGCTCGAGTTCCCAAAGCTCATGTCGAGGGTTGAACTTCTCCAAAGAAATATAAGGCATTTTATGGGAGAGGATTTGGATGCCTTCAATCTGCGTGAGTTTCAGAGTTTAGAGCAACAAATTGATACAGCTCTGAAACGAATACGAACCAAGAAGAACCAACTGATGCATGAGTCCATTTCCCTACTGCAGAAGAGGGAAAAAGAACTGCAAGAGAGGAACAACTTAATTTCCAAGAAGCTTAAAGAAAATGAGAAG---------------------------------------------------------------------------------------------------------------------------------------------------------------------------------------

Brugmansia_suaveolensMBP10 ----------------------------------------------------------------------------------------------------AGATGTCGGTGTTGTGTGATGCTGAGGTGGCATTGATTGTCTTCTCCCCTAAAGGCAAGCTCTTTGAGTAC---TCCACTCAATCCAGCATGGAAAATATGCTGGAAAGATACGAAAGTTACTCCTATGCG---------------------------CAAACTTCTAAGGAAAACAGGACGCTGGAGTACCAAAAGCTCACGGCAAGGGTTGAACTTCTGCAAAGAAATATAAGGCATTTTATGGGAGAGGATCTGGATGCCTTCAATCTGCGAGAGTTTCAGGGTTTAGAGCAACAGATTAATACAGCTCTGAAGCGAATACGAACCAGGAAGAACCAACTGATGCTTGAGTCCATTTCCCTGCTGCAGAGAAAGGAAAAAAGACTGCAAGAGCAAAACAACTTAATTTCCAAGAAGCTTAAAGAATATGAGAAG---------------------------------------------------------------------------------------------------------------------------------------------------------------------------------------

Solandra_maximaMBP10 ---------------------------------------------------------------------------------------------------------TCTGTGTTGTGTGATGCTGAGGTGGCATTGATTGTCTTCTCCCCCAAAGGCAAGCCCTTTGAGTAC---TCCACTGAATCAAGCATGGAAAATATACTGGAAAGATACGAAAGTTACTCATATGCGGAGAAGAAGTTGAATGCTAATGACTCTCAAACTTATAAGGAAAACTGGACACCAGAGTACCCAAAGCTCATGGCAAGGGTTGAACTTCTGCAAAAAAATATAAGTCATTTTATGGGAGAGGATCTGGATGCCTTCAATCTGCGTGAGTTTCAGGATTTAGAGCAACAGCTCGATACAGCTCTGAAGCGAATACGAACCAGGAAGAACCAACTGATGCATGAGTCCATTTCTCTGCTGCAGAAAAAGGAAAAAGAACTGCGAGAGCGAAACAACCTAATTTCCAAGAAGCTTAAAGAAAACGAGAAG---------------------------------------------------------------------------------------------------------------------------------------------------------------------------------------

Datura_metelMBP10 -------------------------------------------------------------------------------------------------------------------------------------------------------------------------------------------------------------------------------------------------------------------------CTAAGGAAAACAGGACGCTGGAGTACCAAAAGCTCACGGCAAGGGTTGAACTTCTGCAAAGAAATATAAGGCATTTTATGGGAGAGGATCTGGATGCCTTCAATCTGCGAGAGTTTCAGGGTTTAGAGCAACAGATTAATACAGCTCTGAAGCGAATACGAACCAGGAAGAACCAACTGATGCTTGAGTCCATTTCCCTGCTGCAGAGAAAGGAAAAAAGACTGCAAGAGCAAAACAACTTAATTTCCAAGAAGCTTAAAGAATATGAGAAG---------------------------------------------------------------------------------------------------------------------------------------------------------------------------------------

Atropa_belladonnaMBP10 -------------------------------------------------------------------------------------------------------------------------------------------------------------------------------CCACTGAGTCCAGCATGGAAAGTATCCTGGAAAGATATGAAAGTTACTCACATGCGGAGAGGAAGTTGAATGCAAATGACTCTCAAACTTATAAGGAAAATTGGACTCTCGAGTACCCAAAGCTCATGGCAAGGACTGAACTTCTGCAAAGAAATATAAGGCATTTTATGGGAGAGGATCTGGATTCCTTCAATCTGCGAGAGTTCCAGGGTTTAGAACAACAGCTCGATACAGCTCTGAAGCGAATACGAACCAGGAAGAACCAACTGATACATGAGTCCATTTCCCAGCTGCAGAAAAAGGAAAAAGAGCTGCATGAGCGAAACCACTTAATTTCCAAAAAGCTGAAAGAAAATGAGAAG---------------------------------------------------------------------------------------------------------------------------------------------------------------------------------------

Lycium_barbarumMBP10 ----------------------------------------------------------------------------------------------------------------------------------------------------------------------------------------------------------------------------------------------------------------------------AGGAAAATTGGACTCTCGAGTACCCAAAGCTCAGGGCAAGGACTGAACTTCTGCAAAGAAATATAAGGCATTTTATGGGAGAGGATCTGGATACCTTCAATCTGCGAGAATTTCAGGGTTTAGAGCAACAGCTCGATACAGCTCTCAAGCGAATACGAACCAGGAAGAACCAACTGATGCATGAGTCCATTTCCCAGCTGCAGAAAAAGGAAAAAGAGCTGCAGGACCGAAACAACTTAATTTCCAAGAAGCTGAAAGAAAATGAGAAG---------------------------------------------------------------------------------------------------------------------------------------------------------------------------------------

DunaliaMBP10 ---------------GTTGAGATGAAGCGGATCGAGAACAAAATAAGCAGGCAAGTGACTTTCTCGAAGAGACGATCCGGTTTGTTGAAGAAGACTCATGAGATCTCCGTGTTGTGTGATGCTGAAGTGGCATTGATTGTCTTCTCCTCCAGTGGCAAGCTCTTTGAGTAC---CCTACTCAATCCAGCATGGAAAGTATCCTGGAAAGGTACGAAAATTACTCATATGCGGAGAGGAAGTTGAATGCAAATGACACCGAAACTAATAAGGAGAACTGGACGCTCGAGTACCCAAAGCTCATGGCAAGGGTGGAACTTCTGCAAAGAAATATAAGGCATTNTATGGGAGAGGATCTGGATGCCTTCAACCTGCGTGAGTTTCAGAGTTTAGAGCAACAGCTCGATACAGCTCTCAAGCGAATACGAACCAGGAAGAACCAACTGATGTTCGAGTCCATTTCCCTGCTGCAGAAAAAGGAAAAAGAAATGCAAGAGCGAAACAACTTAATTTCCAAGAAGCTTAAAGAAAATGAGAAG---------------------------------------------------------------------------------------------------------------------------------------------------------------------------------------

Nicotiana_sylvestrisMBP10 -------------------------------------------------------------------------------------------------------------------------------------------------------------------------------CCACTGAATCCAGCATGGAAAGTATCCTCGAAAGATACGAAAGTTACTCATATGCTGAGAGGAAGTTGAATGCAAATGACTCTGAACCT---------------------GAAAAACCAAAGCTCATGTCAAGGATTGAACTTCTACAAAGAAATATAAGGCATTATATGGGAGAGGATCTGGATTCCTTCTGTCTGCGAGAGTTTCATGGTTTAGAGCAACAACTTGATACAGCTNTGAAGCGAATACGCGCCAGGAAGAACCAACTGATGCATGAGTCCATTTCCCAG---------------------------------------------------------------------------------------------------------------------------------------------------------------------------------------------------------------------------------------------------------------

Withania_sominferaMBP20 ---------------------------------------------------------------------------------------------GCGCTTGAGATGTCAGTTTTCTGTGATGCTGATGTTGCTTTGATTGTTTTCTCTACCAAAGGCAAGCTCTTTGAGTTCTCT---ACTGACTCCAGTATGGAAAGTATTCTGGAAAGATATGAAAGATACTCATATGCAGATAGAAAGATGAATGCAAATGACATTGATCCC---AAGGAAAATTGGAATGTGGAGTATCCGAAACTCATGTCAAGGATTGAACTCTTACAAAGAAATATAAGGCATTATATGGGTCAGGATCTTGACCCTCTCAGTTTGCGAGAGATCCAGAGCTTAGAGCAACAGATTGATACTTCATTAAAGAGAATAAGAAGCAGGAAGAACCAGCTGATGCATGAGTCCATCTCTGAGCTGCAGAAAAAGGAGAAAGCGGTACAAGAACAAAATAACTTGATAACTAAGAAGCTGAAAGAAAAGGAGAAG---------------------------------------------------------------------------------------------------------------------------------------------------------------------------------------

Iochroma_fuchsiodeasMBP20 --------------------------------------------------------------------------------------------TGCGCTTGAGCTGTCGGTGCTATGCGATGCTGATGTTGCTTTGATTGTTTTCTCTACCAAAGGCAAGCTCTTTGAGTACTCC---ACTGACTCCAGTATGGAAAGTATTCTGGAAAGATATGAAAGATACTCACATGCAGAGAGAAAGATGAATGCAAATGACTCTGATCCC---AAGGAAAATTGGAATGTGGAGTATCCGAAGCTCATGTCAAGGATTGAACTTTTACAAAGAAATATAAGGCATTTTATGGGTCAGGATCTTGACCCTCTCAGTTTGCGAGAGCTCCAGAGTTTAGAGCAACAGATTGATACTTCATTAAAGCGAATAAGAAGCAGGAAGAACCAGCTGATGCATGAATCCATTTCTGAGATGCAGAAAAAGGAGAAAGCGATGCAAGAACAAAACAACTTGATAACTAAGAAGCTGAAAGAAAAGGAGAAG---------------------------------------------------------------------------------------------------------------------------------------------------------------------------------------

Nicandra_physalodesMBP20 --------------------------------------------------------------------------------------------------TGAGCTGTCGATGTTGTGCGATGCTGATGTTGCTTTGATTGTTTTTTCTACCAAAGGCAAACTCTTTGAGTACTCCTCCACTGAATCCAGCATGGAAAGTATTCTGGAAAGATACGAAAGATATTCATATGCAGAGAGAATGTTGAATGCAAATGACGACGATCCA---AAGGATAATTGGAGTGTGGAGTATCCGAAGCTCATGTCAAGGATTGAACTCTTACAAAGAAATATAAGGCATTATATGGGTCAGGATTTGGATCCTCTCAGTTTGCGAGAGCTCCAGAGTTTAGAGCAACAGATTGATACTTCATTGAAGCGAATACGAAGCAGAAAGAACCAACTAATGCATGAGTCTATTTCGGAGCTGCAGAAAAAGGAGAAAACACTGCAAGAACAAAACAGCTTGATAAGTAAAAAGCTGAAAGAACACAACAAG---------------------------------------------------------------------------------------------------------------------------------------------------------------------------------------

DunaliaMBP20 ATGGGAAGAGGGAGGGTAGAGTTGAAGCGGATCGAGAACAAGATAAGCAGACAAGTGACATTCTCAAAGAGACGATCTGGATTGTTGAAGAAAGCTAATGAGATCTCCGTTCTCTGTGATGCTGATGTTGCTTTGATTGTTTTCTCTACCAAAGGCAAGCTCTTTGAGTACTCC---ACTGACTCCAGTATGGAAAGTATTCTGGAAAGATATGAAAGATACTCATATGCAGAGAGAAAGATGAATGCAAATGACTCTGATCCC---AAGGAAAATTGGAATGTGGAGTATCCGAAGCTCATGTCAAGGATTGAACTTTTACAAAGAAATATAAGGCATTTTATGGGTCAGGATCTTGACCCTCTCAGTTTGCGAGAGCTCCAGAGTTTAGAGCAACAGATTGATACTTCATTAAAGCGAATAAGAAGCAGGAAGAACCAGCTGATGCATGAATCCATTTCTGAGCTGCAGAAAAAGGAGAAAGCGATGCAAGAACAAAACAACTTGATAACTAAGAAGCTGAAAGAAAAGGAGAAG---------------------------------------------------------------------------------------------------------------------------------------------------------------------------------------

Schizanthus_grahamiiMBP20 ------------------------------------------------------------------------------------------------------------------TGCGATGCTGAGGTTGCTTTGGTCGTCTTCTCCACTAAAGGAAAGCTCTTTGAGTACTCC---ACTGACTCCAGAATGGAAAGGATTATGGAAAGATATGAAAGATACTCATATGCTGAGAGAAAGTTGAATGCAGATGACTCTGAACCC------------TGGACTCTGGAGTACCCCAAGCTCACGGCAAGGATGGAACTTCTACAAAGAAACATGAGGAATTATATGGGTGAGGATCTGGACCCTCTCAGTTTGCGAGAGTTTCAGAGTTTAGAGCAACAACTTGATACGGCTTTGAAACGAATACGAACCAGGAAGAATCAACTGATGCGTGAGTCCATCTCTGAACTGCAGAAAAAGGAGAAAACGCTGCAAGAACAAAACAACTTTATGACTAAGAAGCTCAAAGAAGATGAGAAG---------------------------------------------------------------------------------------------------------------------------------------------------------------------------------------

StreptosolenMBP20 ATGGGAAGGGGTAGGGTTGAGCTGAAGCGGATCGAGAACAAAATAAGCAGGCAAGTGACTTTCTCGAAAAGGCGTAGCGGATTGTTGAAGAAAGCACATGAGATCTCAGTTCTGTGTGAAGCTGAGGTTGGTTTGATTGTTTTCTCCACTAAAGGCAAGCTCTTTGAGTACTCC---ACTGAATCCAGCATGGAAAATATTCTGGAACGATACGAAAGATACTCATATGCAGAAAGGAAGTTGAATGGAAATGACTCTGATCCC---AAGGAAAATTGGAGTTTGGAGTACCCGAAGCTTATGTCAAGGGTTGAACTTATACAAAGAAATATGAGGCATTATATGGGTCAGGATCTGGACCCTCTCAGTTTGCGGGAGCTGCAGAGTTTGGAGCAACAGGTTGATACTGCTTTGAAGCGAATACGCACCAGGAAGAACCAAGTGATGCACGAGTCCATATCTGAGCTGCAGAAAAAGGAGAAAGCACTGCATGAACAAAACAACCTGATGACTAAGAAGTTGAACGAAAAGGAGAAG---------------------------------------------------------------------------------------------------------------------------------------------------------------------------------------

Goetzia_sp.MBP20 ---------------------------------------------------------------------------------------------GCGCTTGAAATATCGGTTTTTTGTGATGCTGAAGTTGCTTTGATCGTATTCTCTTCCAAAGGCAAGCTCTTTGAGTACTCC---ACTGAATCCAGCATGGAAAGTATTCTGGAGAGATACGAAAGATACTCATATGCTCAGAGAAAGCACAATGCTAATGATTCTGATCCC---GGGGAAAATTGGACCATGGAGTACCCGAAGCTCATGTCAAGGATTGAACTTCTACAAAGAAATATAAGGCATTATATGGGTGAGGATCTGGACCCTCTCAGTTTGCGAGAGATTCAGAGTTTAGAGCAACAAATTGATACAGCTTTGAAGCGAATACGAAGCAGGAAGAACCAACTTATGCACGAGACCATTTCTGAACTACAGAAAAAGGAGAAAGCGCTGCAAGAGCAAAACAACTTAATAACCAAGAAGCTGAAAGAAAAAGAAAAG---------------------------------------------------------------------------------------------------------------------------------------------------------------------------------------

Mandragora_officianarumMBP20 --------------------------------------------------------------------------------------------------------GTCGGTGCTGTGTGATGCTGACGTTGCTTTGATTGTTTTCTCTACCAAAGGCAAGCTCTTTGAGTACTCT---ACTGACTTCAGTATGGAAAGTATTCTGGAAAGGTGCGAAAGATACTCATATGCAGAAAGAAAGTTGAACGGAAATGAGTCTGATCCC---AAGGAAAATTGGAGTGTGGAGTATCCCAAGCTCATGTCAAGGATTGAACTTTTACAAAGAAAAATAAGGCATTATATGGGTCAGGATCTGGACCCTCTCAGTTTGCGAGAGCTCCAGAGTTTAGAGCAACGGATTGATACTTCATTAAAGCGAATAAGAAGCAGGAAGAACCATCTGATGCACGAGTTCATTTCTGAGTTGCAGAAAAAGGAGAAGGCGCTGCACGAACAAAACAACTTGATAACTAAGAAGCTGATTGAAAGCGAGAAG---------------------------------------------------------------------------------------------------------------------------------------------------------------------------------------

Plowmania_nyctaginoidesMBP20 --------------------------------------------------------------------------------------------TGCGCTTGAACTTTCCATGTTTTGTGATGCTGATGTTGCTTTAATTGTTTTCTCAACTAAAGGCAAGCTATTTGAGTACTCC---TCTGAGTCCAGTATGGAAAGCATTCTGGAAAGGTATGAAAGATACTCATATGCAGAGAGAAAGGTGAATCCCAATGACTCTAATCCC---CAGGAAAATTGGACATTGGAGTACCCTAAGCTTATGTCAAGGATTGAACTTGTACAAAGAAATATAAGGCATTATATGGGTCAGGACCTGGACCCTCTCAGTTTGCGAGAGCTGCAAAATCTAGAGCAACAGATTGACACTGCATTGAAGCGAATACGCAGCAGGAAGAATCAACTGATGCACGAGTCCATTTCTGAGCTGCATAAAAAGGAGAAAGCATTGCAAGAACAAAATAACTTGATGACTAAGA-----------------------------------------------------------------------------------------------------------------------------------------------------------------------------------------------------------

Nicotiana_obtusifoliaMBP20 ATGGGAAGAGGTAGGGTTCAGCTGAAGCGGATCGAGAACAAGATCAGCAGGCAAGTCACCTTCTCAAAGAGGCGTTCTGGATTGTTGAAGAAAGCAAATGAGATCTCCGTTTTGTGTGATGCTGATGTTGCCTTGATTGTTTTCTCCACCAAAGGCAAGCTCTTTGAGTACTCA---TCCGAGTCCAGCATGGAAAGTATTCTGGAAAGATATGAAAGATACTCATATGCAGAGAGAAAGTCAAATGCAAATGACTCTGATCCC---ATGGAAAATTGGACTCTGGAGTACCCGAAACTCATGTCAAGGATTGAACTTATACAAAGAAACATAAGGCATTATACGGGCCAGGATCTGGACCCTCTTAGTTTGAGAGAGCTACAGAGTTTAGAGCAACAGATTGATACAGCATTGAAGCGAATACGAAGCAGGAAGAACCAACTGATGCACAAGTCCATTTCTGAGCTGCAGAAAAAGGAGAAAGCACTGCAAGAACAGAACAACTCGATGACTAAGAAGCTGAAAGACAAAGAGAAG---------------------------------------------------------------------------------------------------------------------------------------------------------------------------------------

Nicotiana_sylvestrisMBP20 ----------------------------------------------------------------------------------------------------------------------------------------------------------------------------CC---TCCGAGTCCAGCATGGAAAGTATTCTGGAAAGATACGAAAGGTACTCATATGCAGAGAGAAAGTTGAATGCCAATGACGTTGATCCC---ATGGAAAATTGGACTCTGGAGTACCCGAAGCTCATGTCAAGGATTGAACTTATACAAAGAAACATAAGGCATTATACGGGCCAGGATCTGGACCCTCTTAGTTTGCGAGAGCTACAGAGTTTAGAGCAACAGATGGATACAGCATTGAAGCGAATACGAAGCAGGAAGAACCAACTGATGCACGAGTCCATTTCTGAGCTGCAGAAAAAGGAGAAAGCGCTGCAAGAACAAAACAACTCGATGACTAAGAAGCTGAAAGACGAAGAGAAG---------------------------------------------------------------------------------------------------------------------------------------------------------------------------------------

Solandra_maximaMBP20 -------------------------------------------------------------------------GTGTGCTGGAATTCGCCCTTGCGCTTGAGCTGTCGGTGCTGTGTGATGCTGACGTTGCTTTGATTGTTTTCTCTACCAAAGGCAAGCTCTTTGAGTACTCC---ACTGACTCCAGTATGGAAAGTATTCTGGAAAGATACGAAAGATACTCATATGCAGAGAGAAAGATGAATGCAAATGACTCTGATCCC---AAGGAAAATTGGAGTGTGGAGTATCCGAAGCTCATGTCAAGGATTGAACTTTTACAAAGAAATACAAGGCAATATATGGGTCAGGATCTGGACCCTCTCAGTCTGCGAGATCTGCAGAGTTTAGAGCAACTGATTCATACATCATTGAAGCGAATACGAAGCAGGAAGAACCAACTGATGCACGAGTCTATTTCGGAGCTGCAGAAAAAGGAGAAAGCGCTGCAAGAACAAAACAACTTGATAACAAAGAAGATGAAAGAAAACGAGAAG---------------------------------------------------------------------------------------------------------------------------------------------------------------------------------------

Juanulloa_mexicanaMBP20 -----------------------------------------------------------------------------------------------------------GGTGTTTTGTGATGCTGACGTTGCTTTAATTGTTTTCTCTACCAAAGGCAAGCTCTTTGAGTATTCC---ACTGACTCCAGTATGGAAAGTATTCTGGAAAGATACGAAAGATACTCATATGCAGAGAGAAAGATGAATGCAAATGACTCCGATCCG---AAGGAAAATTGTAGTGTGGAGTATCCGAAGCTCATGTCAAGAATTGAACTTTTACAAAGAAATACAAGGCAATATATGGGTCAGGATCTGGACGCTCTCAGTTTGCGAGATCTGGAGAGTTTAGAGCAACAAATTGATACATCATTGAAGCGAATACGAAGCAGGAAGAACCAACTGATGCACGAGTCTATTTCGGAGATGCAGAAGAAAGAGAAAGCGCTGCAAGAACAAAACAACTTGATAACTAAGAAGCTGAAAGAAAACGAGAAG---------------------------------------------------------------------------------------------------------------------------------------------------------------------------------------

Petunia_exsertaMBP20 --------------------------------------------------------------------------------------------TGCGCTTGAAATTTCTGTTCTGTGTGATGCTGATGTTGCTTTAATAGTTTTTTCTACCAAAGGCAAGTTATTTGAGTACTCC---ACTGAGCCCAGCATGGAAAGTATACTGGAAAGGTACGAAAGATACTCATATGCAGAGAGAAAGCTGAATGCTAATGACTCTGATCCC---AAGGAAAATTGGACACTGGAGTACCCGAAGCTCATGTCAAGAATTGAACTTATACAAAGAAATATAAGGCATTATATGGGTCAGGATCTGGACCCTCTCAGTTTGCGAGAGCTGCAGAGTTTAGAGCAACAAATTGACACAGCATTAAAGCGAATACGAAGCAGGAAGAACCAACTGATGCACGAGTCCATTTCTGAGCTGCACAAAAAGGAGAGAGCGCTGCAAGAACAAAATAACTTGATGACTAAGAAGCTGAAAGAAAATGAGAAG---------------------------------------------------------------------------------------------------------------------------------------------------------------------------------------

Petunia_hybridaMBP20 ---------------------------------------------------------------------------------------------------------------------------------------------------------------------------------------TCCAGCATGGAAAGTATACTGGAAAGGTACGAAAGATACTCATATGCAGAGAGAAAGCTGAATGCTAATGACTCTGATCCC---AAGGAAAATTGGACACTGGAGTACCCGAAGCTCATGTCAAGAATTGAACTTATACAAAGAAATATAAGGCATTATATGGGTCAGGATCTGGACCCTCTCAGTTTGCGAGAGCTGCAGAGTTTAGAGCAACAAATTGACACAGCATTAAAGCGAATACGAAGCAGGAAGAACCAACTGATGCACGAGTCCATTTCTGAGCTGCACAAAAAGGAGAGAGCGCTGCAAGAACAAAATAACTTGATGACTAAGAAGCTGAAAGAAAATGA-------------------------------------------------------------------------------------------------------------------------------------------------------------------------------------------

BrunfelsiaMBP20 ATGGGAAGGGGTAGGGTTCAGTTGAAACGAATCGAAAACAAGATCAGCAGGCAAGTCACCTTTTCCAAGAGGCGCTCAGGATTGTTGAAGAAAGCACATGAGATCTCAGTTTTATGTGATGCTGAGGTTGCCTTGATCATTTTCTCTACTAAAGGCAAGTTATTTGAGTACTCC---ACTGAGTCCAGCATGGAAAGTATCCTGGAAAGGTACGAAAGATACTCCTACGCAGAGAGAAGGTTGAATAGAGATGACTCTGATCCC---AAGGAAAATTGGACCCTGGAGTACCCGAAGCTCATGTCAAGGATTGAAATTATACAAAGAAATATAAGGCATTATACGGGTCAGGATTTGGACCCTCTCAATTTGCGAGAGCTGCAAAGTTTAGAGCAACAGATTGATACTGCATTGAAGCGAATAAGAAGCAGGAAGAACCAACTGATGCAGGAGACCATTTCTGAGCTGCATAAAAAGGAGAAATTTCTGCAAGAGCAAAACAACTTGATGACCAAGAAGCTGAAAGAAAATGAGAGG---------------------------------------------------------------------------------------------------------------------------------------------------------------------------------------

Browallia_americanaMBP20 ---------------------------------------------------------------------------------------------GCGCTTGAGATTTCTATCCTTTGCGATGCTGAAGTTGGTTTGATTGTTTTCTCCACTAAAGGCAAGCTCTTTGAGTACTCC---ACTGAATCCAGCATGGAAAATATTCTGGAACGATACGAAAGATACTCATATGCAGAAAGGAAGTTGAATGGAAATGACTCTGATCCC---AAGGAAAATTGGAGCTTGGAGTACCCAAAGCTTATGTCAAGGGTTGAACTTATACAAAGAAATATGAGGCATTATATGGGTCAGGATCTGGACCCTCTCAGTTTGCGGGAGCTGCAGAGTTTGGAGCAACAGATTGATACTGCTTTGAAGCGAATACGCACCAGGAAGAATCAAATGATGCACGAGTCCATCTCCGAGCTGCAGAAAAAGGAGAAAGCACTGCATGAACAAAACAACCTGATGACTAAGAAGTTGAACGAAAAGGAGAAG---------------------------------------------------------------------------------------------------------------------------------------------------------------------------------------

Datura_inoxia_MBP20 ---------------------------------------------------------------------------------------------GCGCTTGARATKTCKGTGTTTTGTGATGCTGACGTTGCTTTGATTGTTTTCTCTACCAAAGGCAAGCTCTTTGAGTACTCC---ACTGACTCCAGTATGGAAAGTATTCTGGAAAGATATGAAAGATACTCATGCGCAGAGAGAAAGATGAATGCAAATGACTCTGATCCC---AAGGAAAATTGGAGTGTGGAGTATCCAAAGCTCATGTCAAGGATTGAACTTTTACAAAGAAATATAAGGCATTATATGGGTCAGGATCTGGACCCTCTCGGTTTACGAGAGCTGCAGAGTTTAGAGCAACAGATTGATACTTCATTGAAGCGAATACGAAGCAGGAAGAACCAACTGATGCACGAGTCTATTTCTGAGCTGCAGAGAAAGGAGAAAGCGCTGCAAGAACAAAACAACTTGATAACTAAGAAGCTGAAAGAAAACGAGAAG---------------------------------------------------------------------------------------------------------------------------------------------------------------------------------------

Brugmansia_suaveolensMBP20 ---------------------------------------------------------------------------------------GCGCTTGAACTATCCGTTTTTTGTGATGCTGACGTTGCTTTGATTGTTTTCTCTACCAAAGGCAAGCTATTTGAGTACTCC---AATGACTCCAGTATGGAAAGTATTCTGGAAAGATACGAAAGATACTCATATGCAGAGAGAAAGATGAATGCAAATGACTCTGATCCC---AAGGAAAATTGGAGTGTGGAGTATCCAATGCTAACGTCAAGGATTGAACTTTTACAAAGAAATATAAGGCATTATATGGGTCAGGATCTGGATCCTCTTAGTTTACGAGAGCTGCAGAGTTTAGAGCAACAGATTGATACTTCATTGAAGCGAATACGAAGCAGGAAGAACCAACTGATGCACAAGTCTATTTCGGAGCTGCAGAAAAAGGAGAAAGCGATGCAAGAACAAAACAACTTGATAACTAAGAAGCTGAAAGAAAACGACAAG---------------------------------------------------------------------------------------------------------------------------------------------------------------------------------------------

Datura_metelMBP20 ------------------------------------------------------------------------------------------------------------------------------------------------------------------------------------------------------------------------------------------------------------------------------------------------------------------------------------------------------------------------CCTCTTAGTTTACGAGAGCTGCAGAGTTTAGAGCAACAGATTGATACTTCATTGAAGCGAATACGAAGCAGGAAGAACCAACTGATGCACAAGTCTATTTCGGAGCTGCAGAAAAAGGAGAAAGCGATGCAAGAACAAAACAACTTGATAACTAAGAAGCTGAAAGAAAACGACAAG---------------------------------------------------------------------------------------------------------------------------------------------------------------------------------------

Atropa_belladonnaMBP20 ATGGGAAGAGGTAGGGTAGAGTTGAAGCGGATAGAGAACAAGATAAGCAGGCAAGTGACTTTCTCAAAGAGACGATCTGGATTGTTGAAGAAAGCAAATGAGATCTCCGTTTTATGTGATGCTGATGTTGCTTTGATTGTTTTCTCTACAAAAGGCAAGCTCTTTGAGTACTCT---ACCGACTCAAGTATGGAAAGCATTCTGGAAAGATACGAAAGATACTCATATGCAGAGAGAAAGCTGAATGCAAATGACTCTGATCCC---AAGGAAAATTGGACAGTGGAGTATCCGAAGCTCATGTCAAGGATTGAACTTTTACAAAGAAATATAAGGAATTATATGGGTCAGGATCTAGACCCTCTCAGTTTGCGAGAGCTCCAGAGTTTAGAGCTCCAGATTGATACTTCATTGAAACGAATACGAAGCAGGAAGAACCAACTGATGCACGAATCCATTTCTGAGCTGCAGAAAAAGGAGAAAGCGCTGCAAGAACAAAACAACTTGATAAGTAAGAAGCTGAAAGAAAATGACAAG---------------------------------------------------------------------------------------------------------------------------------------------------------------------------------------

GrabowskiaMBP20 ------------------------------------------------------------------------------------------------------------------------------------------------------------------------------------------------------------------------------------------------------------------------------------------------------------------------------------------------------------------------------------------------------------------------------------------------------------ATGCACGAGTCCATTTCTGAGCTACAGAAAAAGGAGAAAGCGCTGCAAGAACAAAACAACTTGATAAGTAAGAAGCTGAAAGAAAAGGAGAAG---------------------------------------------------------------------------------------------------------------------------------------------------------------------------------------

Lycium_sp.MBP20 ---------------------------------------------------------------------------------------------------------------------------------------------------------------------------------------TCCAGCATGGAAAGTATTCTGGAAAGATACGAAAGATACTCATATGCAGAGAGAGAGTTGAATCCTAATGACTCTGATCCC---AAGGAAAATTGGAGTGTGGAGCGTCCGAAGCTAATGTCAAGGATTGAACTTTTACAAAGAAATATAAGGCATTATATGGGTCAGGATCTGGACCCTCTCAATTTGCGGGAGCTCCAAAGTTTAGAACAACAGATTGATACATCATTGAAGCGAATACGAACCAGGAAGAACCAACTGATGCACGAGTCCATTTCTGAGCTGCAGAAAAAGGAGAAAGCGCTGCAAGAACAAAACAACTTGATAAGTAAGAAGCTGAAAGAAAACGAGAAG---------------------------------------------------------------------------------------------------------------------------------------------------------------------------------------

Solanum_pimpinellifoliumMBP20 ATGGGAAGAGGTAGGGTAGAGTTGAAACGGATCGAGAACAAAATAAGCAGACAAGTAACATTCTCAAAGAGACGATCTGGATTATTGAAGAAAGCTAATGAGATCTCAGTATTATGTGATGCTGATGTTGCATTGATTGTGTTTTCTACCAAAGGCAAACTTTTCGAGTATTCCTCAAATGACTCAAGTATGGAAAGTATTCTTGAAAGATATGAAAGATGCTCATATGCAGAGAGACAGATGAATGCTAATGATTCTGATCCC---AAGGAAAATTGGAGTGTGGAGTATCCGAAGCTCATGTCAAGAATTGAACTTTTACAAAGAAATATAAGGCATTACATGGGTCAGGATCTGGACCCTCTCAGTTTGCGTGAGCTCCAGAGTATAGAGCAACAGATTGACACTTCATTAAAGAGAATTAGAAGCAGGAAGAATCAACTGATGCACGAGTCCATTTCTGAGCTGCAGAAAAAGGAGAAAGCGCTCCAAGAACAAAACAACTTGATTACTAAGAAGCTAAAAGAAAATGAGAAG---------------------------------------------------------------------------------------------------------------------------------------------------------------------------------------

Jaltomata_procumbensMBP20 --------------------------------------------------------------------------------------------TGCGCTTGAGCTGTCGATGCTTTGTGATGCTGATGTTGCTTTGATTGTTTTCTCTACAAAAGGCAAGCTCTTTGAGTACTGCTCAACTGACTCCAGTATTGAAAGTATTCAGGAAAGATACGAAAGATGCTCATTTGCAGAGAGAAAGATGAATGCAAATGACGCTAATCCC---AAGGAAAATTGGAGTGTGGAGTATCCGAAGCTCATGTCAAGGATTGAACTTTTACAAAGAAATATAAGGCATTATATGGGTCAAGATCTGGACCCTCTCAGTTTACGAGAGCTCCGGAGTTTAGAGCAACAAATTGATACTTCATTGAAGCGAATACGAAGCAGGAAGAACCAACTGATGCACGAGTCTATTTCGGAGCTGCAGAAAAAGGAGAAAGCGTTGCAAGACCAAAACAACTTGATGACTAAGAAGCTGAAAGAAAAGGAGAAG---------------------------------------------------------------------------------------------------------------------------------------------------------------------------------------

Solanum_quitoenseMBP20 --------------------------------------------------------------------------------------------TGCGCTTGAACTTTCTGTTTTTTGTGATGCTGATGTTGCTTTGATTATTTTTTCTACTAAAGGAAAGCTATCTGAGTATGCCTCCACTGACTCCAGTATGGAAAGTATTCTGGAAAGATACGAAAGATACTCATATGCAGAGAGAGATATGAACGCAAATGATTCTGATCCC---AAGGAAAATTGGAGTGTGGAATGTCCGAAGCTCATGTCAAGGATTGAACTTTTACAGAAAAATATAACGCATTACATGGGTCATGATCTAGACCCTCTCAGTTTACGTGAGCTCCAGAGTTTAGAGCAACAGATTGATACTTCATTAAAGAGAATTAGAAGCAGGAAGAACCAACTGATGCACGAGTCCATTTCTGAGCTGCAGAAAAAGGAGAAAGCGCTGCAAGAACAAAACAACTTGATAACTAAGAAGCTGAAAGAAAATGAGAAG---------------------------------------------------------------------------------------------------------------------------------------------------------------------------------------

Cestrum_diurnumMBP20 --------------------------------------------------------------------------------------------TGCGCTTGAGCTCTCGGTCTTTTGTGATGCTGAAGTTGCTTTGATTGTTTTCTCCACCAAAGGCAAGGTCTTTGAGTACTCGTCCACTGAATCCAGCATGGAAAGTATTCTGGAAAGATATGAAAGATACTCATACGCAGAGAAGAAGTTGAACGCCAATGACTCTGATCCC---AAGGAAAATTGGAGTCTGGAGTGCTCGAAGCTTATGTCAAGGATTGAACTTATACAAAGAAACATGAGGCACTACACGGGTCAAGATCTGGATCCCCTCGGTTTGAAAGAGCTGCAGAGCTTAGAGCAGCAGATTGATACTGCATTGAAGCGAATACGAAGCAGGAAGAACCAAATGATGCACCAGTCCATTTCTGAGCTCCAGAAAAAGGAGAAAGCGCTGCACGAACAAAACAACCTGATGACTAAGAAGTTGAAAGAATATGAGAAG---------------------------------------------------------------------------------------------------------------------------------------------------------------------------------------

Cestrum_nocturnumMBP20 ---GGAAGAGGTAGGGTTCAGTTGAAGCGGATCGAGAACAAGATCAGCAGGCAAGTTACCTTCTCTAAGAGGCGTTCTGGATTGTTGAAGAAAGCACATGAGATCTCAGTTTTGTGTGATGCTGAAGTTGCTTTGATTGTTTTCTCCACCAAAGGCAAGCTCTTTGAGTACTCGTCCACTGAATCCAGCATGGAAAGTATTCTGGAAAGATATGAAAGATACTCATACGCAGAGAAAAATTTGAACGCCAATCACTCTGATCCC---AAGGAAAATTGGAGTCTGGAGTACTGGAAGCTTATGTCAAGGATTGAACTTATACAAAGAAACATGAGGCACTATACGGGTCAAGATCTGGATCCCCTCGGTTTGAAAGAGCTGCAGAGTTTAGAGCAGCAGATTGATACTGCATTGAAGCGAATACGAAGCAGGAAGAACCAAATGATGCACCAGTCCATTTCTGAGCTCCAGAAAAAGGAGAAAGCGCTGCACGAACAAAACAACCTGATGACTAAGAAGTTGAAAGAAAATGAGAAG---------------------------------------------------------------------------------------------------------------------------------------------------------------------------------------

Solanum_lycopersicumMBP20 ATGGGAAGAGGTAGGGTAGAGTTGAAACGGATCGAGAACAAAATAAGCAGACAAGTAACATTCTCAAAGAGACGATCTGGATTATTGAAGAAAGCTAATGAGATCTCAGTATTATGTGATGCTGATGTTGCATTGATTGTGTTTTCTACCAAAGGCAAACTTTTCGAGTATTCCTCAAATGACTCAAGTATGGAAAGTATTCTTGAAAGATATGAAAGATGCTCATATGCAGAGAGACAGATGAATGCTAATGATTCTGATCCC---AAGGAAAATTGGAGTGTGGAGTATCCGAAGCTCATGTCAAGAATTGAACTTTTACAAAGAAATATAAGGCATTACATGGGTCAGGATCTGGACCCTCTCAGTTTGCGTGAGCTCCAGAGTATAGAGCAACAGATTGATACTTCATTAAAGAGAATTAGAAGCAGGAAGAATCAACTGATGCACGAGTCCATTTCTGAGCTGCAGAAAAAGGAGAAAGCGCTCCAAGAACAAAACAACTTGATTACTAAGAAGCTAAAAGAAAATGAGAAG---------------------------------------------------------------------------------------------------------------------------------------------------------------------------------------
